# Nearly half of 325 athletes reported pelvic floor symptoms: a cross-sectional study at the Lima 2024 World Athletics U20 Championships

**DOI:** 10.1136/bmjsem-2025-002564

**Published:** 2025-07-25

**Authors:** Silvia Giagio, Paolo Emilio Adami, Stéphane Bermon, Tamara Rial-Rebullido, Paolo Pillastrini, Marco Vecchiato, Frederic Garrandes

**Affiliations:** 1 Department of Biomedical and Neuromotor Sciences (DIBINEM), Alma Mater Studiorum University of Bologna, Bologna, Italy; 2 Division of Occupational Medicine, IRCCS Azienda Ospedaliero-Universitaria di Bologna Policlinico di Sant’Orsola, Bologna, Italy; 3 Health and Science Department, World Athletics, Monaco; 4 Physiology and Pharmacology Department “V. Erspamer”, University of Rome La Sapienza, Rome, Italy; 5 Laboratoire Motricité Humaine Expertise Sport Santé (LAMHESS), Université Côte d’Azur, Nice, France; 6 Department of Health and Physical Education, Monmouth University, West Long Branch, New Jersey, USA; 7 Sports and Exercise Medicine Division, Department of Medicine, University of Padova, Padova, Italy

**Keywords:** Athlete, Pelvic floor, Sport, Athletics

## Abstract

**Objectives:**

Pelvic floor dysfunction (PFD) symptoms are common among athletes but remain underexplored in youth of both sexes competing in track and field. The primary objective was to assess the prevalence of PFD. Secondary objectives evaluated symptoms impact, awareness of pelvic floor health, related behaviours and gynaecological health in females.

**Methods:**

This observational, cross-sectional study was conducted during the Lima 2024 World Athletics U20 Championships. All athletes were eligible and invited to complete a multilingual, anonymous web-based survey assessing pelvic floor health.

**Results:**

Of the 325 athletes who participated (59.1% females, 40.9% males), 43.7% (n=142) reported PFD symptoms. Prevalence was similar across daily life and athletics activities. Overactive bladder and pelvic pain were the most common conditions. Females had higher PFD rates (n=103, 53.7%) compared with males (n=39, 29.3%). Athletes with athletics-related urinary incontinence (n=42; 12.9%) reported frustration and reduced concentration during performance. Symptomatic athletes had low body mass index and reported more maladaptive pelvic floor-related behaviours than asymptomatic athletes (p<0.05). Menstrual issues and contraceptive use were more frequent among females with PFD (p<0.05). Fewer than 30% (n=95) were aware of pelvic floor health, and 88% (n=286) had never undergone screening. Most did not disclose symptoms (n=111; 78.2%) or seek specialised care (n=135; 95.1%).

**Conclusions:**

PFD was prevalent among elite youth athletes, particularly females. These findings highlight the need for proactive strategies, including education, embedding pelvic floor health in medical evaluations and addressing sex-specific needs to optimise athletes’ health throughout their careers.

**WHAT IS ALREADY KNOWN ON THIS TOPIC**

- Pelvic floor (PF) dysfunction is a recognised issue in athletes, with most research focusing on adult female populations and urinary incontinence.
- Comprehensive PF health among elite youth athletes of both sexes is unexplored.

**WHAT THIS STUDY ADDS**

- This is the first study to in-depth assess PF health in both male and female elite youth athletes competing in athletics.
- Nearly half of the participants reported at least one PF dysfunction symptom, with overactive bladder and pelvic pain being the most prevalent.
- Differences between symptomatic and asymptomatic athletes were observed in relation to low body mass index, maladaptive PF-related behaviours and female-specific characteristics including menstrual health.

**HOW THIS STUDY MIGHT AFFECT RESEARCH, PRACTICE OR POLICY**

- There is a need to integrate PF health screening into routine medical evaluations for youth athletes to ensure ongoing monitoring, early identification and symptom management.
- Raising awareness through education and structured proactive interventions can help reduce stigma and improve athlete care by fostering open discussions on PF health.
- Findings underscore the need for multidisciplinary collaboration in sports medicine.

## Introduction

 Pelvic floor dysfunction (PFD) encompasses a wide range of symptoms, including urinary (UI) and anal incontinence, pelvic organ prolapse, overactive bladder syndrome and pelvic pain.^1^ According to literature, both male and female athletes engaging in diverse sports may experience various forms of PFD.^2^ Within its multifactorial pathophysiology,^1 3^ high-intensity and impact sports, such as athletics, may further challenge the tolerance and functionality of pelvic floor (PF) muscles, contributing to the development or worsening of PFD symptoms.^4^,^7^

Despite growing recognition of the importance of PF health in sports medicine,^8 9^ substantial gaps persist in the current evidence base. Most available studies focused on adult female athletes, primarily investigating UI across various sports.^2^ In this group, the risk of UI is nearly three times higher than in the general population,^10^ with prevalence rates reaching up to 80% in gymnastics.^6^ To date, only a few studies specifically targeted athletes participating in multiple athletics disciplines.^411^,^14^ Research in youth populations also remains limited. Only one systematic review assessed UI among adolescent females, reporting an average prevalence of 48.6% across different sports.^15^ However, no primary studies have comprehensively evaluated a range of PFDs, beyond UI, among elite youth athletes of both sexes, particularly in the context of athletics. This under-representation contributes to a critical knowledge gap regarding PF health in this population.

The primary objective of this study was to determine PFD symptoms prevalence among elite youth athletes of both sexes. Further objectives were to explore: (a) symptoms impact on emotional well-being and athletic performance; (b) awareness of PF health; (c) PF-related behaviours and (d) gynaecological health in females. In addition, we analysed differences in characteristics between athletes with and without PFD symptoms. This study may provide valuable insights into the unique PF challenges faced by youth elite athletes and underscore the role sports international federations can play in promoting health initiatives.

## Methods

### Study design

This was an observational, cross-sectional study conducted through a multilingual web-based survey at the Lima World Athletics Under 20 Championships (27 August 2024–31 August 2024). All data were anonymised and stored on a general data protection regulation-compliant online system. Reporting followed the Strengthening the Reporting of Observational Studies in Epidemiology and the Checklist for Reporting Results of Internet E-Surveys guidelines. World Athletics supported the research. The study protocol was prospectively registered and is available elsewhere.^16^

### Equity, diversity and inclusion

The research team comprised women and men with diverse professional backgrounds and geographical representation. The study included all gender categories from World Athletics’ member Federations, ensuring ethnic and cultural diversity. We focused on youth athletes, an underrepresented population in this field.

### Patient and public involvement

No involvement.

### Participants

All competing under 20 athletes were eligible to participate voluntarily. Under 20 athletes are defined as individuals who are 19 years old or younger as of 31 December in the year of the competition.

The required sample size was estimated using the formula for proportions with 95% confidence level, p=0.5 and 5% margin of error. Given the finite population of 1720 eligible athletes, a finite population correction was applied, yielding a minimum required sample of approximately 315 participants.

### Survey

The survey was developed by the research team considering athletes’ age, cultural backgrounds and medical literacy, with key definitions provided in the survey introduction. Initial pilot testing was conducted internally to assess feasibility, usability, clarity and engagement. Subsequently, external professionals reviewed multilingual versions (English, Italian, Spanish and French) to ensure linguistic accuracy, cultural appropriateness and suitability for a youth international population. Minor adjustments were made accordingly. Once finalised, a custom QR code was generated to link directly to the web-based platform (SurveyMonkey, San Mateo, California, USA, it.surveymonkey.com). The survey was closed access and anonymous. Duplicate entries were checked by IP address. The final version covered: (1) demographics and anthropometrics; (2) medical history; (3) sports-related characteristics and (4) PF health. The PF health section comprised assessment of self-reported PFD symptoms, including UI, anal incontinence, pelvic organ prolapse, overactive bladder and pelvic pain. Questions were extracted from part A of the PFD-SENTINEL tool^17^ and adapted for youth athletes and online administration, including a change in phrasing from “Do you usually experience…” to “Have you ever…”. Table 1 summarises the clinical terminology for PFD and the corresponding simplified wording used in the survey. UI was further investigated using the International Consultation on Incontinence Questionnaire-UI Short Form (ICIQ-UI-SF).^1^ Only athletes who reported leaking exclusively during activities related to athletics, including strength and conditioning sessions, were categorised as having athletics-related UI. Only those who reported having athletics-related UI were asked what specific activities caused leakage. For all athletes, additional PF health questions included symptom impact, awareness, behaviours, prior experience with questionnaire-based screening for PF, management strategies and medical evaluations or treatments. Female athletes answered additional gynaecological health questions (see online supplemental file 1).

**Table 1:** Pelvic floor dysfunction (PFD) symptoms and corresponding wording used in the survey

PFD symptom, terminology	PFD question, wording used in the survey*
Urinary incontinence	Have you ever experienced involuntary loss of urine?
Overactive bladder syndrome	Have you ever experienced urinary urgency (that is a strong sensation of needing to go to the toilet) usually accompanied by frequent urination and nocturia?
Pelvic organ prolapse (females)	Have you ever observed or felt a bulge at or protruding from the vaginal opening?
Anal incontinence	Have you ever experienced involuntary loss of stool or gas?
Pelvic pain	Have you ever experienced pain or discomfort in the lower abdomen or genital region?

* The table provides internationally recognised clinical terminology and the simplified wording used in the survey. Questions were adapted from part A of the PFD-SENTINEL tool after piloting for suitability in a youth athletic population. After a brief introductory section explaining common PFD symptoms, athletes were asked whether they had experienced any of the following.

### Data collection

In Lima (Peru), athletes were invited to scan the QR code at the Health & Science Department booth in the athletes’ zone, linking to the survey. Data were collected exclusively during the days of competitions. World Athletics’ medical staff (PEA, FG and SG) provided support on site. On completion, athletes could download an educational leaflet on PF health and receive elastic training bands as a token of gratitude.

### Statistical analysis

Only fully completed surveys were analysed. No duplicates were recorded. The target sample size was reached, and no imputation of missing data was performed. Categorical variables were summarised as frequencies (n) and percentages (%). Standardised prevalence rates were calculated across continents and disciplines. Continuous variables were presented as means and SD or medians, ranges (minimum–maximum), and IQR, depending on data distribution. Normality was assessed through the Shapiro-Wilk test.

Events were classified based on the Athletics’ event participation and on previous Athletics studies.^18 19^ Participants were categorised as ‘symptomatic’ if they reported ever experiencing at least one PFD symptom, whether in daily life or during Athletics-related activities. Otherwise, they were categorised as ‘asymptomatic’.

Analyses exploring differences between symptomatic and asymptomatic athletes were conducted separately for male and female subgroups, as well as for the overall combined sample (males and females). χ² tests were used for categorical variables. For continuous variables that did not meet normality assumptions (body mass index (BMI), daily training volume, weekly training frequency and age at menarche), the Mann-Whitney U test was applied. All variables listed in online supplemental file 2 were tested. All tests were two-sided, and a p<0.05 was considered statistically significant. Analyses were performed using SPSS V.24 (SPSS).

## Results

### Study population

Among the 1720 athletes who competed in Lima, the study achieved a participation rate of 18.9% and a completion rate of 78.1% (325 of 416). The targeted sample size was reached.

In total, 325 elite athletes (n=192; age 18.1±1 years; 59.1% females; n=133, 40.9% males) from 62 countries across the six continents participated in the study. Table 2 summarises participants’ key demographic, anthropometric, medical history and sports-related characteristics. Additional analyses are provided in onlinesupplemental files 3AF and 4A.

**Table 2:** Main demographic, anthropometric, medical history and sports-related characteristics

Variable*	Total n=325	Females n=192	Males n=133
Sex	–	192 (59.1)	133 (40.9)
Age	18.1±1	18±1	18.3±1
Weight (kg)	64.7±14	59.6±10.7	72.2±14.8
Height (cm)	173.5±9.6	169.1±7.7	179.8±8.9
BMI (kg/m^2^)	21.4±3.3	20.8±3.0	22.2±3.6
Place of birth, continent			
Europe	154 (47.4)	100 (52.1)	54 (40.6)
South America	65 (20)	36 (18.8)	29 (21.8)
Asia	42 (12.9)	25 (13)	17 (12.8)
North America	40 (12.3)	20 (10.4)	20 (15)
Africa	18 (5.5)	7 (3.6)	11 (8.27)
Oceania	6 (1.8)	4 (2.1)	2 (1.5)
Regular medication intake	8 (2.5)	4 (2.1)	4 (3)
Muscle or bone injuries in lower belly or pelvic area	37 (11.4)	19 (9.9)	18 (13.5)
Number of stress fractures during the career			
None	256 (78.8)	152 (79.2)	104 (78.2)
One to three	60 (18.5)	34 (17.7)	26 (19.5)
More than three	9 (2.8)	6 (3.1)	3 (2.3)
Sports-related characteristics			
Training hours/day	2.8±1.2	2.9±1.2	2.7±1.1
Training sessions/week	6±2	6.2±2	5.7±1.9
Competitions/month	2.6±1.8	2.7±2	2.4±1.5
Years as competitive athlete	4.9±2.5	5±2.5	4.7±2.5
Participation in other sports or training activities	97 (29.8)	63 (32.8)	34 (25.6)
Athletics disciplines			
Sprints	128 (39.4)	67 (34.9)	61 (45.9)
Hurdles	39 (12)	25 (13)	14 (10.5)
Middle-distance runs	39 (12)	26 (13.5)	13 (9.8)
Jumps	38 (11.7)	28 (14.6)	10 (7.5)
Throws	29 (8.9)	17 (8.8)	12 (9)
Race walking	26 (8)	12 (6.2)	14 (10.5)
Long-distance runs	19 (5.8)	11 (5.7)	8 (6)
Combined events	7 (2.1)	6 (3.1)	1 (0.7)

Results are reported as n (%) and mean±SD.

Muscle or bone injuries in lower belly or pelvic area=this definition includes groin pain, hamstring muscle injury, testicular pain, hip fracture, ‘psoas pain’, as reported by athletes.

Number of training sessions/week=this includes additional training sessions, such as gym workouts or other sports activities, in addition to athletics.

Participation in other sports or training activities=refers to sports or training engagements outside of athletics.

Further details are provided in online supplemental files.

Descriptive analysis for the total number of athletes (n=325), reported by sex.

* Sports-related characteristics=metrics include the average number of hours, sessions and competitions.

BMI, body mass index.

### PFD symptoms: overview of key findings

**Figure 1:**
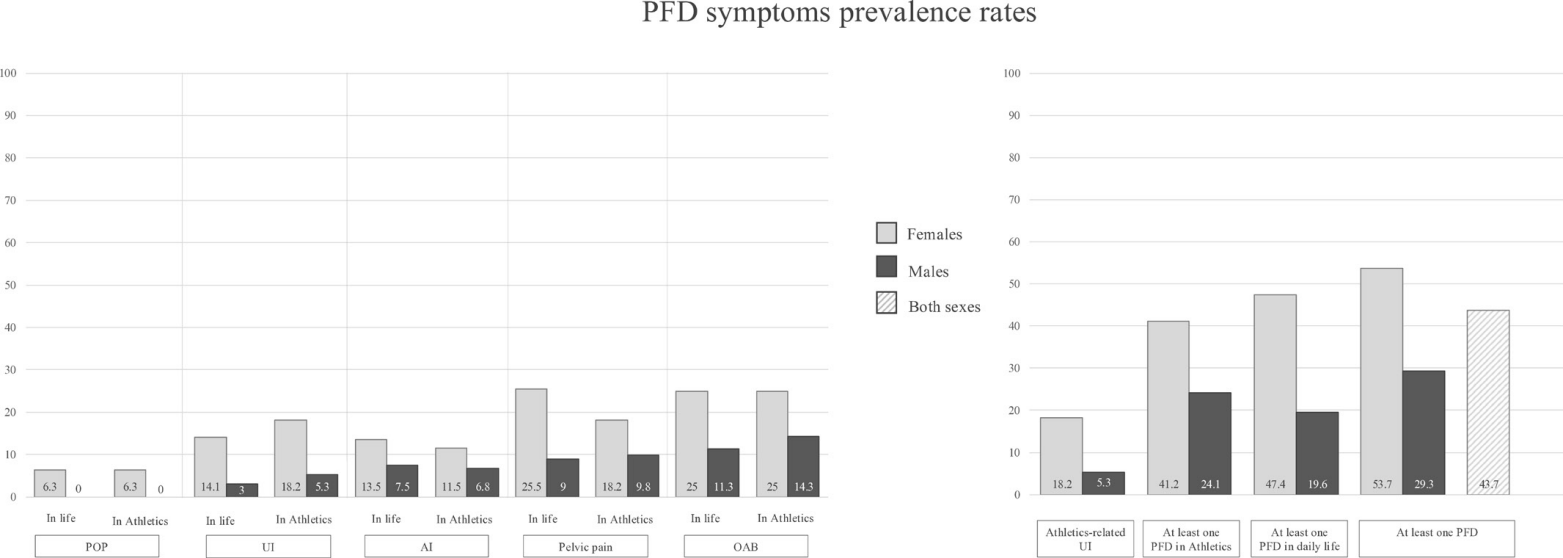
PFD prevalence rates, in female (n=192) and male (n=133) athletes. AI, Anal Incontinence; OAB, Overactive Baldder; PFD, Pelvic Floor Dysfunction; POP, Pelvic Organ Prolapse; UI, Urinary Incontinence.

Nearly half of athletes (n=142; 43.7%) reported at least one PFD symptom. Symptoms of overactive bladder were the most common, reported by 19.4% (n=63) of athletes in daily life and 20.6% (n=67) during Athletics. Pelvic pain prevalences were 18.8% (n=61) in daily life and 14.8% (n=48) during sport. Anal incontinence rates were 11.1% (n=36) and 9.5% (n=31), respectively. Symptoms suggestive of pelvic organ prolapse were less frequent (n=12, 6.3%, in females). Figure 1A–C displays prevalence rates by sex, comparing symptoms in daily life and athletics, along with aggregated rates for the entire cohort. In particular, athletics-related UI was reported by 12.9% (n=42) of athletes across both sexes, mostly triggered by athletic activities such as jumping, sprinting and change of direction.

According to the ICIQ-UI-SF questionnaire, 66.7% (n=21) of athletes reported leaking small amounts of urine, primarily during general physical activity or exercise, with an average impact rating of 3.6 (SD=2.62) on a 10-point scale. The mean ICIQ-UI-SF total score for the overall sample (n=31) was 8.2 (SD=3.8), with similar values observed in females (8±4) and higher scores in males (10±1.4). Regarding validated severity categories, 38.7% (n=12) of athletes were classified as having slight symptoms, 45.2% (n=14) as moderate, and 16.1% (n=5) as severe. Among females, 44.4% (n=12) reported slight symptoms, 37% (n=10) moderate and 18.5% (n=5) severe. Among males, all participants (n=4; 100%) were classified in the moderate category. No athletes of either sex reported symptoms in the ‘very severe’ range.

Table 3 summarises the comparative analysis of symptomatic versus asymptomatic athletes, reporting the variables that differed significantly between groups. The prevalence of PFD symptoms differed by sex, with over 70% of symptomatic athletes being female (p=0.001). Among both female and male athletes with athletics-related UI, median BMI was lower in the symptomatic group than in the asymptomatic group. Pelvic injuries were significantly more common among symptomatic females (p<0.005), while stress fractures were more frequently reported by symptomatic males (p=0.015). Online supplemental file 5 shows an example of the statistical report, reporting differences between symptomatic and asymptomatic athletes in the combined sample of males and females.

**Table 3:** Variables showing statistically significant differences between symptomatic and asymptomatic athletes: combined sample of males and females (n=325), females (n=192) and males (n=133)

Variable*		Symptomatic females and males			Symptomatic females			Symptomatic males		
		PFD in life	PFD in Athletics	Athletics- UI	PFD in life	PFD in Athletics	Athletics- UI	PFD in life	PFD in Athletics	Athletics- UI
General characteristics	Sex	X	X	X						
	BMI (kg/m^2^)*		X	X			X		X	X
Medical history	Respiratory and breathing issues	X			X					
	Pelvic injury				X	X				
	History of one to three stress fractures								X	
PF domain	PF awareness	X	X	X	X		X			
	PFD awareness	X	X	X	X		X			
	Going to the toilet frequently during training	X	X	X				X	X	
	Need to push or strain during bowel movement in daily life	X	X		X	X				
	Caffeine gels/drinks or energy supplements consumption	X	X		X					
	Difficulty starting urination in daily life		X		X					
	Reducing liquid intake			X			X			
	Going to the toilet *before* training or competing	X								
	Going to the toilet frequently during event or competition		X							
Sports- related characteristics	Long distance runs	X								
	Middle distance runs			X						
	Throws		X							
	Participation in other sports or trainings	X								
	Training (hours/day)*						X			
Female health	Change in menstrual cycle				X	X				
	Hormonal medication or other contraceptive methods				X		X			

In the present table, only statistically significant differences between symptomatic and asymptomatic athletes (p<0.05) are reported.

For example, among symptomatic females and males in daily life, respiratory and breathing issues were more prevalent compared with asymptomatic athletes (p<0.05).

Change in menstrual cycle=Change in menstrual cycle when increase of exercise intensity, frequency or duration.

Pelvic injury=Muscle or bone injuries in lower belly or pelvic area.

PF awareness=PF anatomy and function awareness.

Unless otherwise specified, the χ2 test was used for categorical variables.

* The Mann-Whitney U test was used to compare symptomatic and asymptomatic athletes for continuous variables with non-normal distributions.

BMI, body mass index; PF, pelvic floor; PFD, pelvic floor dysfunction; UI, urinary incontinence.

### PFD prevalence rates across continents and Athletics disciplines

Across continents, the highest PFD prevalence was in Oceania (n=4; 66.7%), followed by Asia (n=20; 47.6%), Europe (n=70; 45.5%), Africa (n=8; 44.4%), South America (n=27; 41.5%) and North America (n=14; 35%).

**Figure 2:**
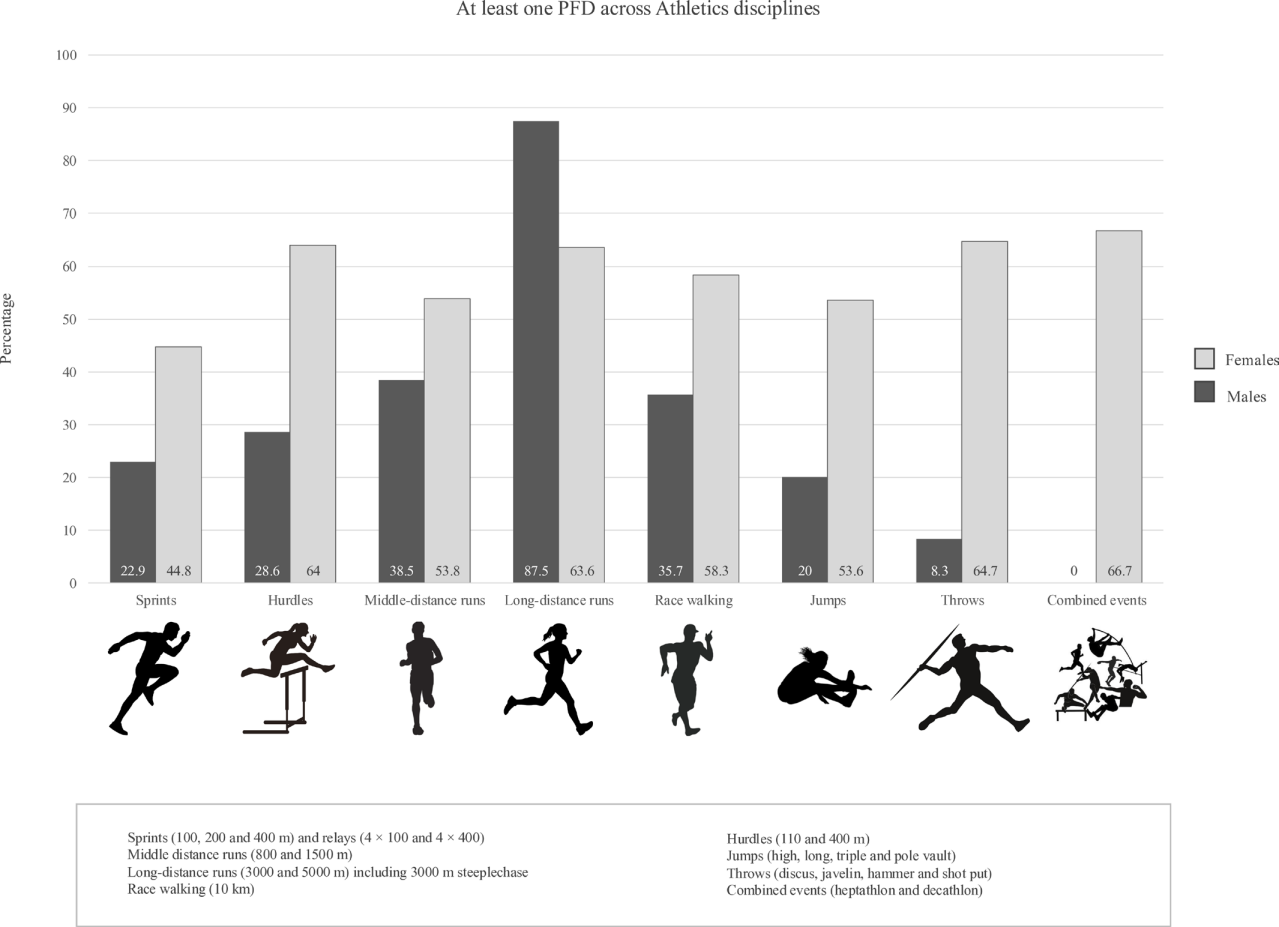
Prevalence rates of at least one PFD in female and male athletes (n=325), according to specific athletic disciplines. PFD, Pelvic Floor Dysfunction.

Long-distance runners exhibited the highest PFD prevalence (73.7%), followed by athletes in combined events (57.1%). Figure 2 provides a descriptive analysis stratified by sex. Symptoms in daily life were more frequent in long-distance runners (p=0.002) and in athletes engaged in additional sports or training activities (p=0.014), compared with asymptomatic peers. Athletics-related UI was more commonly reported by athletes participating in middle distance runs (p=0.012). However, these differences were no longer statistically significant when analyses were stratified by sex (table 3).

### Impact of PFD symptoms and management strategies

**Figure 3:**
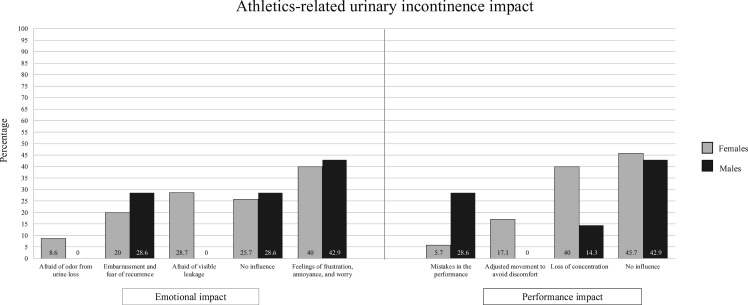
Emotional and performance impact of athletics-related urinary incontinence in female and male athletes.

Figure 3 illustrates the impact of athletics-related UI on emotional well-being and athletic performance.

Of the 142 athletes with any PFD, 78.2% (n=111) never disclosed their condition. Among those who did, most (n=15; 48.4%) turned to healthcare professionals or family members (n=15; 48.4%), rather than coaches (n=10; 32.3%). A total of 83.8% (n=119) did not adopt any mitigation strategies for symptoms. The use of pads was reported by 13.6% of females (n=14) and 10.3% of males (n=4). Only 4.9% (n=7) underwent specialised assessments and 14.1% (n=20) declared engaging in PF intervention. One female athlete had experience in PF exercises.

### Awareness of PF health and related behaviours

Table 4 presents the main information regarding PF health among all athletes. Only 30% (n=95) reported being aware of PF health. Significant differences in terms of awareness were found between symptomatic and asymptomatic athletes of both sexes (p<0.05), as shown in table 3 alongside other relevant variables. Most athletes (n=286; 88%) had never undergone questionnaire-based PF medical screening during routine medical evaluations. 35.7% of the 116 athletes who reported having access to a health professional within their team to discuss PF health, sports physicians were the most cited (n=61; 52.6%), followed by sports physiotherapists (n=44; 37.9%).

**Table 4:** PF health domain

Variable	Total n=325	Females n=192	Males n=133
PF awareness	95 (29.2)	65 (33.8)	30 (22.6)
PFD awareness	69 (21.2)	46 (24)	23 (17.3)
PF-related behaviours			
Difficulty starting urination in daily life	27 (8.3)	12 (6.3)	15 (11.3)
Need to push or strain during bowel movement in daily life	37 (11.4)	28 (14.6)	9 (6.8)
Going to the toilet before training or competing	236 (72.6)	154 (80.2)	82 (61.6)
Reducing liquid intake during training or competing	84 (25.8)	52 (27.1)	32 (24.1)
Going to the toilet frequently during training	117 (36)	79 (41.2)	38 (28.6)
Going to the toilet frequently during event or competition	132 (40.6)	88 (45.8)	44 (33.1)
Consumption of gels/drinks or energy supplements with caffeine	108 (33.2)	62 (32.3)	46 (34.6)

Results are reported as n (%).

Descriptive analysis for the total number of athletes (n=325), reported by sex.

PF awareness, PF anatomy and function awareness.

PF, pelvic floor; PFD, pelvic floor dysfunction.

Maladaptive PF-related behaviours, such as straining during bowel movements, frequent urination before or during training and difficulty initiating urination in daily life, were significantly more prevalent among females with PFD compared with males (p<0.005). In addition, liquid restriction was more frequently reported by females with athletics-related UI than by their asymptomatic peers (p=0.006).

### Gynaecological health in female athletes

Among female athletes, 59.9% (n=115) had never undergone a gynaecological visit, and 74% (n=142) did not attend regular check-ups. The mean age of menarche was 13.5 years (SD=1.7). One 17-year-old athlete reported no history of menarche, consistent with the clinical definition of primary amenorrhoea. Menstrual patterns that changed due to increased exercise intensity, frequency or duration were reported by 39.6% (n=76), with differences between symptomatic and asymptomatic athletes (54.2% vs 31.3%; p=0.002). 31.3% reported irregular menstrual cycles (n=60). Hormonal contraceptive users (n=31; 16.2%) had higher PFD prevalence during life (p=0.037) and UI in athletics compared with the asymptomatic group (p=0.007).

## Discussion

This study represents the first comprehensive overview of PF health among elite youth athletes of both sexes participating in athletics. Our findings revealed the presence of PFD symptoms among younger athletes, with similarities and differences compared with adult athletes.

### Need for education and screening

Athletes reported limited prior knowledge of PF health, consistent with previous studies in adolescent and adult athletes across various sports.^11 12 15 20^ Athletes with greater awareness also reported experiencing more PFDs, suggesting that the presence of symptoms may heighten awareness or, conversely, that increased awareness facilitates symptom recognition. Only 10% of athletes reported undergoing questionnaire-based medical screening for PF, confirming the gap in routine PF health screening in sports medicine practices.^21^

### Need for specialised referrals in youth athletes

Nearly half of the elite athletes reported at least one PFD symptom, suggesting the need for timely specialist referrals. Our findings align with recent Athletics research reporting PFD prevalence close to 50%,^4 11^ particularly among females.^2 4^ Overactive bladder symptoms were the most frequently reported, in contrast with the SUI patterns typically observed in adult athletes.^6^ A broader range of PFDs was also identified, including anal incontinence and pelvic pain. Symptoms suggestive of pelvic organ prolapse were infrequent, likely due to the absence of major risk factors.^3^ Unlike adults, who frequently reported symptoms during sports activities,^22^ our cohort showed similar rates of occurrence in both daily life and athletics.

Differences between symptomatic and asymptomatic athletes were more related to general factors (eg, low BMI, sex specific characteristics, PF-related behaviours) than to sport-specific variables. Although low BMI findings contrast with general population trends,^23^ our results align with studies on UI in female athletes, suggesting it may have a role in PFD.^24^ The history of stress fractures and pelvic injuries emerged as previously unexplored contributors to PFD.

### A worldwide challenge in all athletics disciplines

While a recent study on adult Spanish athletes suggested higher UI prevalence in specific disciplines,^4^ we found no substantial differences in PFD prevalence across continents or disciplines when analysed by sex. Comparisons with previous research are challenging due to heterogenous event classifications and our broader focus on overall PF health. Based on the available evidence, a direct relationship between specific physical demands in athletics and PFDs is still inconsistent. Symptoms are likely influenced by a combination of individual and sports-related characteristics endured by elite youth athletes, regardless of discipline. Further investigations within this sample are warranted to ensure a more comprehensive interpretation.

### From emotional impact to barriers in seeking help for PFD

In line with previous literature,^22 24^ athletes reported frustration, annoyance, worry and a loss of concentration due to symptoms. These factors may undermine sport enjoyment, motivation and long-term engagement. Despite this burden, symptoms were rarely disclosed or discussed,^15^ possibly due to stigma, social normalisation of PFD or limited perceived impact.^12 25^ As a result, athletes did not seek medical advice and/or undergo specialised assessment and instead relied on improvised strategies to mitigate symptoms.^5 22 23^ These included sanitary product use and altering bladder emptying behaviours or fluid intake while training. As previously noted,^5^ these maladaptive behaviours may contribute to the persistence, progression or worsening of PFD symptoms. While 14.1% of athletes reported engaging in therapeutic PFD interventions, only a small proportion of these underwent specialised assessments. It may be possible that some athletes relied on self-implemented strategies, which merit cautious consideration.

### Sex-specific insights for PF health

The study identified sex-specific differences which highlight the importance of tailored approaches.^2 5 14^ Consistent with previous literature, PFD prevalence was lower among males,^14 26^ while nearly half of the female athletes reported at least one symptom. Our findings reinforce current evidence^27^ and align with the International Olympic Committee (IOC) recommendations to monitor female-specific health domains.^8^ Our data also revealed that low rates of gynaecological health visits, despite the high prevalence of menstrual disorders, reflect a lack of awareness, as previously demonstrated in other youth health domains.^18^ The American College of Obstetricians and Gynecologists recommends that the initial reproductive health visit occur between 13 and 15 years of age.^28^ Addressing the PF health needs of female athletes requires a broader perspective that extends beyond the scope of sports medicine to consider reproductive, psychological and social determinants of health.

In addition, the relationship between low BMI, menstrual irregularities, bone stress fractures and PFDs, particularly UI, warrants careful consideration as these factors may be components of complex and multifactorial clinical presentations, such as Relative Energy Deficiency in Sport.^29^ While awaiting further research, clinicians should remain vigilant during screening and evaluation.

### Clinical implications

A proactive approach based on education, monitoring and defined referral pathways by medical staff is warranted. This population does not present common PFD risk factors such as parity or advanced age,^3^ yet they may face additional pelvic health challenges throughout their career and lifespan.^3 7^ Monitoring may help identify predisposing or intervening factors that could contribute to the development or exacerbation of PFD, potentially influencing PF load tolerance.^3 7^ For symptomatic athletes, timely recognition may prompt appropriate referral and intervention as well as mitigate long-term consequences.^30^ Moreover, increasing athletes’ and stakeholders’ awareness can help break the barriers, stigma and misconceptions surrounding PF health.

### Proactive approach: a priority for World Athletics

As part of its commitment to integrating PF health into sports medicine, World Athletics has undertaken several initiatives within this project, including the distribution of educational materials, social media dissemination and dedicated workshops. The next phase will assess medical staff knowledge and practices to support a proactive, athlete-centred approach. This requires collaboration across Federations, clinicians and coaches, with attention to cultural and population-specific needs.

### Strengths and limitations 

This study provides novel insights into PF health in an international sample of elite youth athletes, ensuring diverse geographical and cultural representation. The survey’s availability in four languages enhanced accessibility and inclusivity. By assessing a broad range of PFDs and PF health domains, our study expands knowledge and aligns with IOC recommendations on female athlete health and global research priorities. A conservative approach was adopted by including only fully completed surveys with no imputation of missing data, in order to minimise unreliable, partial or inconsistent responses.

However, findings should be interpreted with caution. Although the target sample size was reached, participation rates and limited representation from certain continents may affect generalisability. We used the PFD-SENTINEL as it is currently the only available tool for athletic populations, although it is undergoing validation.^31^ Self-reported data may have introduced biases, particularly in the accurate identification of PFD and in distinguishing symptoms (eg, pelvic pain) from other conditions (eg, cramping or groin pain). To mitigate this, basic information was included in the questionnaire, and researchers were available to assist participants. The item used to assess pelvic organ prolapse captures only more advanced cases, the prevalence and its interpretation must be contextualised accordingly. Finally, the cross-sectional design prevents causal inferences, and larger sample sizes are needed to explore emerging factors such as stress fractures and pelvic injuries.

## Conclusions

While PFD symptoms were prevalent among youth athletes, awareness of PF health and access to routine screenings remained limited. Nearly half of the participants reported any symptoms with higher prevalence rates of overactive bladder symptoms and pelvic pain. Although low rates of Athletics-related UI were reported, it was frequently associated with feelings of frustration, annoyance, worry and a loss of concentration during training or competition. Comparisons between symptomatic and asymptomatic athletes revealed differences in low BMI and PF-related maladaptive behaviours in both sexes. Among females, sex-specific factors including menstrual health were more frequently reported by those with PFD. These findings support the need for a proactive approach to PF health in youth athletes. This includes integrating screening into routine evaluations to ensure ongoing monitoring, early identification, timely referral and appropriate management. Promoting awareness through education may further reduce stigma and enhance care within multidisciplinary teams.

## Supplementary material

10.1136/bmjsem-2025-002564 online supplemental file 1

10.1136/bmjsem-2025-002564 online supplemental file 2

10.1136/bmjsem-2025-002564 online supplemental file 3

10.1136/bmjsem-2025-002564 online supplemental file 4

10.1136/bmjsem-2025-002564 online supplemental file 5

## Data Availability

Data are available on reasonable request.
