# Supplementary material for: Nearly half of 325 athletes reported pelvic floor symptoms: a cross-sectional study at the Lima 2024 World Athletics U20 Championships
Source: BMJ Open Sport Exerc Med. 2025 Jul 25;11(3):e002564. doi: 10.1136/bmjsem-2025-002564 (PMC12306240; doi:10.1136/bmjsem-2025-002564)
Supplement: online supplemental file 1 [file bmjsem-11-3-s001.pdf]

Pelvic Floor Health in Athletics

WHY SHOULD WE CARE ABOUT PELVIC FLOOR HEALTH?

The pelvic floor is essential for everyone, at every stage of life, and for both men and women.
For you, as an athlete, maintaining a healthy pelvic floor is important not only for achieving best performance today but also for ensuring long-term physical well-being and preventing future issues.

WHAT IS THE PELVIC FLOOR?

It is a group of muscles at the bottom of your pelvis. These muscles support important organs like the bladder, intestines, and in women, the uterus.

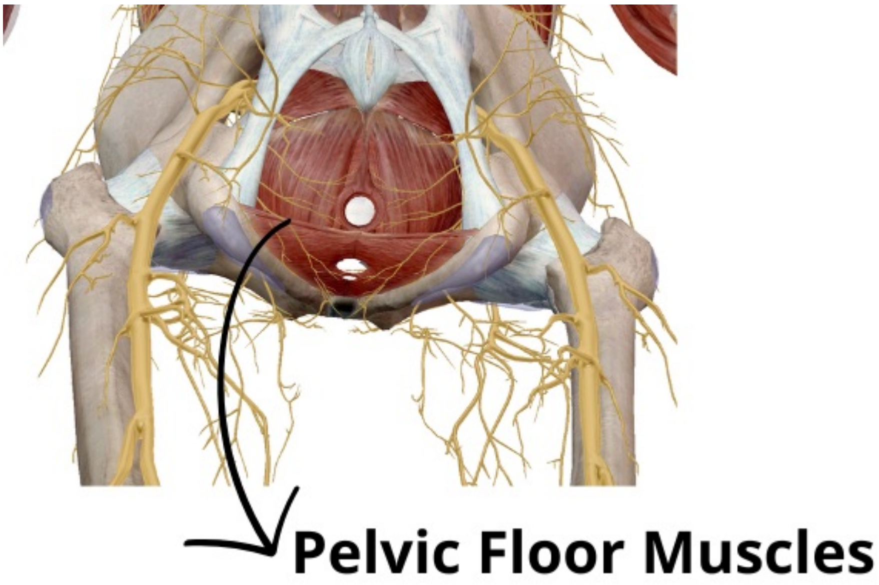

They help with several essential functions:

- Holding organs in place,
- Controlling bladder and bowel movements and prevent leakage,
- Playing a role in sexual health,
- Maintaining balance and strength.

Sometimes, these muscles might not function properly, which can cause discomfort or other symptoms, particularly in athletes. During this survey, we will dive deeper into what this could mean for you.

READY TO START?

We will ask some questions about your pelvic floor health.

Pelvic Floor Health in Athletics

YOUR INFORMATION

Your information are important as they help us analyse the data more accurately and assess how pelvic health issues may vary across different groups.

* 3. How old are you? (years)

[ ]

* 4. What is your **sex**?

- [ ] Female
- [ ] Male
- [ ] Prefer not to say

* 5. What is your **weight**? (kg)

[ ]

* 6. What is your **height**? (cm)

[ ]

* 7. Place of **birth**

[ ]

* 8. What **federation** do you represent in this World Championships?

[ ]

Pelvic Floor Health in Athletics

YOUR MEDICAL HISTORY

Here we gather information about your general health. Understanding your medical history helps us explore possible links between general health factors and pelvic health.

* 9. Do you take any regular **medications**?

- [ ] No
- [ ] Yes (please specify)

____

* 10. Do you **smoke**?

- [ ] No
- [ ] Yes

* 11. Do you have any of the following **health conditions**?

- [ ] Frequent urinary infections (>2 times per year, diagnosed with urine tests)
- [ ] Respiratory and breathing issues like asthma
- [ ] Constipation: it refers to having infrequent bowel movements or trouble passing stool. You might feel like you haven't fully emptied your bowels
- [ ] History of pelvic surgery
- [ ] Other health conditions (please specify)

____

- [ ] None of the above

* 12. Have you ever had **muscle or bone injuries** in your **lower belly or pelvic area**? For example, pain near your groin, muscle tears in your belly, fractures near your pubic bone or trauma in the area

☐ No

☐ Yes (please specify the type of injury on your own words)

* 13. How many **stress fractures** have you experienced? A stress fracture is a type of bone break caused by repetitive stress or force, often from overuse in sports such as repeatedly jumping up and down or running long

☐ None

☐ 1 to 3

☐ More than 3

Pelvic Floor Health in Athletics

ATHLETICS-RELATED INFORMATION

Here, we aim to understand more about your athletic activities.

* 14. In which **event** do you compete? You can select more than one event

- [ ] 100 Metres
- [ ] 200 Metres
- [ ] 400 Metres
- [ ] 800 Metres
- [ ] 1500 Metres
- [ ] 5000 Metres
- [ ] 10000 Metres
- [ ] Marathon
- [ ] 3000 Metres Steeplechase
- [ ] 100/110 Metres Hurdles
- [ ] 400 Metres Hurdles
- [ ] Heptathlon
- [ ] Decathlon
- [ ] High jump
- [ ] Pole vault
- [ ] Long jump
- [ ] Triple jump
- [ ] Shot Put
- [ ] Discus Throw
- [ ] Hammer Throw
- [ ] Javelin Throw
- [ ] Race Walk

* 15. How often and **how much do you train**? Please provide a number for each question

Training hours/day	
Number of training session per week, considering also additional training such as gym session or others) (number/week)	
Number of competitions per month (number/month)	
Number of high-level competitions per season (e.g. international) (number/season)	
Number of years as competitive athlete (years)	

* 16. Do you participate in **other sports or training activities**?

- [ ] No, I do not participate in other activities in addition to athletics
- [ ] Yes, I practice gym activities (including power/weightlifting)
- [ ] Yes, I compete in other sports (please specify)

Pelvic Floor Health in Athletics

YOUR PELVIC FLOOR HEALTH

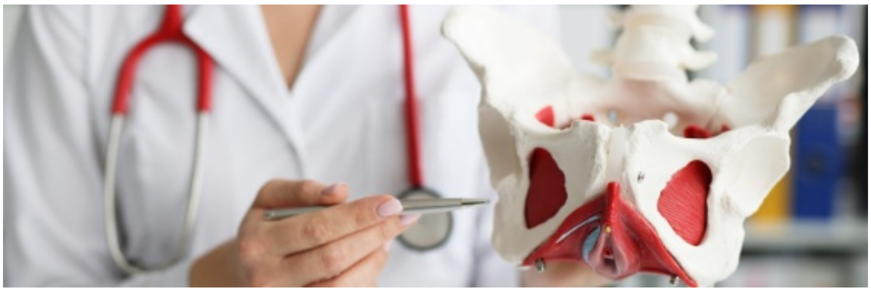

This is the main section. Your experiences and knowledge in this area are invaluable in helping us understand how widespread pelvic floor issues are among athletes.

* 17. Before this survey, were you aware of the **pelvic floor** and its functions?

- [ ] No
- [ ] Yes

* 18. Before this survey, were you aware of **pelvic floor dysfunctions**?

- [ ] No
- [ ] Yes

* 19. Is there a **health professional** in your sports medical team with whom you can discuss **pelvic floor health**?

- [ ] No
- [ ] Yes
- [ ] Do not know

Pelvic Floor Health in Athletics

* 20. If yes, what **kind of professional**?

◯ Uro-gynaecologist

◯ Sports physician

◯ Urologist

◯ Pelvic floor physiotherapist

◯ Sports physiotherapist

◯ Other (please specify)

* 21. Please answer the following **questions**

	No	Yes
In your daily life, do you usually find it hard to start urinating?	◯	◯
In your daily life, do you need to push or strain when you go to the toilet for a bowel movement?	◯	◯
Before training or competing, do you usually go to the toilet?	◯	◯
During training or competing, do you usually reduce liquid intake?	◯	◯
During training, do you often go to the toilet?	◯	◯
During the event or during competition, do you often go to the toilet?	◯	◯
Do you usually intake gels/drinks or energy supplements with caffeine?	◯	◯
Do you use sanitary pads/products during physical activities?	◯	◯

22. In the following section, we included additional specific sex-related questions.

Please confirm your sex here to continue, thank you.

☐ Female

☐ Male

Pelvic Floor Health in Athletics
FEMALE SECTION

23. Age of **menarche** (first menstruation)

[ ]

* 24. Do you usually have a **regular, menstrual cycle** (approximately every 28 days) while training and competing?

- [ ] No, I usually have irregular menstrual cycle
- [ ] Yes
- [ ] Yes, but I was on contraceptives
- [ ] Not applicable (I do not have a menstrual cycle for any reason)

* 25. Does your **menstruation cycle change** when you **increase** your exercise intensity, frequency or duration?

- [ ] No
- [ ] Yes
- [ ] Not applicable (I do not have a menstrual cycle for any reason)

* 26. Does your menstrual cycle **hurt you**?

- [ ] No
- [ ] Yes
- [ ] Not applicable (I do not have menstrual cycle for any reason)

* 27. Have you ever been evaluated by a **gynaecologist**? A gynaecologist is a doctor who specializes in treating the female reproductive system

- [ ] No
- [ ] Yes

* 28. Do you have any **gynaecological problems**?

- [ ] No gynaecological problems
- [ ] Polycystic ovarian syndrome
- [ ] Endometriosis
- [ ] Pelvic inflammatory disease
- [ ] Gynaecological disorders related to menstrual disorders
- [ ] Recurrent vaginal infections
- [ ] Other (please specify)

* 29. Do you use **hormonal medications** or other **contraceptive** methods? These includes hormonal pills, vaginal rings and intrauterine devices

- [ ] No
- [ ] Yes

Pelvic Floor Health in Athletics

* 30. If yes, started to use it to:

- [ ] Avoid pregnancy
- [ ] Regulate menstruation
- [ ] Both reasons

* 31. Have you ever experienced **pain using vaginal tampons?**

- [ ] No, because I do not use vaginal tampons
- [ ] No
- [ ] Yes

* 32. Have you ever observed or felt a **bulge at or protruding from the vaginal opening**?

- [ ] No
- [ ] Yes

* 33. Do you undergo **regular gynaecological check-ups** (at least once a year)?

- [ ] No
- [ ] Yes

Pelvic Floor Health in Athletics

SYMPTOMS OF PELVIC FLOOR DYSFUNCTION

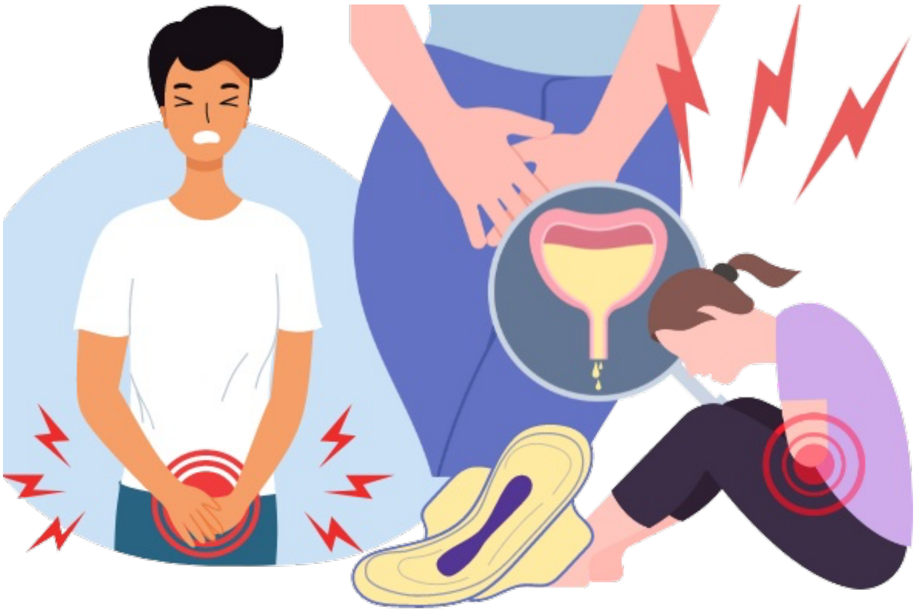

Identifying symptoms of pelvic floor dysfunction is essential for our research.

Pelvic floor dysfunctions occur when the pelvic floor muscles are unable to perform their normal functions effectively. This can lead to a range of symptoms that might affect both **daily life** and **athletic performance**. Key dysfunctions include:

- Urinary leakage, frequent urination, difficulty in starting or stopping urination or a sense of urinary urgency, that is, a strong sensation of needing to go to the toilet
- Loss of stool or gas beyond your control or difficulty in defecation
- Pain or discomfort in the lower abdomen or genital region
- A sensation of heaviness or dragging in the female pelvis.

* 34. **In your life**, have you ever experienced any of the following symptoms?

	No	Yes
Urinary urgency (that is a strong sensation of needing to go to the toilet) usually accompanied by frequent urination and nocturia	◯	◯
Involuntary loss of stool or gas	◯	◯
Pain or discomfort in the lower abdomen or genital region	◯	◯

* 35. **In your life**, have you ever experienced involuntary **loss of urine**?

☐ No

☐ Yes

Pelvic Floor Health in Athletics

* 36. **When** does urine leak? (Please tick all that apply to you)

- [ ] Never – urine does not leak
- [ ] Leaks before you can get to the toilet
- [ ] Leaks when you cough or sneeze
- [ ] Leaks when you are asleep
- [ ] Leaks when you are physically active/exercising
- [ ] Leaks when you have finished urinating and are dressed
- [ ] Leaks for no obvious reason
- [ ] Leaks all the time

* 37. **How often** do you leak urine?

- [ ] Never
- [ ] About once a week or less often
- [ ] Two or three times a week
- [ ] About once a day
- [ ] Several times a day
- [ ] All the time

* 38. We would like to know how much urine you think leaks. **How much urine** do you usually leak (whether you wear protection or not)?

- [ ] None
- [ ] A small amount
- [ ] A moderate amount
- [ ] A large amount

39. Overall, **how much does leaking urine interfere** with your everyday life? Please ring a number between 0 (not at all) and 10 (a great deal)

0 ——————————————— 10 [ ]

40. **How** these symptoms **impact your daily life?**

- [ ] No influence
- [ ] Feel frustrated, annoyed, worried
- [ ] Feel embarrassed, afraid it may happen again
- [ ] Afraid of visible leakage
- [ ] Other (please specify)

[ ]

Pelvic Floor Health in Athletics

* 41. **During athletics training or competitions**, have you ever experienced any of the following symptoms?

	No	Yes
Urinary urgency (that is a strong sensation of needing to go to the toilet) usually accompanied by frequent urination	◯	◯
Involuntary loss of stool or gas	◯	◯
Pain or discomfort in the lower abdomen or genital region	◯	◯

* 42. **During athletics training or competitions**, have you ever experienced involuntary **loss of urine**?

☐ No

☐ Yes

* 43. During athletics training or competitions, **when do you usually leak urine**?

☐ Training

☐ Competitions

☐ Specific athletics- activities (jumping, sprinting, changing direction)

☐ Other sports-activities (for example lifting, gym). Please specify

* 44. How do pelvic floor symptoms affect you **emotionally** during **competitions or training**? (Select all that apply)

- [ ] No emotional influence
- [ ] Feel frustrated, annoyed, worried
- [ ] Feel embarrassed, afraid it may happen again
- [ ] Afraid of visible leakage
- [ ] Afraid that urine loss will smell
- [ ] Other (please specify)

* 45. How do pelvic floor symptoms affect your **athletic performance** during competitions or training? (Select all that apply)

- [ ] No influence on performance
- [ ] Loss of concentration
- [ ] Make mistakes in the performance
- [ ] I move differently
- [ ] Other (please specify)

Pelvic Floor Health in Athletics

* 46. If you experience or experienced any type of these pelvic floor symptoms, did you **discuss the problem** with someone?

- ☐ I never experienced any type of pelvic floor symptoms
- ☐ No
- ☐ Yes

Pelvic Floor Health in Athletics

* 47. If yes, with whom?

- [ ] Healthcare professionals
- [ ] Your family/parents
- [ ] Your coach
- [ ] Teammates
- [ ] Other (please specify)

* 48. Are there any **strategies** you use to mitigate symptoms?

- [ ] No strategies
- [ ] Use pads
- [ ] Restrict fluids
- [ ] Other (please specify)

* 49. Have you ever sought any **specialised assessment**?

- [ ] No
- [ ] Yes

* 50. Have you ever undergone any **therapy/intervention** (e.g. physiotherapy exercises, medications)?

- [ ] No
- [ ] Yes

Pelvic Floor Health in Athletics

END PAGE

Thank you for your contribution.
If you have further questions, you may contact us:

Dr. Silvia Giagio, silviagiagioft@gmail.com
Dr. Paolo Emilio Adami, paoloemilio.adami@worldathletics.org
Dr. Frederic Garrandes, frederic.garrandes@worldathletics.org

PLEASE, CLICK THE "DONE" BUTTON TO CONFIRM YOUR RESPONSES.
