# Supplementary material for: Nearly half of 325 athletes reported pelvic floor symptoms: a cross-sectional study at the Lima 2024 World Athletics U20 Championships
Source: BMJ Open Sport Exerc Med. 2025 Jul 25;11(3):e002564. doi: 10.1136/bmjsem-2025-002564 (PMC12306240; doi:10.1136/bmjsem-2025-002564)
Supplement: online supplemental file 2 [file bmjsem-11-3-s002.docx]

**SUPPLEMENTARY FILE 2**. List of variables. Analyses exploring differences between symptomatic and asymptomatic athletes were conducted separately for male and female subgroups, as well as for the overall combined sample (males and females) for each of the following variables.

| **VARIABLE^a^** | **VARIABLE DESCRIPTION** |
| --- | --- |
| Body Mass Index (BMI) | BMI (kg/m2) |
| Sex |  |
| Smoking |  |
| Events | Events in Lima Championships |
| Medication | Regular medication intake |
| Health conditions |  |
| Pelvic injury | Muscle or bone injuries in lower belly or pelvic area |
| History of stress fractures | Number of stress fractures during the career |
| Training hours/day | Average number of hours per day |
| Training sessions/week | Average number of training sessions per week, considering also additional training such as gym session or others (number/week) |
| Participation in other sports or trainings |  |
| Age of first menstruation | Years |
| Regular menstrual cycle while training and competing |  |
| Change in menstrual cycle | Changing in menstruation cycle when increase of exercise intensity, frequency or duration |
| Painful menstrual cycle |  |
| Hormonal medications or other contraceptive methods |  |
| PF awareness | Pelvic floor anatomy and function awareness. |
| PFD awareness | Pelvic floor dysfunction awareness |
| Difficulty starting urination in daily life |  |
| Need to push or strain during bowel movement in daily life |  |
| Going to the toilet before training or competing |  |
| Reducing liquid intake during training or competing |  |
| Going to the toilet frequently during training |  |
| Going to the toilet frequently during event or competition |  |
| Consumption of gels/drinks or energy supplements with caffeine |  |

^A^ Continuous variables are highlighted in grey, while categorical variables are displayed in white.
