# Supplementary material for: Nearly half of 325 athletes reported pelvic floor symptoms: a cross-sectional study at the Lima 2024 World Athletics U20 Championships
Source: BMJ Open Sport Exerc Med. 2025 Jul 25;11(3):e002564. doi: 10.1136/bmjsem-2025-002564 (PMC12306240; doi:10.1136/bmjsem-2025-002564)
Supplement: online supplemental file 3 [file bmjsem-11-3-s003.docx]

**SUPPLEMENTARY FILE 3, A-F.** Complete descriptive analysis. Results are reported as n (%) and mean ± standard deviation (SD).

1. **ANTHROPOMETRIC, DEMOGRAPHIC AND MEDICAL HISTORY CHARACTERISTICS**

*Anthropometric and demographic characteristics. Results for the total number of athletes (n=325) and reported by sex.*

| **Variable*** | **Total**  **n=325** | **Females**  **n= 192** | **Males**  **n= 133** |
| --- | --- | --- | --- |
| **Sex** | - | 192 (59.1) | 133 (40.9) |
| **Age** | 18.1 ± 1 | 18 ± 1 | 18.3 ± 1 |
| **Weight (kg)** | 64.8 ± 14 | 59,62 ± 10.7 | 72.2 ± 14.8 |
| **Height (cm)** | 173.5 ± 9.6 | 169.1 ± 7.7 | 179.8 ± 8.9 |
| **BMI** | 21.4 ± 3.3 | 20.8 ± 3.0 | 22.2 ± 3.6 |
| **Place of birth, continent** |  |  |  |
| Europe | 154 (47.4) | 100 (52.1) | 54 (40.6) |
| South America | 65 (20) | 36 (18.7) | 29 (21.8) |
| Asia | 42 (12.9) | 25 (13) | 17 (12.8) |
| North America | 40 (12.3) | 20 (10.4) | 20 (15) |
| Africa | 18 (5.5) | 7 (3.6) | 11 (8.3) |
| Oceania | 6 (1.8) | 4 (2.1) | 2 (1.5) |

Results are reported as n (%) and mean ± SD.

* Place of birth, continent = place of birth (countries= 64) classification per geographic continent as follows:

*Europe*= Italy, Poland, Germany, Czech Republic, United Kingdom of Great Britain and Northern Ireland, France, Norway, Spain, Estonia, Ukraine, Latvia, Austria, Slovenia, Finland, Lithuania, Portugal, Croatia, Serbia, Belgium, Hungary, Denmark, Switzerland, Bosnia and Herzegovina, Sweden, Romania.

*Asia*= India, Japan, Sri Lanka, Turkey, Kazakhstan, Republic of Korea, China, Israel, Singapore, United Arab Emirates, Lebanon. Turkey was included in Asia.

*Africa*= Botswana, South ‎Sudan, Nigeria, Morocco, South Africa, Algeria, Sudan, Lesotho.

*North America*= United States of America, Canada, Bahamas, Cuba, Mexico, Jamaica, Haiti, Nicaragua, Trinidad and Tobago

*South America*= Brazil, Peru, Argentina, Colombia, Ecuador, Chile, Guyana

*Oceania*= Australia, Papua New Guinea, New Zealand

*Antarctica*= none

*Place of birth, country: total number of athletes (n=325)*

| **Country** | **n (%)** |
| --- | --- |
| Italy | 33 (10.1) |
| Brazil | 19 (5.8) |
| Poland | 14 (4.3) |
| India | 13 (4) |
| Peru | 12 (3.7) |
| United States of America | 12 (3.7) |
| Germany | 11 (3.4) |
| Ecuador | 10 (3.1) |
| Czech Republic | 9 (2.8) |
| United Kingdom of Great Britain and Northern Ireland | 9 (2.8) |
| Argentina | 8 (2.5) |
| France | 8 (2.5) |
| Colombia | 8 (2.5) |
| Japan | 7 (2.1) |
| Bahamas | 7 (2.1) |
| Norway | 7 (2.1) |
| Spain | 6 (1.8) |
| Estonia | 6 (1.8) |
| Chile | 6 (1.8) |
| Ukraine | 6 (1.8) |
| Latvia | 5 (1.5) |
| Austria | 5 (1.5) |
| Botswana | 5 (1.5) |
| Sri Lanka | 5 (1.5) |
| Canada | 5 (1.5) |
| Morocco | 5 (1.5) |
| Slovenia | 5 (1.5) |
| Mexico | 5 (1.5) |
| Australia | 4 (1.2) |
| Cuba | 4 (1.2) |
| Finland | 4 (1.2) |
| Turkey | 4 (1.2) |
| Lithuania | 4 (1.2) |
| Portugal | 4 (1.2) |
| Croatia | 4 (1.2) |
| Serbia | 3 (0.9) |
| Kazakhstan | 3 (0.9) |
| Jamaica | 3 (0.9) |
| Republic of Korea | 2 (0.6) |
| Belgium | 2 (0.6) |
| Haiti | 2 (0.6) |
| Hungary | 2 (0.6) |
| China | 2 (0.6) |
| South ‎Sudan | 2 (0.6) |
| Nigeria | 2 (0.6) |
| Denmark | 2 (0.6) |
| Guyana | 2 (0.6) |
| Slovakia | 2 (0.6) |
| Israel | 2 (0.6) |
| Nicaragua | 1 (0.3) |
| Trinidad and Tobago | 1 (0.3) |
| Singapore | 1 (0.3) |
| Algeria | 1 (0.3) |
| Switzerland | 1 (0.3) |
| South Africa | 1 (0.3) |
| United Arab Emirates | 1 (0.3) |
| Sudan | 1 (0.3) |
| Lesotho | 1 (0.3) |
| Bosnia and Herzegovina | 1 (0.3) |
| New Zealand | 1 (0.3) |
| Lebanon | 1 (0.3) |
| Sweden | 1 (0.3) |
| Papua New Guinea | 1 (0.3) |
| Romania | 1 (0.3) |

Results are reported as n (%).

*Represented federation in Lima World Championships: total athletes (n=325)*

| **Federation** | **n (%)** |
| --- | --- |
| Italy | 33 (10.1) |
| Brazil | 19 (5.8) |
| India | 14 (4.3) |
| Poland | 13 (4) |
| Peru | 12 (3.7) |
| Germany | 12 (3.7) |
| United States of America | 11 (3.4 |
| Ecuador | 10 (3.1) |
| United Kingdom of Great Britain and Northern Ireland | 9 (2.8) |
| Czech Republic | 9 (2.8) |
| France | 8 (2.5) |
| Argentina | 8 (2.5) |
| Spain | 8 (2.5) |
| Colombia | 8 (2.5) |
| Norway | 7 (2.1) |
| Bahamas | 7 (2.1) |
| Ukraine | 6 (1.8) |
| Austria | 6 (1.8) |
| Estonia | 6 (1.8) |
| Chile | 6 (1.8) |
| Slovenia | 5 (1.5) |
| Sri Lanka | 5 (1.5) |
| Latvia | 5 (1.5) |
| Canada | 5 (1.5) |
| Mexico | 5 (1.5) |
| Botswana | 5 (1.5) |
| Japan | 5 (1.5) |
| Portugal | 5 (1.5) |
| Turkey | 4 (1.2) |
| Cuba | 4 (1.2) |
| Croatia | 4 (1.2) |
| Finland | 4 (1.2) |
| Lithuania | 4 (1.2) |
| Morocco | 4 (1.2) |
| Australia | 4 (1.2) |
| Kazakhstan | 3 (0.9) |
| Jamaica | 3 (0.9) |
| Serbia | 3 (0.9) |
| Kenya | 3 (0.9) |
| Slovakia | 2 (0.6) |
| Israel | 2 (0.6) |
| Denmark | 2 (0.6) |
| Guyana | 2 (0.6) |
| Trinidad and Tobago | 2 (0.6) |
| Belgium | 2 (0.6) |
| China | 2 (0.6) |
| New Zealand | 2 (0.6) |
| South Africa | 2 (0.6) |
| Hungary | 2 (0.6) |
| Romania | 1 (0.3) |
| Algeria | 1 (0.3) |
| Republic of Korea | 1 (0.3) |
| British Virgin Island | 1 (0.3) |
| Sweden | 1 (0.3) |
| Singapore | 1 (0.3) |
| Nicaragua | 1 (0.3) |
| Switzerland | 1 (0.3) |
| Ethiopia | 1 (0.3) |
| Belize | 1 (0.3) |
| Lesotho | 1 (0.3) |
| Bosnia and Herzegovina | 1 (0.3) |
| Nigeria | 1 (0.3) |

Results are reported as n (%).

*Medical history. Results for the total number of athletes (n=325) and reported by sex.*

| **Variable*** | **Total**  **n= 325** | **Females**  **n=192** | **Males**  **n=133** |
| --- | --- | --- | --- |
| **Regular medication intake** |  |  |  |
| No | 317 (97.5) | 188 (97.9) | 129 (97) |
| Yes | 8 (2.5) | 4 (2.1) | 4 (3) |
| **Smoking** |  |  |  |
| No | 318 (97.8) | 190 (99) | 128 (96.2) |
| Yes | 7 (2.1) | 2 (1) | 5 (3.8) |
| **Health conditions** |  |  |  |
| None | 276 (84.9) | 158 (82.3) | 118 (88.7) |
| Respiratory and breathing issues like  asthma | 25 (7.7) | 17 (8.8) | 8 (6.0) |
| Recurrent urinary infections | 11 (3.4) | 8 (4.2) | 3 (2.3) |
| Constipation | 6 (1.8) | 5 (2.6) | 1 (0.7) |
| History of pelvic surgery | 2 (0.6) | 1 (0.5) | 1 (0.7) |
| Others | 5 (1.5) | 3 (1.6) | 2 (1.5) |
| Hypertension | 2 (0.6) | 1 (0.3) | 1 (0.3) |
| Diabetes | 1 (0.3) | 1 (0.3) | - |
| Hyperthyroidism | 1 (0.3) | 1 (0.3) | - |
| Heart arrhythmia | 1 (0.3) | - | 1 (0.3) |
| **Muscle or bone injuries in lower belly or pelvic area** |  |  |  |
| No | 288 (88.6) | 173 (90.1) | 115 (86.5) |
| Yes | 37 (11.4) | 19 (9.9) | 18 (13.5) |
| Not specified | 13 (4) | 10 (5.2) | 3 (2.5) |
| Groin pain | 10 (3) | 5 (2.6) | 5 (3.8) |
| Hamstring muscle injury | 8 (2.5) | 1 (0.5) | 7 (5.3) |
| Testicular pain | 1 (0.3) | - | 1 (0.7) |
| “Psoas pain” | 3 (0.9) | 2 (1.0) | 1 (0.7) |
| Hip fracture | 2 (0.6) | 1 (0.5) | 1 (0.7) |
| **Number of stress fractures during the career** |  |  |  |
| None | 256 (78.8) | 152 (79.2) | 104 (78) |
| One to three | 60 (18.5) | 34 (17.7) | 26 (19.5) |
| More than three | 9 (2.8) | 6 (3.1) | 3 (2.3) |

Results are reported as n (%).

* Respiratory and breathing issues like asthma; Recurrent urinary infections (>2 times per year, diagnosed with urine tests). “Psoas pain” = terminology reported by athletes, open question.

1. **SPORTS-RELATED CHARACTERISTICS**

*Sports-related characteristics: training and competitions volume in Athletics. Results for the total number of athletes (n=325) and reported by sex.*

| **Variables*** | **Total**  **n=325** | **Females**  **n= 192** | **Males**  **n= 133** |
| --- | --- | --- | --- |
| Training hours/day | 2.8 ± 1.2 | 2.9 ± 1.2 | 2.7 ± 1.1 |
| Number of training sessions/week | 6 ± 2 | 6.2 ± 2 | 5.7 ± 1.9 |
| Number of competitions/month | 2.6 ± 1.8 | 2.7 ± 2 | 2.4 ± 1.5 |
| Number of high-level competitions/season | 2.9 ± 1.8 | 2.8 ± 1.8 | 3 ± 1.9 |
| Number of years as a competitive athlete | 4.9 ± 2.5 | 5 ± 2.5 | 4.7 ± 2.5 |
| Participation in other sports or training activities |  |  |  |
| No other activities in addition to athletics | 245 (75.4) | 138 (71.9) | 107 (80.4) |
| Other activities including gym | 80 (24.6) | 54 (28.1) | 26 (19.5) |
| Competition in other sports to athletics | 17 (5.2) | 9 (4.7) | 8 (6) |

Results are reported as n (%) and mean ± SD.

* Number of training session per day or per week, considering also additional training such as gym session or others. Number of high-level competitions per season (e.g. international).

*Events. Official Timetable, Ilma 2024 World Athletics U20 Championships.**


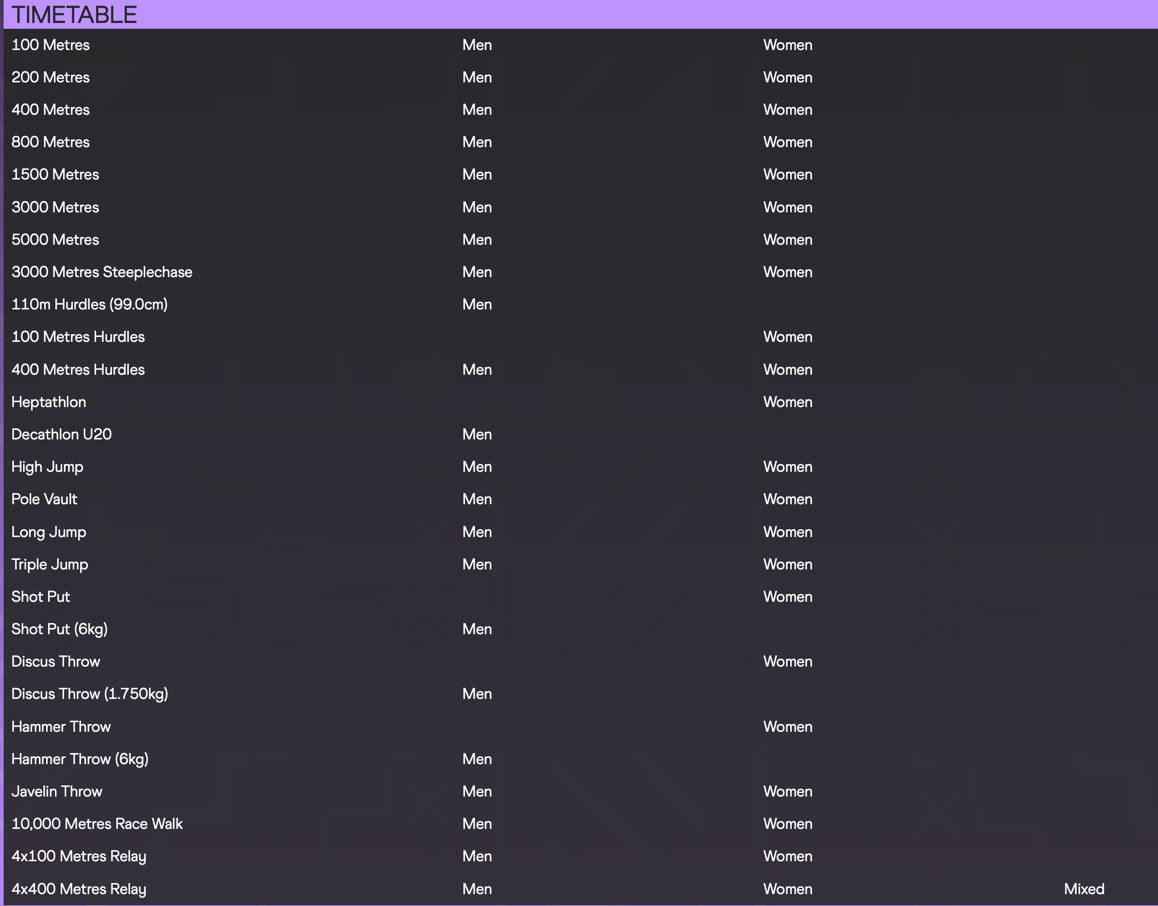


*Events. Results for the total number of athletes (n=325) and reported by sex.*

| **Variables** | **Total**  **n=325** | **Females**  **n=192** | **Males**  **n=133** |
| --- | --- | --- | --- |
| 100 Metres | 72 (22.1) | 35 (18.2) | 37 (27.8) |
| 200 Metres | 55 (16.9) | 31 (16.1) | 24 (18) |
| 400 Metres | 70 (21.5) | 39 (20.3) | 31 (23.3) |
| 800 Metres | 31 (9.5) | 23 (12) | 8 (6) |
| 1500 Metres | 21 (6.5) | 13 (6.8) | 8 (6) |
| 5000 Metres | 11 (3.4) | 8 (4.2) | 3 (2.3) |
| 10000m Race Walk | 26 (8) | 12 (6.2) | 14 (10.5) |
| 3000 Metres Steeplechase | 12 (3.7) | 6 (3.1) | 6 (4.5) |
| 100/110 Metres Hurdles | 32 (9.8) | 20 (10.4) | 12 (9) |
| 400 Metres Hurdles | 18 (5.5) | 10 (5.2) | 8 (6) |
| Heptathlon | 7 (2.1) | 7 (3.6) | 0 (0) |
| Decathlon | 2 (0.6) | 1 (0.5) | 1 (0.7) |
| High jump | 9 (2.8) | 4 (2.1) | 5 (3.8) |
| Pole vault | 13 (4) | 12 (6.2) | 1 (0.7) |
| Long jump | 12 (3.7) | 10 (5.2) | 2 (1.5) |
| Triple jump | 11 (3.4) | 9 (4.7) | 2 (1.5) |
| Shot Put | 11 (3.4) | 6 (3.1) | 5 (3.8) |
| Discus Throw | 9 (2.8) | 5 (2.6) | 4 (3) |
| Hammer Throw | 4 (1.2) | 2 (1.0) | 2 (1.5) |
| Javelin Throw | 7 (2.1) | 5 (2.6) | 2 (1.5) |

Results are reported as n (%).

*Discipline classification. Results for the total number of athletes (n=325) and reported by sex.*

| **Variables*** | **Total**  **n=325** | **Females**  **n=192** | **Males**  **n=133** |
| --- | --- | --- | --- |
| Sprints | 128 (39.4) | 67 (34.9) | 61 (45.9) |
| Hurdles | 39 (12) | 25 (13) | 14 (10.5) |
| Middle distance runs | 39 (12) | 26 (13.5) | 13 (9.8) |
| Jumps | 38 (11.7) | 28 (14.6) | 10 (7.5) |
| Throws | 29 (8.9) | 17 (8.8) | 12 (9) |
| Race walking | 26 (8) | 12 (6.2) | 14 (10.5) |
| Long-distance runs | 19 (5.8) | 11 (5.7) | 8 (6) |
| Combined events | 7 (2.1) | 6 (3.1) | 1 (0.7) |

Results are reported as n (%).

**Athletic event group classification*, based on the Athletics Olympic programme and based on previous Athletics studies [1,2]:

*Sprints* (60, 100, 200 and 400 m) and relays (4 × 100 and 4 × 400);

*Middle distance runs* (800–1500 m);

*Long-distance runs* (3000–10 000 m) including steeplechase (2000 and 3000 m steeplechase);

*Cross-country races;* *Marathon, half-marathon and road races;*

*Race walking* (5, 10, 20 and 50 km);

*Hurdles* (60, 100, 110 and 400 m hurdles);

*Jumps* (high, long, triple and pole vault);

*Throws* (discus, javelin, hammer and shot put);

*Combined events* (pentathlon, heptathlon, octathlon and decathlon).

For the analysis, the following criteria were applied:

- Athletes competing in multiple events were categorized based on the longest distance run (e.g., an athlete competing in both 400m and 800m was classified as "Middle distance runs").
- Events were categorized as "Hurdles" if the athlete exclusively competed in hurdle events.

*[1] Timpka T, Alonso JM, Jacobsson J, Junge A, Branco P, Clarsen B, Kowalski J, Mountjoy M, Nilsson S, Pluim B, Renström P, Rønsen O, Steffen K, Edouard P. Injury and illness definitions and data collection procedures for use in epidemiological studies in Athletics (track and field): consensus statement. Br J Sports Med. 2014 Apr;48(7):483-90. doi: 10.1136/bjsports-2013-093241.*

*[2] Bermon S, Adami PE, Dahlström Ö, Fagher K, Hautala J, Ek A, Anderson C, Jacobsson J, Svedin CG, Timpka T. Lifetime Prevalence of Verbal, Physical, and Sexual Abuses in Young Elite Athletics Athletes. Front Sports Act Living. 2021 May 31;3:657624. doi: 10.3389/fspor.2021.657624. PMID: 34136805; PMCID: PMC8200562.*

1. **PELVIC FLOOR DOMAIN**

*Pelvic floor and related habits. Results for the total number of athletes (n=325) and reported by sex.*

| **Variable** | **Total**  **n=325** | **Females**  **n= 192** | **Males**  **n= 133** |
| --- | --- | --- | --- |
| **Difficulty starting urination in daily life** |  |  |  |
| No | 298 (91.7) | 180 (93.7) | 118 (88.7) |
| Yes | 27 (8.3) | 12 (6.3) | 15 (11.3) |
| **Need to push or strain during bowel movement in daily life** |  |  |  |
| No | 288 (88.6) | 164 (85.4) | 124 (93.2) |
| Yes | 37 (11.4) | 28 (14.6) | 9 (6.8) |
| **Going to the toilet *before* training or competing** |  |  |  |
| No | 89 (27.4) | 38 (19.8) | 51 (38.3) |
| Yes | 236 (72.6) | 154 (80.2) | 82 (61.6) |
| **Reducing liquid intake *during* training or competing** |  |  |  |
| No | 241 (74.1) | 140 (72.9) | 101 (75.9) |
| Yes | 84 (25.8) | 52 (27.1) | 32 (24.1) |
| **Going to the toilet frequently *during* training** |  |  |  |
| No | 208 (64) | 113 (58.8) | 95 (71.4) |
| Yes | 117 (36) | 79 (41.2) | 38 (28.6) |
| **Going to the toilet frequently *during* event or competition** |  |  |  |
| No | 193 (59.4) | 104 (54.2) | 89 (66.9) |
| Yes | 132 (40.6) | 88 (45.8) | 44 (33.1) |
| **Consumption of gels/drinks or energy supplements with caffeine** |  |  |  |
| No | 217 (66.8) | 130 (67.7) | 87 (65.4) |
| Yes | 108 (33.2) | 62 (32.3) | 46 (34.6) |
| **Use of sanitary pads/products during physical activities** |  |  |  |
| No | 224 (68.9) | 107 (55.7) | 117 (88.7) |
| Yes | 101 (31.1) | 85 (44.3) | 16 (11.3) |

Results are reported as n (%).

*Pelvic floor domain, knowledge and sports practice. Results for the total number of athletes (n=325) and reported by sex.*

| **Variable** | **Total**  **n=325** | **Females**  **n= 192** | **Males**  **n= 133** |
| --- | --- | --- | --- |
| **Pelvic floor health awareness** |  |  |  |
| No | 230 (70.8) | 127 (66.1) | 103 (77.4) |
| Yes | 95 (29.2) | 65 (33.8) | 30 (22.6) |
| **Pelvic floor dysfunction awareness** |  |  |  |
| No | 256 (78.8) | 146 (76) | 110 (82.7) |
| Yes | 69 (21.2) | 46 (24) | 23 (17.3) |
| **Pelvic floor screening or evaluation during sports medicine examinations** |  |  |  |
| No | 286 (88) | 169 (88.0) | 117 (88) |
| Yes | 39 (12) | 23 (12) | 16 (12) |
| **Presence of a health professional within the team to discuss pelvic floor health** |  |  |  |
| No | 137 (42.1) | 75 (39.1) | 62 (46.6) |
| Yes | 116 (35.7) | 73 (38) | 43 (32.3) |
| Do not know | 72 (22.1) | 44 (22.9) | 28 (21) |
| **Presence of a health professional: kind of professional** |  |  |  |
| Sports physician | 61 (52.6) | 32 (43.8) | 29 (67.4) |
| Sports physiotherapist | 44 (37.9) | 34 (46.6) | 10 (23.3) |
| Urologist | 3 (2.6) | 2 (2.7) | 2 (4.6) |
| Uro-gynecologist | 3 (2.6) | 2 (2.7) | - |
| Pelvic floor physiotherapist | 3 (2.6) | 2 (2.7) | 1 (2.3) |
| Other specialists | 2 (1.7) | 1 (1.4) | 1 (2.3) |

Results are reported as n (%).

* Presence of a health professional: kind of professional. Results from athletes who answered *“yes”* at the previous questions (n=116).

1. **PELVIC FLOOR DYSFUNCTION**

*Pelvic floor dysfunction (PFD) symptoms in daily life. Results for the total number of athletes (n=325) and reported by sex.*

| **Variable*** | **Total**  **n=325** | **Females**  **n= 192** | **Males**  **n= 133** |
| --- | --- | --- | --- |
| **Urinary incontinence (any type)** |  |  |  |
| No | 294 (90) | 165 (85.9) | 129 (97) |
| Yes | 31 (9.5) | 27 (14.1) | 4 (3) |
| **Overactive bladder syndrome** |  |  |  |
| No | 262 (80.6) | 144 (75) | 118 (88.7) |
| Yes | 63 (19.4) | 48 (25) | 15 (11.3) |
| **Anal incontinence** |  |  |  |
| No | 289 (88.9) | 166 (86.5) | 123 (92.5) |
| Yes | 36 (11.1) | 26 (13.5) | 10 (7.5) |
| **Pelvic pain** |  |  |  |
| No | 264 (81.2) | 143 (74.5) | 121 (91) |
| Yes | 61 (18.8) | 49 (25.5) | 12 (9) |
| **Pelvic organ prolapse (POP)** |  |  |  |
| No | - | 180 (93.7) | - |
| Yes | - | 12 (6.3) | - |
| **Number of athletes who reported at least one symptom** | 117 (36) | 91 (47.4) | 26 (19.5) |

Results are reported as n (%).

* Questions extracted from the PFD-SENTINEL tool, PART A. Giagio S, Salvioli S, Innocenti T, Gava G, Vecchiato M, Pillastrini P, Turolla A. PFD-SENTINEL: Development of a screening tool for pelvic floor dysfunction in female athletes through an international Delphi consensus. Br J Sports Med. 2023 Jul;57(14):899-905. doi: 10.1136/bjsports-2022-105985. Epub 2022 Dec 14. PMID: 36517214.

*ICIQ-UI-SF: Results for the total number of athletes (n=31), experiencing urinary incontinence during daily life) and reported by sex.*

| **Variable** | **Total**  **n=31** | **Females**  **n=27** | **Males**  **n=4** |
| --- | --- | --- | --- |
| **When** |  |  |  |
| Leaks before you can get to the toilet | 10 (32.3) | 8 (29.6) | 2 (50) |
| Leaks when you cough or sneeze | 12 (38.7) | 12 (44.4) | 0 (0) |
| Leaks when you are asleep | 1 (3.2) | 1 (3.70) | 0 (0) |
| Leaks when you are physically active/exercising | 19 (61.3) | 18 (66.7) | 1 (25) |
| Leaks when you have finished urinating and are dressed | 3 (9.7) | 3 (11.1) | 0 (0) |
| Leaks for no obvious reason | 4 (12.9) | 4 (14.8) | 0 (0) |
| Leaks all the time | 1 (3.2) | 0 (0) | 1 (25) |
| **How often / frequency** |  |  |  |
| About once a week or less often | 15 (48.4) | 15 (55.6) | 0 (0) |
| Two or three times a week | 7 (22.6) | 5 (18.5) | 2 (50) |
| About once a day | 5 (16.1) | 3 (11.1) | 2 (50) |
| Several times a day | 3 (9.7) | 3 (11.1) | 0 (0) |
| All the time | 1 (3.2) | 1 (3.7) | 0 (0) |
| **How much urine** |  |  |  |
| A small amount | 21 (67.7) | 18 (66.7) | 3 (75) |
| A moderate amount | 9 (29) | 8 (29.6) | 1 (25) |
| A large amount | 1 (3.2) | 1 (3.3) | (0) |
| **How much does leaking urine interfere with everyday life** | 3.6 ± 2.6 | 3.4 ± 2.7 | 5 ± 0.8 |
| **ICIQ-SF** |  |  |  |
| Total score | 8.2 ± 3.8 | 8 ± 4 | 10 ± 1.4 |
| **ICIQ-SF** |  |  |  |
| Slight | 12 (38.7) | 12 (44.4) | 0 (0) |
| Moderate | 14 (45.2) | 10 (37) | 4 (100) |
| Severe | 5 (16.1) | 5 (18.5) | 0 (0) |
| Very severe | 0 (0) | 0 (0) | 0 (0) |

Results are reported as n (%) and mean ± SD.

*Urinary incontinence (any type) in daily life.*

| **Variable** | **Total**  **n=31** | **Females**  **n=27** | **Males**  **n=4** |
| --- | --- | --- | --- |
| **Impact in daily life** |  |  |  |
| No influence | 25 (80.6) | 21 (77.8) | 4 (100) |
| Feel embarrassed, afraid it may happen again | 20 (64.5) | 16 (59.3) | 4 (100) |
| Afraid of visible leakage | 19 (61.3) | 17 (63) | 2 (50) |
| Feel frustrated, annoyed, worried | 16 (51.6) | 15 (55.6) | 1 (25) |

Results are reported as n (%).

*Pelvic floor dysfunction (PFD) in Athletics. Results for the total number of athletes (n=325) and reported by sex.*

| **Variable** | **Total**  **n=325** | **Females**  **n= 192** | **Males**  **n= 133** |
| --- | --- | --- | --- |
| **Urinary incontinence** |  |  |  |
| No | 283 (87.1) | 157 (81.8) | 126 (94.7) |
| Yes | 42 (12.9) | 35 (18.2) | 7 (5.3) |
| **Overactive Bladder Syndrome** |  |  |  |
| No | 258 (79.4) | 144 (75) | 114 (85.7) |
| Yes | 67 (20.6) | 48 (25) | 19 (14.3) |
| **Anal incontinence** |  |  |  |
| No | 294 (90.5) | 170 (88.5) | 124 (93.2) |
| Yes | 31 (9.5) | 22 (11.5) | 9 (6.8) |
| **Pelvic pain** |  |  |  |
| No | 277 (85.2) | 157 (81.8) | 120 (90.2) |
| Yes | 48 (14.8) | 35 (18.2) | 13 (9.8) |
| **Number of athletes who reported at least one symptom** | 111 (34.1) | 79 (41.1) | 32 (24.1) |

Results are reported as n (%).

*Athletics-related urinary incontinence.*

| **Variable** | **Total**  **n=42** | **Females**  **n=35** | **Males**  **n= 7** |
| --- | --- | --- | --- |
| **Trigger – Athletics** |  |  |  |
| Training | 23 (54.8) | 18 (51) | 5 (71.4) |
| Competitions | 20 (47.6) | 17 (48.6) | 3 (42.9) |
| Both | 12 (28.6) | 10 (28.6) | 2 (28.6) |
| **Trigger – activity** |  |  |  |
| Specific athletics- activities (jumping, sprinting, changing direction) | 18 (42.9) | 17 (48.6) | 1 (14.3) |
| Other sports-activities (for example lifting, gym) | 1 (2.4) | 1 (2.9) | 0 (0) |
| **Impact on emotion sphere, during competitions or training** |  |  |  |
| Feel frustrated, annoyed, worried | 17 (40.5) | 14 (40) | 3 (42.9) |
| No emotional influence | 11 (26.2) | 9 (25.7) | 2 (28.6) |
| Afraid of visible leakage | 10 (23.8) | 10 (28.6) | 0 (0) |
| Feel embarrassed, afraid it may happen again | 9 (21.4) | 7 (20) | 2 (28.6) |
| Afraid that urine loss will smell | 3 (7.1) | 3 (8.6) | 0 (0) |
| **Impact on athletic performance** |  |  |  |
| No influence | 19 (45.2) | 16 (45.7) | 3 (42.9) |
| Loss of concentration | 15 (35.7) | 14 (40) | 1 (14.3) |
| I move differently | 6 (14.3) | 6 (17.1) | 0 (0) |
| Make mistakes in the performance | 4 (9.6) | 2 (5.7) | 2 (28.6) |

Results are reported as n (%).

*Pelvic floor dysfunction (PFD) aggregated data. Descriptive analysis for the total number of athletes (n=325) and reported by sex.*

| **Variable*** | **Total**  **n=325** | **Females**  **n= 192** | **Males**  **n= 133** |
| --- | --- | --- | --- |
| At least one PFD in life | 117 (36) | 91 (47.4) | 26 (19.6) |
| At least one PFD in Athletics | 111 (34.2) | 79 (41.1) | 32 (24.1) |
| Athletics-related UI | 42 (12.9) | 35 (18.2) | 7 (5.3) |
| **Overall number of athletes**  **who reported at least one PFD** | 142 (43.7) | 103 (53.7) | 39 (29.3) |

Results are reported as n (%).

^*^ PFD=Pelvic Floor Dysfunction; UI= Urinary Incontinence.

*Pelvic floor dysfunction (PFD) aggregated data. Analysis per discipline and reported by sex.*

| **Variable*** | **Total**  **n=142** | **Females**  **n=103** | **Males**  **n=39** |
| --- | --- | --- | --- |
| Sprints | 44 (31) | 30 (29.1) | 14 (35.9) |
| Hurdles | 20 (14.1) | 16 (15.5) | 4 (10.3) |
| Middle distance runs | 19 (13.4) | 14 (13.6) | 5 (12.8) |
| Jumps | 17 (12) | 15 (14.6) | 2 (5.1) |
| Long-distance runs | 14 (9.9) | 7 (6.8) | 7 (17.9) |
| Throws | 12 (8.4) | 11 (10.7) | 1 (2.6) |
| Race walking | 12 (8.4) | 7 (6.8) | 5 (12.8) |
| Combined events | 4 (2.8) | 4 (3.9) | 0 |

Results are reported as n (%).

*Pelvic floor dysfunction (PFD) symptoms: aggregated data. Standardised prevalence rates and reported by sex.*

| **Variable*** | **Total (%)** | **Females (%)** | **Males (%)** |
| --- | --- | --- | --- |
| Sprints | 34.4 | 44.8 | 22.9 |
| Hurdles | 51.3 | 64 | 28.6 |
| Middle distance runs | 48.7 | 53.8 | 38.5 |
| Jumps | 44.7 | 53.6 | 20 |
| Throws | 41.4 | 64.7 | 8.3 |
| Race walking | 46.1 | 58.3 | 35.7 |
| Long-distance runs | 73.7 | 63.6 | 87.5 |
| Combined events | 57.1 | 66.7 | 0 |

Results are reported as percentage, %.

*Standardised prevalence rates were calculated to compare the proportion of athletes with symptoms across disciplines, accounting for differences in sample size = number of athletes with symptoms/number of athletes per discipline) x 100.

*Pelvic floor dysfunction (PFD) symptoms: aggregated data. Standardised prevalence rates per 100 athletes per continent.*

| **Variable*** | **Total number of athletes** | **Number of athletes with PFD symptoms** | **Total (%)** |
| --- | --- | --- | --- |
| Oceania | 6 | 4 | 66.7 |
| Asia | 42 | 20 | 47.6 |
| South America | 65 | 27 | 41.5 |
| Europe | 154 | 70 | 45.4 |
| Africa | 18 | 8 | 44.4 |
| North America | 40 | 14 | 35 |

*Standardised prevalence rates were calculated to compare the proportion of athletes with symptoms across continents, accounting for differences in sample size = number of athletes with symptoms/number of athletes per continent) x 100.

1. **FEMALE GYNECOLOGICAL DOMAIN**

*Uro-gynecological domain. Results among female athletes.*

| **Variable** | **n= 192** |
| --- | --- |
| **At least one gynaecological evaluation** |  |
| No | 115 (59.9) |
| Yes | 77 (40.1) |
| **Regular gynaecological check-ups (at least once a year)** |  |
| No | 142 (74) |
| Yes | 48 (25) |
| **Gynaecological medical conditions** |  |
| No gynaecological problems | 169 (88) |
| Polycystic ovarian syndrome | 10 (5.2) |
| Gynaecological disorders related to menstrual disorders | 5 (2.6) |
| Endometriosis | 3 (1.6) |
| Do not know | 3 (1.6) |
| Recurrent vaginal infection | 2 (1) |
| **Age of menarche^a^** | 13.5 ± 1.7 |
| **Menstrual cycle** |  |
| Regular menstrual cycle | 114 (59.4) |
| Irregular menstrual cycle | 60 (31.2) |
| Regular, with hormonal medications or other contraceptive use | 14 (7.3) |
| Not applicable, no menstrual cycle for any reason | 4 (2.1) |
| **Change in menstrual cycle when increase of exercise intensity, frequency or duration** |  |
| No | 106 (55.2) |
| Yes | 76 (39.6) |
| Not applicable, no menstrual cycle for any reason | 10 (5.1) |
| **Painful menstrual cycle** |  |
| No | 99 (51.6) |
| Yes | 89 (46.3) |
| Not applicable, no menstrual cycle for any reason | 4 (2.1) |
| **Pain during vaginal tampons use** |  |
| No | 105 (54.7) |
| No, because I do not use vaginal tampons | 64 (33.3) |
| Yes | 23 (12) |
| **Use of hormonal medication or other contraceptive methods** |  |
| No | 161 (83.8) |
| Yes | 31 (16.1) |
| **Reason for hormonal medication or other contraceptive methods use ^b^** |  |
| Regulate menstruation cycle | 13 (41.9) |
| Avoid pregnancy | 10 (32.3) |
| Both reasons | 8 (25.8) |

Results are reported as n (%).

^a^ Number of females athletes was 191; one athlete reported primary amenorrhea. See main manuscript.

^b^ Analysis was conducted among athletes who answered “yes” to the previous question (n=31).

1. **MANAGEMENT OF PFD**

*Pelvic floor symptoms management. Results for athletes who reported at least one symptom of pelvic floor dysfunction (PFD) during daily life and/or athletic activities (n = 142), with results reported by sex.*

| **Variable *** | **n=142** | **Females**  **n= 103** | **Males**  **n= 39** |
| --- | --- | --- | --- |
| **Discussion symptoms with someone** |  |  |  |
| No | 111 (78.2) | 78 (75.7) | 33 (84.6) |
| Yes | 31 (21.8) | 25 (24.3) | 6 (15.4) |
| Healthcare professionals | 15 (48.4) | 13 (52) | 2 (33.3) |
| Family/parents | 15 (48.4) | 13 (52) | 2 (33.3) |
| Coach | 10 (32.3) | 8 (32) | 2 (33.3) |
| Teammates | 5 (16.1) | 4 (16) | 1 (16.7) |
| **Strategies to mitigate symptoms** |  |  |  |
| No strategies | 119 (83.8) | 85 (82.5) | 34 (87.2) |
| Use pads | 18 (12.7) | 14 (13.6) | 4 (10.3) |
| Restrict fluids | 6 (4.2) | 4 (3.9) | 2 (5.1) |
| Preventive urination | 1 (0.7) | 1 (1) |  |
| Pelvic floor exercises | 1 (0.7) | 1 (1) |  |
| **Specialised assessment** |  |  |  |
| No | 135 (95.1) | 98 (95.1) | 37 (94.9) |
| Yes | 7 (4.9) | 5 (4.8) | 2 (5.1) |
| **Therapy or intervention** |  |  |  |
| No | 122 (85.9) | 88 (85.4) | 34 (87.2) |
| Yes | 20 (14.1) | 15 (14.6) | 5 (12.8) |

Results are reported as n (%).

* Therapy or intervention for PFD (e.g. physiotherapy exercises, medications). Discussion symptoms: results among athletes who reported to have discussed symptoms with someone (n=31).
