# Supplementary material for: Nearly half of 325 athletes reported pelvic floor symptoms: a cross-sectional study at the Lima 2024 World Athletics U20 Championships
Source: BMJ Open Sport Exerc Med. 2025 Jul 25;11(3):e002564. doi: 10.1136/bmjsem-2025-002564 (PMC12306240; doi:10.1136/bmjsem-2025-002564)
Supplement: online supplemental file 4 [file bmjsem-11-3-s004.pdf]

SUPPLEMENTARY FILE 4 A-C. Statistically significant differences between symptomatic and asymptomatic athletes: combined sample of males and females (A), females (B), and males (C).

A. MALE AND FEMALE ATHLETES

TABLE A1. Analysis among males and females (n=325). Differences between symptomatic and asymptomatic athletes *in daily life.*

	**Variables[a]**	**Level**	**SYMPTOMATIC (n=117)**	**ASYMPTOMATIC (n = 208)**	***p*-value**
General characteristics	Sex	Male<br>Female	26 (22.2)<br>91 (77.8)	107 (51.4)<br>101 (48.6)	<.001
Medical history	Respiratory and breathing issues	No<br>Yes	102 (87.2)<br>15 (12.8)	198 (95.2)<br>10 (4.8)	.009
PF domain	PF awareness	No<br>Yes	66 (56.4)<br>51 (43.6)	164 (78.8)<br>44 (21.2)	<.001
	PFD awareness	No<br>Yes	80 (68.4)<br>37 (31.6)	176 (84.6)<br>32 (15.4)	<.001
	Need to push or strain during bowel movement in daily life	No<br>Yes	92 (78.6)<br>25 (21.4)	196 (94.2)<br>12 (5.8)	<.001
	Going to the toilet frequently *during* training	No<br>Yes	61 (52.1)<br>56 (47.9)	147 (70.7)<br>61 (29.3)	<.001
	Going to the toilet *before* training or competing	No<br>Yes	23 (19.7)<br>94 (80.3)	66 (31.7)<br>142 (68.3)	.019
	Caffeine gels/drinks or energy supplements consumption	No<br>Yes	70 (59.8)<br>47 (40.2)	147 (70.7)<br>61 (29.3)	.046
Sports-related characteristics	Event type: long distance runs	No<br>Yes	104 (88.9)<br>13 (11.1)	202 (97.1)<br>6 (2.9)	.002
	Participation in other sports or trainings	No<br>Yes	79 (67.5)<br>38 (32.5)	166 (79.8)<br>42 (20.2)	.014

Results are reported as n (%).
In the present table, only statistically significant association (p-value <0.05) are reported.
When not otherwise specified, the Chi-Square Test was used.

[a] PF = Pelvic Floor. PFD= Pelvic Floor Dysfunction. Pelvic floor awareness = Pelvic floor anatomy and function awareness.

TABLE A2. Analysis among males and females (n=325). Differences between symptomatic and asymptomatic athletes in *Athletics.*

	Variables [a]	Level	SYMPTOMATIC (n=111)	ASYMPTOMATIC (n=214)	*p*-value
General characteristics	Sex	Male	32 (28.8)	101 (47.2)	.001
		Female	79 (71.2)	113 (52.8)	
	BMI (kg/m^2)	Median [Min – Max]	20.3 [15 – 30.9]	20.9 [16 – 39.2]	.003[b]
		IQR	15.9	23.2	
PF domain	PF awareness	No	69 (62.2)	161 (75.2)	.014
		Yes	42 (37.8)	53 (24.8)	
	PFD awareness	No	176 (82.2)	80 (72.1)	.033
		Yes	38 (17.8)	31 (27.9)	
	Need to push or strain during bowel movement in daily life	No	87 (78.4)	201 (93.9)	<.001
		Yes	24 (21.6)	13 (6.1)	
	Going to the toilet frequently *during* training	No	60 (54.1)	148 (69.2)	.007
		Yes	51 (45.9)	66 (30.8)	
	Difficulty starting urination in daily life	No	96 (86.5)	202 (94.2)	.014
		Yes	15 (13.5)	12 (5.6)	
	Going to the toilet frequently *during* event or competition	No	136 (63.6)	57 (51.4)	.034
		Yes	78 (36.4)	54 (48.6)	
	Caffeine gels/drinks or energy supplements consumption	No	66 (59.5)	151 (70.6)	.044
		Yes	45 (40.5)	63 (29.4)	
Sports-related characteristics	Event type: throws	No	106 (95.5)	190 (88.8)	.044
		Yes	5 (4.5)	24 (11.2)	

Results are reported as n (%), and median, range [minimum–maximum], and interquartile range (IQR).
In the present table, only statistically significant association (p-value <0.05) are reported.

[a] PF = Pelvic Floor. PFD= Pelvic Floor Dysfunction. Pelvic floor awareness = Pelvic floor anatomy and function awareness.
[b] Mann-Whitney test was used. When not otherwise specified, the Chi-Square Test was used.

TABLE A3. Analysis among males and females (n=325). Differences between symptomatic (*Athletics-related urinary incontinence*) and asymptomatic athletes.

	**Variables[a]**	**Level**	**SYMPTOMATIC (n=42)**	**ASYMPTOMATIC (n = 283)**	***p*-value**
General characteristics	Sex	Male	7 (16.7)	126 (44.5)	<.001
		Female	35 (83.3)	157 (55.5)	
	BMI (kg/m^2)	Median [Min – Max]	19.6 [16.8 – 28.6]	19.6 [16.8 – 28.6]	<.001[b]
		IQR	11.8	11.8	
PF domain	PF awareness	No	22 (52.4)	208 (73.5)	.005
		Yes	20 (47.6)	75 (26.5)	
	PFD awareness	No	28 (66.7)	228 (80.6)	.040
		Yes	14 (33.3)	55 (19.4)	
	Reducing liquid intake	No	23 (54.8)	218 (77)	.002
		Yes	19 (45.2)	65 (23)	
	Going to the toilet frequently *during* training	No	20 (47.6)	188 (66.4)	.018
		Yes	22 (52.4)	95 (33.6)	
Sports-related characteristics	Event type: middle distance runs	No	32 (76.2)	254 (89.9)	.012
		Yes	10 (23.8)	29 (10.2)	

Results are reported as n (%), and median, range [minimum–maximum], and interquartile range (IQR).
In the present table, only statistically significant association (p-value <0.05) are reported.

[a] PF= Pelvic Floor. PFD= Pelvic Floor Dysfunction. Pelvic floor awareness = Pelvic floor anatomy and function awareness.
[b] Mann-Whitney test was used. When not otherwise specified, the Chi-Square Test was used.

B. FEMALE ATHLETES

TABLE B1. Analysis among females (n=192). Differences between symptomatic and asymptomatic athletes *in daily life.*

	Variables [a,b]	Level	SYMPTOMATIC (n=91)	ASYMPTOMATIC (n =101)	*p*-value
Medical history	Pelvic injury	No	77 (84.6)	96 (95)	.016
		Yes	14 (15.4)	5 (5)	
	Respiratory and breathing issues	No	79 (86.8)	96 (95)	.045
		Yes	12 (13.2)	5 (5)	
PF domain	PF awareness	No	47 (51.6)	80 (79.2)	<.001
		Yes	44 (48.4)	21 (20.8)	
	PFD awareness	No	60 (65.9)	86 (85.1)	.002
		Yes	31 (34.1)	15 (14.9)	
	Need to push or strain during bowel movement in daily life	No	69 (75.8)	95 (94.1)	<.001
		Yes	22 (24.2)	6 (5.9)	
	Difficulty starting urination in daily life	No	82 (90.1)	98 (97)	.048
		Yes	9 (9.9)	3 (3)	
	Caffeine gels/drinks or energy supplements consumption	No	55 (60.4)	75 (74.3)	.041
		Yes	36 (39.6)	26 (25.7)	
Female health	Change in menstrual cycle	No	38 (45.8)	68 (68.7)	.002
		Yes	45 (54.2)	31 (31.3)	
	Hormonal medication or other contraceptive methods	No	71 (78)	90 (89.1)	.037
		Yes	20 (22)	11 (10.9)	

Results are reported as n (%).
In the present table, only statistically significant association (p-value <0.05) are reported.
When not otherwise specified, the Chi-Square Test was used.

[a] PF= Pelvic Floor. PFD= Pelvic Floor Dysfunction. Change in menstrual cycle = Change in menstrual cycle when increase of exercise intensity, frequency or duration. Pelvic injury = Muscle or bone injuries in lower belly or pelvic area. Pelvic floor awareness = Pelvic floor anatomy and function awareness.

TABLE B2. Analysis among females (n=192). Differences between symptomatic and asymptomatic athletes *in Athletics*.

	**Variables** [a]	**Level**	**SYMPTOMATIC (n=79)**	**ASYMPTOMATIC (n =113)**	***p*-value**
Medical history	Pelvic injury	No	67 (84.8)	106 (93.8)	.040
		Yes	12 (15.2)	7 (6.3)	
PF domain	Need to push or strain during bowel movement in daily life	No	60 (75.9)	104 (92)	.002
		Yes	19 (24.1)	9 (8)	
Female health	Change in menstrual cycle	No	32 (44.4)	74 (67.3)	.002
		Yes	40 (55.6)	36 (32.7)	

Results are reported as n (%).
In the present table, only statistically significant association (p-value <0.05) are reported.
When not otherwise specified, the Chi-Square Test was used.

[a] PF= Pelvic Floor. Change in menstrual cycle = Change in menstrual cycle when increase of exercise intensity, frequency or duration. Pelvic injury = Muscle or bone injuries in lower belly or pelvic area.

TABLE B3. Analysis among females (n=192). Differences between symptomatic (*Athletics-related urinary incontinence*) and asymptomatic athletes.

	Variables[a]	Level	SYMPTOMATIC (n=35)	ASYMPTOMATIC (n =157)	*p*-value
General characteristics	BMI (kg/m^2)	Median [Min – Max]	19.23 [16.8 – 28.6]	20.3 [16 – 31.1]	.015[b]
		IQR	11.80	15.1	
PF domain	PF awareness	No	16 (45.7)	111 (70.7)	.005
		Yes	19 (54.3)	46 (29.3)	
	PFD awareness	No	22 (62.9)	124 (79)	.043
		Yes	13 (37.1)	33 (21)	
	Reducing liquid intake	No	19 (54.3)	121 (77.1)	.006
		Yes	16 (45.7)	36 (22.9)	
Female health	Hormonal medication or other contraceptive methods	No	24 (68.6)	137 (87.3)	.007
		Yes	11 (31.4)	20 (12.7)	
Athletics-related characteristics	Training (hours/day)	Median [Min – Max]	2 [1.5 – 4]	3 [1 – 8]	.002
		IQR	2.5	7	

Results are reported as n (%), and median, range [minimum–maximum], and interquartile range (IQR).
In the present table, only statistically significant association (p-value <0.05) are reported.

[a] PF = Pelvic Floor. PFD= Pelvic Floor Dysfunction. PF awareness = Pelvic floor anatomy and function awareness.
[b] Mann-Whitney test was used. When not otherwise specified, the Chi-Square Test was used.

C. MALE ATHLETES

TABLE C1. Analysis among males (n=133). Differences between symptomatic and asymptomatic athletes in *daily life.*

	**Variables [a]**	**Level**	**SYMPTOMATIC (n=26)**	**ASYMPTOMATIC (n=107)**	***p*-value**
PF domain	Going to the toilet frequently *during* training	No<br>Yes	13 (50)<br>13 (50)	82 (76.6)<br>25 (23.4)	.007

Results are reported as n (%).
In the present table, only statistically significant association (p-value <0.05) are reported.
When not otherwise specified, the Chi-Square Test was used.
[a] PF = Pelvic Floor.

TABLE C2. Analysis among males (n=133). Differences between symptomatic and asymptomatic athletes in *Athletics*..

	**Variables [a]**	**Level**	**SYMPTOMATIC (n=32)**	**ASYMPTOMATIC (n=101)**	***p*-value**
General characteristics	BMI (kg/m^2)	Median [Min – Max]<br>IQR	20.9 [15 – 23.5]<br>8.5	22.1 [16.3 – 39.2]<br>22.9	.027[b]
Medical history	History of one to three stress fractures	No<br>Yes	21 (65.6)<br>11 (34.4)	86 (85.1)<br>15 (14.9)	0.015
PF domain	Going to the toilet frequently *during* training	No<br>Yes	18 (56.3)<br>14 (43.8)	77 (76.2)<br>24 (23.8)	.029

Results are reported as n (%), and median, range [minimum–maximum], and interquartile range (IQR).
In the present table, only statistically significant association (p-value <0.05) are reported.

[a] PF = Pelvic Floor.
[b] Mann-Whitney test was used. When not otherwise specified, the Chi-Square Test was used.

TABLE C3. Analysis among males (n=133). Differences between symptomatic (*Athletics-related urinary incontinence*) and asymptomatic athletes.

	Variables [a]	Level	SYMPTOMATIC (n=7)	ASYMPTOMATIC (n=126)	*p*-value
General characteristics	BMI (kg/m^2)	Median [Min – Max]<br>IQR	19.9 [18.7 – 20.8]<br>2.1	21.9 [15 – 39.2]<br>24.2	.005[b]

Results are reported as median, range [minimum–maximum], and interquartile range (IQR).
In the present table, only statistically significant association (p-value <0.05) are reported.
[b] Mann-Whitney test was used.
