# Supplementary material for: Nearly half of 325 athletes reported pelvic floor symptoms: a cross-sectional study at the Lima 2024 World Athletics U20 Championships
Source: BMJ Open Sport Exerc Med. 2025 Jul 25;11(3):e002564. doi: 10.1136/bmjsem-2025-002564 (PMC12306240; doi:10.1136/bmjsem-2025-002564)
Supplement: online supplemental file 5 [file bmjsem-11-3-s005.pdf]

SUPPLEMENTARY FILE 5. Statistical report example. Differences between symptomatic and asymptomatic athletes in the combined sample of males and female (n=325).

DIFFERENCES BETWEEN SYMPTOMATIC AND ASYMPTOMATIC ATHLETES IN DAILY LIFE.

A. Continuous variables

Variables	Level	SYMPTOMATIC (n=117)	ASYMPTOMATIC (n = 208)	*p*-value*
BMI (kg/m^2)	Median [Min – Max]	20.45 [15 - 31.10]	20.77 [16 – 39.20]	.085
	IQR	16.10	23.20	
Training (hours/day)	Median [Min – Max]	2 [1 – 6]	2.50 [1-8]	.388
	IQR	5	7	
Training (sessions/week)	Median [Min – Max]	6 [1 – 13]	6 [1 – 13]	.148
	IQR	12	12	

* Mann-Whitney test (non-parametric test) was used.

B. Categorial variables

sex * symptoms_life

Crosstab

			symptoms_life		
			no	1	Total
sex	male	Count	107_a	26_b	133
		Expected Count	85,1	47,9	133,0
		% within sex	80,5%	19,5%	100,0%
		% within symptoms_life	51,4%	22,2%	40,9%
		% of Total	32,9%	8,0%	40,9%
		Standardized Residual	2,4	-3,2	
	female	Count	101_a	91_b	192
		Expected Count	122,9	69,1	192,0
		% within sex	52,6%	47,4%	100,0%
		% within symptoms_life	48,6%	77,8%	59,1%
		% of Total	31,1%	28,0%	59,1%
		Standardized Residual	-2,0	2,6	
Total		Count	208	117	325
		Expected Count	208,0	117,0	325,0
		% within sex	64,0%	36,0%	100,0%
		% within symptoms_life	100,0%	100,0%	100,0%
		% of Total	64,0%	36,0%	100,0%

Each subscript letter denotes a subset of symptoms_life categories whose column proportions do not differ significantly from each other at the ,05 level.

Chi-Square Tests

	Value	df	Asymptotic Significance (2-sided)	Exact Sig. (2-sided)	Exact Sig. (1-sided)
Pearson Chi-Square	26,445[a]	1	<,001		
Continuity Correction[b]	25,250	1	<,001		
Likelihood Ratio	27,648	1	<,001		
Fisher's Exact Test				<,001	<,001
Linear-by-Linear Association	26,364	1	<,001		
N of Valid Cases	325				

a. 0 cells (0,0%) have expected count less than 5. The minimum expected count is 47,88.
b. Computed only for a 2x2 table

Symmetric Measures

		Value	Approximate Significance
Nominal by Nominal	Phi	,285	<,001
	Cramer's V	,285	<,001
	Contingency Coefficient	,274	<,001
N of Valid Cases		325	

medications * symptoms_life

Crosstab

			symptoms_life		
			no	1	Total
medications	no	Count	204_a	113_a	317
		Expected Count	202,9	114,1	317,0
		% within medications	64,4%	35,6%	100,0%
		% within symptoms_life	98,1%	96,6%	97,5%
		% of Total	62,8%	34,8%	97,5%
		Standardized Residual	,1	-,1	
	yes	Count	4_a	4_a	8
		Expected Count	5,1	2,9	8,0
		% within medications	50,0%	50,0%	100,0%
		% within symptoms_life	1,9%	3,4%	2,5%
		% of Total	1,2%	1,2%	2,5%
		Standardized Residual	-,5	,7	
Total		Count	208	117	325
		Expected Count	208,0	117,0	325,0
		% within medications	64,0%	36,0%	100,0%
		% within symptoms_life	100,0%	100,0%	100,0%
		% of Total	64,0%	36,0%	100,0%

Each subscript letter denotes a subset of symptoms_life categories whose column proportions do not differ significantly from each other at the ,05 level.

Chi-Square Tests

	Value	df	Asymptotic Significance (2-sided)	Exact Sig. (2-sided)	Exact Sig. (1-sided)
Pearson Chi-Square	,698[a]	1	,404		
Continuity Correction[b]	,214	1	,644		
Likelihood Ratio	,670	1	,413		
Fisher's Exact Test				,465	,314
Linear-by-Linear Association	,696	1	,404		
N of Valid Cases	325				

a. 1 cells (25,0%) have expected count less than 5. The minimum expected count is 2,88.
b. Computed only for a 2x2 table

Symmetric Measures

		Value	Approximate Significance
Nominal by Nominal	Phi	,046	,404
	Cramer's V	,046	,404
	Contingency Coefficient	,046	,404
N of Valid Cases		325	

smoking * symptoms_life

Crosstab

			symptoms_life		
			no	1	Total
smoking	no	Count	205_a	113_a	318
		Expected Count	203,5	114,5	318,0
		% within smoking	64,5%	35,5%	100,0%
		% within symptoms_life	98,6%	96,6%	97,8%
		% of Total	63,1%	34,8%	97,8%
		Standardized Residual	,1	-,1	
	yes	Count	3_a	4_a	7
		Expected Count	4,5	2,5	7,0
		% within smoking	42,9%	57,1%	100,0%
		% within symptoms_life	1,4%	3,4%	2,2%
		% of Total	0,9%	1,2%	2,2%
		Standardized Residual	-,7	,9	
Total		Count	208	117	325
		Expected Count	208,0	117,0	325,0
		% within smoking	64,0%	36,0%	100,0%
		% within symptoms_life	100,0%	100,0%	100,0%
		% of Total	64,0%	36,0%	100,0%

Each subscript letter denotes a subset of symptoms_life categories whose column proportions do not differ significantly from each other at the ,05 level.

Chi-Square Tests

	Value	df	Asymptotic Significance (2-sided)	Exact Sig. (2-sided)	Exact Sig. (1-sided)
Pearson Chi-Square	1,388[a]	1	,239		
Continuity Correction[b]	,609	1	,435		
Likelihood Ratio	1,320	1	,251		
Fisher's Exact Test				,257	,214
Linear-by-Linear Association	1,384	1	,239		
N of Valid Cases	325				

a. 2 cells (50,0%) have expected count less than 5. The minimum expected count is 2,52.
b. Computed only for a 2x2 table

Symmetric Measures

		Value	Approximate Significance
Nominal by Nominal	Phi	,065	,239
	Cramer's V	,065	,239
	Contingency Coefficient	,065	,239
N of Valid Cases		325	

pelvic_injury * symptoms_life

Crosstab

			symptoms_life		Total
			no	1	
pelvic_injury	no	Count	189a	99a	288
		Expected Count	184,3	103,7	288,0
		% within pelvic_injury	65,6%	34,4%	100,0%
		% within symptoms_life	90,9%	84,6%	88,6%
		% of Total	58,2%	30,5%	88,6%
		Standardized Residual	,3	-,5	
	yes	Count	19a	18a	37
		Expected Count	23,7	13,3	37,0
		% within pelvic_injury	51,4%	48,6%	100,0%
		% within symptoms_life	9,1%	15,4%	11,4%
		% of Total	5,8%	5,5%	11,4%
		Standardized Residual	-1,0	1,3	
Total		Count	208	117	325
		Expected Count	208,0	117,0	325,0
		% within pelvic_injury	64,0%	36,0%	100,0%
		% within symptoms_life	100,0%	100,0%	100,0%
		% of Total	64,0%	36,0%	100,0%

Each subscript letter denotes a subset of symptoms_life categories whose column proportions do not differ significantly from each other at the ,05 level.

Chi-Square Tests

	Value	df	Asymptotic Significance (2-sided)	Exact Sig. (2-sided)	Exact Sig. (1-sided)
Pearson Chi-Square	2,899[a]	1	,089		
Continuity Correction[b]	2,313	1	,128		
Likelihood Ratio	2,805	1	,094		
Fisher's Exact Test				,102	,066
Linear-by-Linear Association	2,890	1	,089		
N of Valid Cases	325				

a. 0 cells (0,0%) have expected count less than 5. The minimum expected count is 13,32.
b. Computed only for a 2x2 table

Symmetric Measures

		Value	Approximate Significance
Nominal by Nominal	Phi	,094	,089
	Cramer's V	,094	,089
	Contingency Coefficient	,094	,089
N of Valid Cases		325	

other_sports * symptoms_life

Crosstab

			symptoms_life		Total
			no	1	
other_sports	no	Count	166a	79b	245
		Expected Count	156,8	88,2	245,0
		% within other_sports	67,8%	32,2%	100,0%
		% within symptoms_life	79,8%	67,5%	75,4%
		% of Total	51,1%	24,3%	75,4%
		Standardized Residual	,7	-1,0	
	yes	Count	42a	38b	80
		Expected Count	51,2	28,8	80,0

	% within other_sports	52,5%	47,5%	100,0%
	% within symptoms_life	20,2%	32,5%	24,6%
	% of Total	12,9%	11,7%	24,6%
	Standardized Residual	-1,3	1,7	
Total	Count	208	117	325
	Expected Count	208,0	117,0	325,0
	% within other_sports	64,0%	36,0%	100,0%
	% within symptoms_life	100,0%	100,0%	100,0%
	% of Total	64,0%	36,0%	100,0%

Each subscript letter denotes a subset of symptoms_life categories whose column proportions do not differ significantly from each other at the ,05 level.

Chi-Square Tests

	Value	df	Asymptotic Significance (2-sided)	Exact Sig. (2-sided)	Exact Sig. (1-sided)
Pearson Chi-Square	6,091[a]	1	,014		
Continuity Correction[b]	5,447	1	,020		
Likelihood Ratio	5,955	1	,015		
Fisher's Exact Test				,016	,010
Linear-by-Linear Association	6,073	1	,014		
N of Valid Cases	325				

a. 0 cells (0,0%) have expected count less than 5. The minimum expected count is 28,80.
b. Computed only for a 2x2 table

Symmetric Measures

		Value	Approximate Significance
Nominal by Nominal	Phi	,137	,014
	Cramer's V	,137	,014
	Contingency Coefficient	,136	,014
N of Valid Cases		325	

pelvic_awareness * symptoms_life

Crosstab

			symptoms_life		
			no	1	Total
pelvic_awareness	no	Count	164_a	66_b	230
		Expected Count	147,2	82,8	230,0
		% within pelvic_awareness	71,3%	28,7%	100,0%
		% within symptoms_life	78,8%	56,4%	70,8%
		% of Total	50,5%	20,3%	70,8%
		Standardized Residual	1,4	-1,8	
	yes	Count	44_a	51_b	95
		Expected Count	60,8	34,2	95,0
		% within pelvic_awareness	46,3%	53,7%	100,0%
		% within symptoms_life	21,2%	43,6%	29,2%
		% of Total	13,5%	15,7%	29,2%
		Standardized Residual	-2,2	2,9	
Total		Count	208	117	325
		Expected Count	208,0	117,0	325,0
		% within pelvic_awareness	64,0%	36,0%	100,0%
		% within symptoms_life	100,0%	100,0%	100,0%
		% of Total	64,0%	36,0%	100,0%

Each subscript letter denotes a subset of symptoms_life categories whose column proportions do not differ significantly from each other at the ,05 level.

Chi-Square Tests

	Value	df	Asymptotic Significance (2-sided)	Exact Sig. (2-sided)	Exact Sig. (1-sided)
Pearson Chi-Square	18,221[a]	1	<,001		
Continuity Correction[b]	17,152	1	<,001		
Likelihood Ratio	17,814	1	<,001		
Fisher's Exact Test				<,001	<,001
Linear-by-Linear Association	18,165	1	<,001		
N of Valid Cases	325				

a. 0 cells (0,0%) have expected count less than 5. The minimum expected count is 34,20.
b. Computed only for a 2x2 table

Symmetric Measures

		Value	Approximate Significance
Nominal by Nominal	Phi	,237	<,001
	Cramer's V	,237	<,001
	Contingency Coefficient	,230	<,001
N of Valid Cases		325	

pfd_awareness * symptoms_life

Crosstab

			symptoms_life		
			no	1	Total
pfd_awareness	no	Count	176$_a$	80$_b$	256
		Expected Count	163,8	92,2	256,0
		% within pfd_awareness	68,8%	31,3%	100,0%
		% within symptoms_life	84,6%	68,4%	78,8%
		% of Total	54,2%	24,6%	78,8%
		Standardized Residual	,9	-1,3	
	yes	Count	32$_a$	37$_b$	69
		Expected Count	44,2	24,8	69,0
		% within pfd_awareness	46,4%	53,6%	100,0%
		% within symptoms_life	15,4%	31,6%	21,2%
		% of Total	9,8%	11,4%	21,2%
		Standardized Residual	-1,8	2,4	
Total		Count	208	117	325
		Expected Count	208,0	117,0	325,0
		% within pfd_awareness	64,0%	36,0%	100,0%
		% within symptoms_life	100,0%	100,0%	100,0%
		% of Total	64,0%	36,0%	100,0%

Each subscript letter denotes a subset of symptoms_life categories whose column proportions do not differ significantly from each other at the ,05 level.

Chi-Square Tests

	Value	df	Asymptotic Significance (2-sided)	Exact Sig. (2-sided)	Exact Sig. (1-sided)
Pearson Chi-Square	11,808[a]	1	<,001		
Continuity Correction[b]	10,857	1	<,001		
Likelihood Ratio	11,434	1	<,001		
Fisher's Exact Test				,001	<,001
Linear-by-Linear Association	11,772	1	<,001		
N of Valid Cases	325				

a. 0 cells (0,0%) have expected count less than 5. The minimum expected count is 24,84.
b. Computed only for a 2x2 table

Symmetric Measures

		Value	Approximate Significance
Nominal by Nominal	Phi	,191	<,001
	Cramer's V	,191	<,001
	Contingency Coefficient	,187	<,001
N of Valid Cases		325	

difficult_urination * symptoms_life

Crosstab

			symptoms_life		
			no	1	Total
difficult_urination	no	Count	195[a]	103[a]	298
		Expected Count	190,7	107,3	298,0
		% within difficult_urination	65,4%	34,6%	100,0%
		% within symptoms_life	93,8%	88,0%	91,7%
		% of Total	60,0%	31,7%	91,7%
		Standardized Residual	,3	-,4	
	yes	Count	13[a]	14[a]	27
		Expected Count	17,3	9,7	27,0
		% within difficult_urination	48,1%	51,9%	100,0%
		% within symptoms_life	6,3%	12,0%	8,3%
		% of Total	4,0%	4,3%	8,3%
		Standardized Residual	-1,0	1,4	
Total		Count	208	117	325
		Expected Count	208,0	117,0	325,0
		% within difficult_urination	64,0%	36,0%	100,0%
		% within symptoms_life	100,0%	100,0%	100,0%
		% of Total	64,0%	36,0%	100,0%

Each subscript letter denotes a subset of symptoms_life categories whose column proportions do not differ significantly from each other at the ,05 level.

Chi-Square Tests

	Value	df	Asymptotic Significance (2-sided)	Exact Sig. (2-sided)	Exact Sig. (1-sided)
Pearson Chi-Square	3,212[a]	1	,073		
Continuity Correction[b]	2,505	1	,113		
Likelihood Ratio	3,085	1	,079		
Fisher's Exact Test				,093	,059
Linear-by-Linear Association	3,202	1	,074		
N of Valid Cases	325				

a. 0 cells (0,0%) have expected count less than 5. The minimum expected count is 9,72.
b. Computed only for a 2x2 table

Symmetric Measures

		Value	Approximate Significance
Nominal by Nominal	Phi	,099	,073
	Cramer's V	,099	,073
	Contingency Coefficient	,099	,073
N of Valid Cases		325	

push_bowel * symptoms_life

Crosstab

			symptoms_life		
			no	1	Total
push_bowel	no	Count	196_a	92_b	288
		Expected Count	184,3	103,7	288,0
		% within push_bowel	68,1%	31,9%	100,0%
		% within symptoms_life	94,2%	78,6%	88,6%
		% of Total	60,3%	28,3%	88,6%
		Standardized Residual	,9	-1,1	
	yes	Count	12_a	25_b	37
		Expected Count	23,7	13,3	37,0
		% within push_bowel	32,4%	67,6%	100,0%
		% within symptoms_life	5,8%	21,4%	11,4%
		% of Total	3,7%	7,7%	11,4%
		Standardized Residual	-2,4	3,2	
Total		Count	208	117	325
		Expected Count	208,0	117,0	325,0
		% within push_bowel	64,0%	36,0%	100,0%
		% within symptoms_life	100,0%	100,0%	100,0%
		% of Total	64,0%	36,0%	100,0%

Each subscript letter denotes a subset of symptoms_life categories whose column proportions do not differ significantly from each other at the ,05 level.

Chi-Square Tests

	Value	df	Asymptotic Significance (2-sided)	Exact Sig. (2-sided)	Exact Sig. (1-sided)
Pearson Chi-Square	18,059[a]	1	<,001		
Continuity Correction[b]	16,546	1	<,001		
Likelihood Ratio	17,260	1	<,001		
Fisher's Exact Test				<,001	<,001
Linear-by-Linear Association	18,003	1	<,001		
N of Valid Cases	325				

a. 0 cells (0,0%) have expected count less than 5. The minimum expected count is 13,32.
b. Computed only for a 2x2 table

Symmetric Measures

		Value	Approximate Significance
Nominal by Nominal	Phi	,236	<,001
	Cramer's V	,236	<,001
	Contingency Coefficient	,229	<,001
N of Valid Cases		325	

toilet_before * symptoms_life

Crosstab

			symptoms_life		
			no	1	Total
toilet_before	no	Count	66_a	23_b	89
		Expected Count	57,0	32,0	89,0
		% within toilet_before	74,2%	25,8%	100,0%
		% within symptoms_life	31,7%	19,7%	27,4%
		% of Total	20,3%	7,1%	27,4%
		Standardized Residual	1,2	-1,6	
	yes	Count	142_a	94_b	236
		Expected Count	151,0	85,0	236,0
		% within toilet_before	60,2%	39,8%	100,0%

	% within symptoms_life	68,3%	80,3%	72,6%
	% of Total	43,7%	28,9%	72,6%
	Standardized Residual	-,7	1,0	
Total	Count	208	117	325
	Expected Count	208,0	117,0	325,0
	% within toilet_before	64,0%	36,0%	100,0%
	% within symptoms_life	100,0%	100,0%	100,0%
	% of Total	64,0%	36,0%	100,0%

Each subscript letter denotes a subset of symptoms_life categories whose column proportions do not differ significantly from each other at the ,05 level.

Chi-Square Tests

	Value	df	Asymptotic Significance (2-sided)	Exact Sig. (2-sided)	Exact Sig. (1-sided)
Pearson Chi-Square	5,488[a]	1	,019		
Continuity Correction[b]	4,898	1	,027		
Likelihood Ratio	5,677	1	,017		
Fisher's Exact Test				,020	,013
Linear-by-Linear Association	5,471	1	,019		
N of Valid Cases	325				

a. 0 cells (0,0%) have expected count less than 5. The minimum expected count is 32,04.
b. Computed only for a 2x2 table

Symmetric Measures

		Value	Approximate Significance
Nominal by Nominal	Phi	,130	,019
	Cramer's V	,130	,019
	Contingency Coefficient	,129	,019
N of Valid Cases		325	

reduce_liquid * symptoms_life

Crosstab

			symptoms_life		Total
			no	1	
reduce_liquid	no	Count	158_a	83_a	241
		Expected Count	154,2	86,8	241,0
		% within reduce_liquid	65,6%	34,4%	100,0%
		% within symptoms_life	76,0%	70,9%	74,2%
		% of Total	48,6%	25,5%	74,2%
		Standardized Residual	,3	-,4	
	yes	Count	50_a	34_a	84
		Expected Count	53,8	30,2	84,0
		% within reduce_liquid	59,5%	40,5%	100,0%
		% within symptoms_life	24,0%	29,1%	25,8%
		% of Total	15,4%	10,5%	25,8%
		Standardized Residual	-,5	,7	
Total		Count	208	117	325
		Expected Count	208,0	117,0	325,0
		% within reduce_liquid	64,0%	36,0%	100,0%
		% within symptoms_life	100,0%	100,0%	100,0%
		% of Total	64,0%	36,0%	100,0%

Each subscript letter denotes a subset of symptoms_life categories whose column proportions do not differ significantly from each other at the ,05 level.

Chi-Square Tests

	Value	df	Asymptotic Significance (2-sided)	Exact Sig. (2-sided)	Exact Sig. (1-sided)
Pearson Chi-Square	,985[a]	1	,321		
Continuity Correction[b]	,741	1	,389		
Likelihood Ratio	,975	1	,323		
Fisher's Exact Test				,356	,194
Linear-by-Linear Association	,982	1	,322		
N of Valid Cases	325				

a. 0 cells (0,0%) have expected count less than 5. The minimum expected count is 30,24.
b. Computed only for a 2x2 table

Symmetric Measures

		Value	Approximate Significance
Nominal by Nominal	Phi	,055	,321
	Cramer's V	,055	,321
	Contingency Coefficient	,055	,321
N of Valid Cases		325	

toilet_training * symptoms_life

Crosstab

			symptoms_life		Total
			no	1	
toilet_training	no	Count	147_a	61_b	208
		Expected Count	133,1	74,9	208,0
		% within toilet_training	70,7%	29,3%	100,0%
		% within symptoms_life	70,7%	52,1%	64,0%
		% of Total	45,2%	18,8%	64,0%
		Standardized Residual	1,2	-1,6	
	yes	Count	61_a	56_b	117
		Expected Count	74,9	42,1	117,0
		% within toilet_training	52,1%	47,9%	100,0%
		% within symptoms_life	29,3%	47,9%	36,0%
		% of Total	18,8%	17,2%	36,0%
		Standardized Residual	-1,6	2,1	
Total		Count	208	117	325
		Expected Count	208,0	117,0	325,0
		% within toilet_training	64,0%	36,0%	100,0%
		% within symptoms_life	100,0%	100,0%	100,0%
		% of Total	64,0%	36,0%	100,0%

Each subscript letter denotes a subset of symptoms_life categories whose column proportions do not differ significantly from each other at the ,05 level.

Chi-Square Tests

	Value	df	Asymptotic Significance (2-sided)	Exact Sig. (2-sided)	Exact Sig. (1-sided)
Pearson Chi-Square	11,167[a]	1	<,001		
Continuity Correction[b]	10,377	1	,001		
Likelihood Ratio	11,037	1	<,001		
Fisher's Exact Test				,001	<,001
Linear-by-Linear Association	11,132	1	<,001		
N of Valid Cases	325				

a. 0 cells (0,0%) have expected count less than 5. The minimum expected count is 42,12.
b. Computed only for a 2x2 table

Symmetric Measures

		Value	Approximate Significance
Nominal by Nominal	Phi	,185	<,001
	Cramer's V	,185	<,001
	Contingency Coefficient	,182	<,001
N of Valid Cases		325	

toilet_competition * symptoms_life

Crosstab

			symptoms_life		Total
			no	1	
toilet_competition	no	Count	129_{a}	64_{a}	193
		Expected Count	123,5	69,5	193,0
		% within toilet_competition	66,8%	33,2%	100,0%
		% within symptoms_life	62,0%	54,7%	59,4%
		% of Total	39,7%	19,7%	59,4%
		Standardized Residual	,5	-,7	
	yes	Count	79_{a}	53_{a}	132
		Expected Count	84,5	47,5	132,0
		% within toilet_competition	59,8%	40,2%	100,0%
		% within symptoms_life	38,0%	45,3%	40,6%
		% of Total	24,3%	16,3%	40,6%
		Standardized Residual	-,6	,8	
Total		Count	208	117	325
		Expected Count	208,0	117,0	325,0
		% within toilet_competition	64,0%	36,0%	100,0%
		% within symptoms_life	100,0%	100,0%	100,0%
		% of Total	64,0%	36,0%	100,0%

Each subscript letter denotes a subset of symptoms_life categories whose column proportions do not differ significantly from each other at the ,05 level.

Chi-Square Tests

	Value	df	Asymptotic Significance (2-sided)	Exact Sig. (2-sided)	Exact Sig. (1-sided)
Pearson Chi-Square	1,663[a]	1	,197		
Continuity Correction[b]	1,373	1	,241		
Likelihood Ratio	1,656	1	,198		
Fisher's Exact Test				,239	,121
Linear-by-Linear Association	1,658	1	,198		
N of Valid Cases	325				

a. 0 cells (0,0%) have expected count less than 5. The minimum expected count is 47,52.

b. Computed only for a 2x2 table

Symmetric Measures

		Value	Approximate Significance
Nominal by Nominal	Phi	,072	,197
	Cramer's V	,072	,197
	Contingency Coefficient	,071	,197
N of Valid Cases		325	

caffeine * symptoms_life

Crosstab

			symptoms_life		Total
			no	1	
caffeine	no	Count	147_a	70_b	217
		Expected Count	138,9	78,1	217,0
		% within caffeine	67,7%	32,3%	100,0%
		% within symptoms_life	70,7%	59,8%	66,8%
		% of Total	45,2%	21,5%	66,8%
		Standardized Residual	,7	-,9	
	yes	Count	61_a	47_b	108
		Expected Count	69,1	38,9	108,0
		% within caffeine	56,5%	43,5%	100,0%
		% within symptoms_life	29,3%	40,2%	33,2%
		% of Total	18,8%	14,5%	33,2%
		Standardized Residual	-1,0	1,3	
Total		Count	208	117	325
		Expected Count	208,0	117,0	325,0
		% within caffeine	64,0%	36,0%	100,0%
		% within symptoms_life	100,0%	100,0%	100,0%
		% of Total	64,0%	36,0%	100,0%

Each subscript letter denotes a subset of symptoms_life categories whose column proportions do not differ significantly from each other at the ,05 level.

Chi-Square Tests

	Value	df	Asymptotic Significance (2-sided)	Exact Sig. (2-sided)	Exact Sig. (1-sided)
Pearson Chi-Square	3,969[a]	1	,046		
Continuity Correction[b]	3,495	1	,062		
Likelihood Ratio	3,923	1	,048		
Fisher's Exact Test				,050	,031
Linear-by-Linear Association	3,956	1	,047		
N of Valid Cases	325				

a. 0 cells (0,0%) have expected count less than 5. The minimum expected count is 38,88.
b. Computed only for a 2x2 table

Symmetric Measures

		Value	Approximate Significance
Nominal by Nominal	Phi	,111	,046
	Cramer's V	,111	,046
	Contingency Coefficient	,110	,046
N of Valid Cases		325	

event=combined events * symptoms_life

Crosstab

			symptoms_life		Total
			no	1	
event=combined events	others	Count	205_a	113_a	318
		Expected Count	203,5	114,5	318,0
		% within event=combined events	64,5%	35,5%	100,0%
		% within symptoms_life	98,6%	96,6%	97,8%
		% of Total	63,1%	34,8%	97,8%
		Standardized Residual	,1	-,1	
	combined	Count	3_a	4_a	7
		Expected Count	4,5	2,5	7,0
		% within event=combined events	42,9%	57,1%	100,0%
		% within symptoms_life	1,4%	3,4%	2,2%

		% of Total	0,9%	1,2%	2,2%
		Standardized Residual	-,7	,9	
Total		Count	208	117	325
		Expected Count	208,0	117,0	325,0
		% within event=combined events	64,0%	36,0%	100,0%
		% within symptoms_life	100,0%	100,0%	100,0%
		% of Total	64,0%	36,0%	100,0%

Each subscript letter denotes a subset of symptoms_life categories whose column proportions do not differ significantly from each other at the ,05 level.

Chi-Square Tests

	Value	df	Asymptotic Significance (2-sided)	Exact Sig. (2-sided)	Exact Sig. (1-sided)
Pearson Chi-Square	1,388[a]	1	,239		
Continuity Correction[b]	,609	1	,435		
Likelihood Ratio	1,320	1	,251		
Fisher's Exact Test				,257	,214
Linear-by-Linear Association	1,384	1	,239		
N of Valid Cases	325				

a. 2 cells (50,0%) have expected count less than 5. The minimum expected count is 2,52.
b. Computed only for a 2x2 table

Symmetric Measures

		Value	Approximate Significance
Nominal by Nominal	Phi	,065	,239
	Cramer's V	,065	,239
	Contingency Coefficient	,065	,239
N of Valid Cases		325	

event=hurdles * symptoms_life

Crosstab

			symptoms_life		Total
			no	1	
event=hurdles	others	Count	188_{a}	98_{a}	286
		Expected Count	183,0	103,0	286,0
		% within event=hurdles	65,7%	34,3%	100,0%
		% within symptoms_life	90,4%	83,8%	88,0%
		% of Total	57,8%	30,2%	88,0%
		Standardized Residual	,4	-,5	
	hurdles	Count	20_{a}	19_{a}	39
		Expected Count	25,0	14,0	39,0
		% within event=hurdles	51,3%	48,7%	100,0%
		% within symptoms_life	9,6%	16,2%	12,0%
		% of Total	6,2%	5,8%	12,0%
		Standardized Residual	-1,0	1,3	
Total		Count	208	117	325
		Expected Count	208,0	117,0	325,0
		% within event=hurdles	64,0%	36,0%	100,0%
		% within symptoms_life	100,0%	100,0%	100,0%
		% of Total	64,0%	36,0%	100,0%

Each subscript letter denotes a subset of symptoms_life categories whose column proportions do not differ significantly from each other at the ,05 level.

Chi-Square Tests

	Value	df	Asymptotic Significance (2-sided)	Exact Sig. (2-sided)	Exact Sig. (1-sided)
Pearson Chi-Square	3,111[a]	1	,078		
Continuity Correction[b]	2,516	1	,113		
Likelihood Ratio	3,010	1	,083		
Fisher's Exact Test				,108	,058
Linear-by-Linear Association	3,102	1	,078		
N of Valid Cases	325				

a. 0 cells (0,0%) have expected count less than 5. The minimum expected count is 14,04.
b. Computed only for a 2x2 table

Symmetric Measures

		Value	Approximate Significance
Nominal by Nominal	Phi	,098	,078
	Cramer's V	,098	,078
	Contingency Coefficient	,097	,078
N of Valid Cases		325	

event=jumps * symptoms_life

Crosstab

			symptoms_life no	symptoms_life 1	Total
event=jumps	others	Count	184_a	103_a	287
		Expected Count	183,7	103,3	287,0
		% within event=jumps	64,1%	35,9%	100,0%
		% within symptoms_life	88,5%	88,0%	88,3%
		% of Total	56,6%	31,7%	88,3%
		Standardized Residual	,0	,0	
	jumps	Count	24_a	14_a	38
		Expected Count	24,3	13,7	38,0
		% within event=jumps	63,2%	36,8%	100,0%
		% within symptoms_life	11,5%	12,0%	11,7%
		% of Total	7,4%	4,3%	11,7%
		Standardized Residual	-,1	,1	
Total		Count	208	117	325
		Expected Count	208,0	117,0	325,0
		% within event=jumps	64,0%	36,0%	100,0%
		% within symptoms_life	100,0%	100,0%	100,0%
		% of Total	64,0%	36,0%	100,0%

Each subscript letter denotes a subset of symptoms_life categories whose column proportions do not differ significantly from each other at the ,05 level.

Chi-Square Tests

	Value	df	Asymptotic Significance (2-sided)	Exact Sig. (2-sided)	Exact Sig. (1-sided)
Pearson Chi-Square	,013[a]	1	,908		
Continuity Correction[b]	,000	1	1,000		
Likelihood Ratio	,013	1	,909		
Fisher's Exact Test				1,000	,521
Linear-by-Linear Association	,013	1	,909		
N of Valid Cases	325				

a. 0 cells (0,0%) have expected count less than 5. The minimum expected count is 13,68.
b. Computed only for a 2x2 table

Symmetric Measures

		Value	Approximate Significance
Nominal by Nominal	Phi	,006	,908
	Cramer's V	,006	,908
	Contingency Coefficient	,006	,908
N of Valid Cases		325	

event=long distance runs * symptoms_life

Crosstab

			symptoms_life no	symptoms_life 1
event=long distance runs	others	Count	202_a	104_b
		Expected Count	195,8	110,2
		% within event=long distance runs	66,0%	34,0%
		% within symptoms_life	97,1%	88,9%
		% of Total	62,2%	32,0%
		Standardized Residual	,4	-,6
	long distance	Count	6_a	13_b
		Expected Count	12,2	6,8
		% within event=long distance runs	31,6%	68,4%
		% within symptoms_life	2,9%	11,1%
		% of Total	1,8%	4,0%
		Standardized Residual	-1,8	2,4
Total		Count	208	117
		Expected Count	208,0	117,0
		% within event=long distance runs	64,0%	36,0%
		% within symptoms_life	100,0%	100,0%
		% of Total	64,0%	36,0%

Crosstab

			Total
event=long distance runs	others	Count	306
		Expected Count	306,0
		% within event=long distance runs	100,0%
		% within symptoms_life	94,2%
		% of Total	94,2%
		Standardized Residual	
	long distance	Count	19
		Expected Count	19,0
		% within event=long distance runs	100,0%
		% within symptoms_life	5,8%
		% of Total	5,8%
		Standardized Residual	
Total		Count	325
		Expected Count	325,0
		% within event=long distance runs	100,0%
		% within symptoms_life	100,0%
		% of Total	100,0%

Each subscript letter denotes a subset of symptoms_life categories whose column proportions do not differ significantly from each other at the ,05 level.

Chi-Square Tests

	Value	df	Asymptotic Significance (2-sided)	Exact Sig. (2-sided)	Exact Sig. (1-sided)
Pearson Chi-Square	9,206[a]	1	,002		
Continuity Correction[b]	7,772	1	,005		
Likelihood Ratio	8,762	1	,003		
Fisher's Exact Test				,005	,003
Linear-by-Linear Association	9,178	1	,002		
N of Valid Cases	325				

a. 0 cells (0,0%) have expected count less than 5. The minimum expected count is 6,84.
b. Computed only for a 2x2 table

Symmetric Measures

		Value	Approximate Significance
Nominal by Nominal	Phi	,168	,002
	Cramer's V	,168	,002
	Contingency Coefficient	,166	,002
N of Valid Cases		325	

event=middle distance runs * symptoms_life

Crosstab

			symptoms_life	
			no	1
event=middle distance runs	others	Count	183_a	103_a
		Expected Count	183,0	103,0
		% within event=middle distance runs	64,0%	36,0%
		% within symptoms_life	88,0%	88,0%
		% of Total	56,3%	31,7%
		Standardized Residual	,0	,0
	middle distance	Count	25_a	14_a
		Expected Count	25,0	14,0
		% within event=middle distance runs	64,1%	35,9%
		% within symptoms_life	12,0%	12,0%
		% of Total	7,7%	4,3%
		Standardized Residual	,0	,0
Total		Count	208	117
		Expected Count	208,0	117,0
		% within event=middle distance runs	64,0%	36,0%
		% within symptoms_life	100,0%	100,0%
		% of Total	64,0%	36,0%

Crosstab

			Total
event=middle distance runs	others	Count	286
		Expected Count	286,0
		% within event=middle distance runs	100,0%
		% within symptoms_life	88,0%
		% of Total	88,0%
		Standardized Residual	
	middle distance	Count	39
		Expected Count	39,0
		% within event=middle distance runs	100,0%
		% within symptoms_life	12,0%
		% of Total	12,0%
		Standardized Residual	

Total	Count	325
	Expected Count	325,0
	% within event=middle distance runs	100,0%
	% within symptoms_life	100,0%
	% of Total	100,0%

Each subscript letter denotes a subset of symptoms_life categories whose column proportions do not differ significantly from each other at the ,05 level.

Chi-Square Tests

	Value	df	Asymptotic Significance (2-sided)	Exact Sig. (2-sided)	Exact Sig. (1-sided)
Pearson Chi-Square	,000[a]	1	,989		
Continuity Correction[b]	,000	1	1,000		
Likelihood Ratio	,000	1	,989		
Fisher's Exact Test				1,000	,570
Linear-by-Linear Association	,000	1	,989		
N of Valid Cases	325				

a. 0 cells (0,0%) have expected count less than 5. The minimum expected count is 14,04.
b. Computed only for a 2x2 table

Symmetric Measures

		Value	Approximate Significance
Nominal by Nominal	Phi	-,001	,989
	Cramer's V	,001	,989
	Contingency Coefficient	,001	,989
N of Valid Cases		325	

event=race walking * symptoms_life

Crosstab

			symptoms_life no	symptoms_life 1	Total
event=race walking	others	Count	188_a	111_a	299
		Expected Count	191,4	107,6	299,0
		% within event=race walking	62,9%	37,1%	100,0%
		% within symptoms_life	90,4%	94,9%	92,0%
		% of Total	57,8%	34,2%	92,0%
		Standardized Residual	-,2	,3	
	race walking	Count	20_a	6_a	26
		Expected Count	16,6	9,4	26,0
		% within event=race walking	76,9%	23,1%	100,0%
		% within symptoms_life	9,6%	5,1%	8,0%
		% of Total	6,2%	1,8%	8,0%
		Standardized Residual	,8	-1,1	
Total		Count	208	117	325
		Expected Count	208,0	117,0	325,0
		% within event=race walking	64,0%	36,0%	100,0%
		% within symptoms_life	100,0%	100,0%	100,0%
		% of Total	64,0%	36,0%	100,0%

Each subscript letter denotes a subset of symptoms_life categories whose column proportions do not differ significantly from each other at the ,05 level.

Chi-Square Tests

	Value	df	Asymptotic Significance (2-sided)	Exact Sig. (2-sided)	Exact Sig. (1-sided)
Pearson Chi-Square	2,048[a]	1	,152		
Continuity Correction[b]	1,484	1	,223		
Likelihood Ratio	2,184	1	,139		
Fisher's Exact Test				,202	,110
Linear-by-Linear Association	2,042	1	,153		
N of Valid Cases	325				

a. 0 cells (0,0%) have expected count less than 5. The minimum expected count is 9,36.
b. Computed only for a 2x2 table

Symmetric Measures

		Value	Approximate Significance
Nominal by Nominal	Phi	-,079	,152
	Cramer's V	,079	,152
	Contingency Coefficient	,079	,152
N of Valid Cases		325	

event=throws * symptoms_life

Crosstab

			symptoms_life		Total
			no	1	
event=throws	others	Count	191_a	105_a	296
		Expected Count	189,4	106,6	296,0
		% within event=throws	64,5%	35,5%	100,0%
		% within symptoms_life	91,8%	89,7%	91,1%
		% of Total	58,8%	32,3%	91,1%
		Standardized Residual	,1	-,2	
	throws	Count	17_a	12_a	29
		Expected Count	18,6	10,4	29,0
		% within event=throws	58,6%	41,4%	100,0%
		% within symptoms_life	8,2%	10,3%	8,9%
		% of Total	5,2%	3,7%	8,9%
		Standardized Residual	-,4	,5	
Total		Count	208	117	325
		Expected Count	208,0	117,0	325,0
		% within event=throws	64,0%	36,0%	100,0%
		% within symptoms_life	100,0%	100,0%	100,0%
		% of Total	64,0%	36,0%	100,0%

Each subscript letter denotes a subset of symptoms_life categories whose column proportions do not differ significantly from each other at the ,05 level.

Chi-Square Tests

	Value	df	Asymptotic Significance (2-sided)	Exact Sig. (2-sided)	Exact Sig. (1-sided)
Pearson Chi-Square	,400[a]	1	,527		
Continuity Correction[b]	,185	1	,667		
Likelihood Ratio	,393	1	,531		
Fisher's Exact Test				,547	,329
Linear-by-Linear Association	,399	1	,528		
N of Valid Cases	325				

a. 0 cells (0,0%) have expected count less than 5. The minimum expected count is 10,44.
b. Computed only for a 2x2 table

Symmetric Measures

		Value	Approximate Significance
Nominal by Nominal	Phi	,035	,527
	Cramer's V	,035	,527
	Contingency Coefficient	,035	,527
N of Valid Cases		325	

health_conditions=constipation * symptoms_life

Crosstab

			symptoms_life	
			no	1
health_conditions=constipation	others	Count	207_a	112_b
		Expected Count	204,2	114,8
		% within health_conditions=constipation	64,9%	35,1%
		% within symptoms_life	99,5%	95,7%
		% of Total	63,7%	34,5%
		Standardized Residual	,2	-,3
	constipations	Count	1_a	5_b
		Expected Count	3,8	2,2
		% within health_conditions=constipation	16,7%	83,3%
		% within symptoms_life	0,5%	4,3%
		% of Total	0,3%	1,5%
		Standardized Residual	-1,4	1,9
Total		Count	208	117
		Expected Count	208,0	117,0
		% within health_conditions=constipation	64,0%	36,0%
		% within symptoms_life	100,0%	100,0%
		% of Total	64,0%	36,0%

Crosstab

			Total
health_conditions=constipation	others	Count	319
		Expected Count	319,0
		% within health_conditions=constipation	100,0%
		% within symptoms_life	98,2%
		% of Total	98,2%
		Standardized Residual	
	constipations	Count	6
		Expected Count	6,0
		% within health_conditions=constipation	100,0%
		% within symptoms_life	1,8%
		% of Total	1,8%
		Standardized Residual	
Total		Count	325
		Expected Count	325,0
		% within health_conditions=constipation	100,0%
		% within symptoms_life	100,0%
		% of Total	100,0%

Each subscript letter denotes a subset of symptoms_life categories whose column proportions do not differ significantly from each other at the ,05 level.

Chi-Square Tests

	Value	df	Asymptotic Significance (2-sided)	Exact Sig. (2-sided)	Exact Sig. (1-sided)
Pearson Chi-Square	5,944[a]	1	,015		
Continuity Correction[b]	4,035	1	,045		
Likelihood Ratio	5,812	1	,016		
Fisher's Exact Test				,024	,024
Linear-by-Linear Association	5,926	1	,015		
N of Valid Cases	325				

a. 2 cells (50,0%) have expected count less than 5. The minimum expected count is 2,16.
b. Computed only for a 2x2 table

Symmetric Measures

		Value	Approximate Significance
Nominal by Nominal	Phi	,135	,015
	Cramer's V	,135	,015
	Contingency Coefficient	,134	,015
N of Valid Cases		325	

health_conditions=diabetes * symptoms_life

Crosstab

			symptoms_life		
			no	1	Total
health_conditions=diabetes	others	Count	207$_a$	117$_a$	324
		Expected Count	207,4	116,6	324,0
		% within health_conditions=diabetes	63,9%	36,1%	100,0%
		% within symptoms_life	99,5%	100,0%	99,7%
		% of Total	63,7%	36,0%	99,7%
		Standardized Residual	,0	,0	
	diabetes	Count	1$_a$	0$_a$	1
		Expected Count	,6	,4	1,0
		% within health_conditions=diabetes	100,0%	0,0%	100,0%
		% within symptoms_life	0,5%	0,0%	0,3%
		% of Total	0,3%	0,0%	0,3%
		Standardized Residual	,5	-,6	
Total		Count	208	117	325
		Expected Count	208,0	117,0	325,0
		% within health_conditions=diabetes	64,0%	36,0%	100,0%
		% within symptoms_life	100,0%	100,0%	100,0%
		% of Total	64,0%	36,0%	100,0%

Each subscript letter denotes a subset of symptoms_life categories whose column proportions do not differ significantly from each other at the ,05 level.

Chi-Square Tests

	Value	df	Asymptotic Significance (2-sided)	Exact Sig. (2-sided)	Exact Sig. (1-sided)
Pearson Chi-Square	,564[a]	1	,453		
Continuity Correction[b]	,000	1	1,000		
Likelihood Ratio	,894	1	,344		
Fisher's Exact Test				1,000	,640

Linear-by-Linear Association	,563	1	,453		
N of Valid Cases	325				

a. 2 cells (50,0%) have expected count less than 5. The minimum expected count is ,36.
b. Computed only for a 2x2 table

Symmetric Measures

		Value	Approximate Significance
Nominal by Nominal	Phi	-,042	,453
	Cramer's V	,042	,453
	Contingency Coefficient	,042	,453
N of Valid Cases		325	

health_conditions=frequent UI * symptoms_life

Crosstab

			symptoms_life	
			no	1
health_conditions=frequent UI	others	Count	202a	112a
		Expected Count	201,0	113,0
		% within health_conditions=frequent UI	64,3%	35,7%
		% within symptoms_life	97,1%	95,7%
		% of Total	62,2%	34,5%
		Standardized Residual	,1	-,1
	Frequent UI	Count	6a	5a
		Expected Count	7,0	4,0
		% within health_conditions=frequent UI	54,5%	45,5%
		% within symptoms_life	2,9%	4,3%
		% of Total	1,8%	1,5%
		Standardized Residual	-,4	,5
Total		Count	208	117
		Expected Count	208,0	117,0
		% within health_conditions=frequent UI	64,0%	36,0%
		% within symptoms_life	100,0%	100,0%
		% of Total	64,0%	36,0%

Crosstab

			Total
health_conditions=frequent UI	others	Count	314
		Expected Count	314,0
		% within health_conditions=frequent UI	100,0%
		% within symptoms_life	96,6%
		% of Total	96,6%
		Standardized Residual	
	Frequent UI	Count	11
		Expected Count	11,0
		% within health_conditions=frequent UI	100,0%
		% within symptoms_life	3,4%
		% of Total	3,4%
		Standardized Residual	
Total		Count	325

	Expected Count	325,0
	% within health_conditions=frequent UI	100,0%
	% within symptoms_life	100,0%
	% of Total	100,0%

Each subscript letter denotes a subset of symptoms_life categories whose column proportions do not differ significantly from each other at the ,05 level.

Chi-Square Tests

	Value	df	Asymptotic Significance (2-sided)	Exact Sig. (2-sided)	Exact Sig. (1-sided)
Pearson Chi-Square	,442[a]	1	,506		
Continuity Correction[b]	,119	1	,730		
Likelihood Ratio	,429	1	,513		
Fisher's Exact Test				,533	,356
Linear-by-Linear Association	,440	1	,507		
N of Valid Cases	325				

a. 1 cells (25,0%) have expected count less than 5. The minimum expected count is 3,96.
b. Computed only for a 2x2 table

Symmetric Measures

		Value	Approximate Significance
Nominal by Nominal	Phi	,037	,506
	Cramer's V	,037	,506
	Contingency Coefficient	,037	,506
N of Valid Cases		325	

health_conditions=heart arrhytmia * symptoms_life

Crosstab

			symptoms_life	
			no	1
health_conditions=heart arrhytmia	others	Count	207_a	117_a
		Expected Count	207,4	116,6
		% within health_conditions=heart arrhytmia	63,9%	36,1%
		% within symptoms_life	99,5%	100,0%
		% of Total	63,7%	36,0%
		Standardized Residual	,0	,0
	heart arrhytmia	Count	1_a	0_a
		Expected Count	,6	,4
		% within health_conditions=heart arrhytmia	100,0%	0,0%
		% within symptoms_life	0,5%	0,0%
		% of Total	0,3%	0,0%
		Standardized Residual	,5	-,6
Total		Count	208	117
		Expected Count	208,0	117,0
		% within health_conditions=heart arrhytmia	64,0%	36,0%
		% within symptoms_life	100,0%	100,0%
		% of Total	64,0%	36,0%

Crosstab

			Total

health_conditions=heart arrhytmia	others	Count	324
		Expected Count	324,0
		% within health_conditions=heart arrhytmia	100,0%
		% within symptoms_life	99,7%
		% of Total	99,7%
		Standardized Residual	
	heart arrhytmia	Count	1
		Expected Count	1,0
		% within health_conditions=heart arrhytmia	100,0%
		% within symptoms_life	0,3%
		% of Total	0,3%
		Standardized Residual	
Total		Count	325
		Expected Count	325,0
		% within health_conditions=heart arrhytmia	100,0%
		% within symptoms_life	100,0%
		% of Total	100,0%

Each subscript letter denotes a subset of symptoms_life categories whose column proportions do not differ significantly from each other at the ,05 level.

Chi-Square Tests

	Value	df	Asymptotic Significance (2-sided)	Exact Sig. (2-sided)	Exact Sig. (1-sided)
Pearson Chi-Square	,564[a]	1	,453		
Continuity Correction[b]	,000	1	1,000		
Likelihood Ratio	,894	1	,344		
Fisher's Exact Test				1,000	,640
Linear-by-Linear Association	,563	1	,453		
N of Valid Cases	325				

a. 2 cells (50,0%) have expected count less than 5. The minimum expected count is ,36.
b. Computed only for a 2x2 table

Symmetric Measures

		Value	Approximate Significance
Nominal by Nominal	Phi	-,042	,453
	Cramer's V	,042	,453
	Contingency Coefficient	,042	,453
N of Valid Cases		325	

health_conditions=pelvic surgery * symptoms_life

Crosstab

			symptoms_life	
			no	1
health_conditions=pelvic surgery	others	Count	207_a	116_a
		Expected Count	206,7	116,3
		% within health_conditions=pelvic surgery	64,1%	35,9%
		% within symptoms_life	99,5%	99,1%
		% of Total	63,7%	35,7%
		Standardized Residual	,0	,0
	pelvic surgery	Count	1_a	1_a

		Expected Count	1,3	,7
		% within health_conditions=pelvic surgery	50,0%	50,0%
		% within symptoms_life	0,5%	0,9%
		% of Total	0,3%	0,3%
		Standardized Residual	-,2	,3
Total		Count	208	117
		Expected Count	208,0	117,0
		% within health_conditions=pelvic surgery	64,0%	36,0%
		% within symptoms_life	100,0%	100,0%
		% of Total	64,0%	36,0%

Crosstab

			Total
health_conditions=pelvic surgery	others	Count	323
		Expected Count	323,0
		% within health_conditions=pelvic surgery	100,0%
		% within symptoms_life	99,4%
		% of Total	99,4%
		Standardized Residual	
	pelvic surgery	Count	2
		Expected Count	2,0
		% within health_conditions=pelvic surgery	100,0%
		% within symptoms_life	0,6%
		% of Total	0,6%
		Standardized Residual	
Total		Count	325
		Expected Count	325,0
		% within health_conditions=pelvic surgery	100,0%
		% within symptoms_life	100,0%
		% of Total	100,0%

Each subscript letter denotes a subset of symptoms_life categories whose column proportions do not differ significantly from each other at the ,05 level.

Chi-Square Tests

	Value	df	Asymptotic Significance (2-sided)	Exact Sig. (2-sided)	Exact Sig. (1-sided)
Pearson Chi-Square	,171[a]	1	,679		
Continuity Correction[b]	,000	1	1,000		
Likelihood Ratio	,164	1	,685		
Fisher's Exact Test				1,000	,591
Linear-by-Linear Association	,171	1	,680		
N of Valid Cases	325				

a. 2 cells (50,0%) have expected count less than 5. The minimum expected count is ,72.
b. Computed only for a 2x2 table

Symmetric Measures

		Value	Approximate Significance
Nominal by Nominal	Phi	,023	,679
	Cramer's V	,023	,679
	Contingency Coefficient	,023	,679
N of Valid Cases		325	

health_conditions=hypertension * symptoms_life

Crosstab

			symptoms_life	
			no	1
health_conditions=hypertension	others	Count	208_a	115_a
		Expected Count	206,7	116,3
		% within health_conditions=hypertension	64,4%	35,6%
		% within symptoms_life	100,0%	98,3%
		% of Total	64,0%	35,4%
		Standardized Residual	,1	-,1
	hypertension	Count	0_a	2_a
		Expected Count	1,3	,7
		% within health_conditions=hypertension	0,0%	100,0%
		% within symptoms_life	0,0%	1,7%
		% of Total	0,0%	0,6%
		Standardized Residual	-1,1	1,5
Total		Count	208	117
		Expected Count	208,0	117,0
		% within health_conditions=hypertension	64,0%	36,0%
		% within symptoms_life	100,0%	100,0%
		% of Total	64,0%	36,0%

Crosstab

			Total
health_conditions=hypertension	others	Count	323
		Expected Count	323,0
		% within health_conditions=hypertension	100,0%
		% within symptoms_life	99,4%
		% of Total	99,4%
		Standardized Residual	
	hypertension	Count	2
		Expected Count	2,0
		% within health_conditions=hypertension	100,0%
		% within symptoms_life	0,6%
		% of Total	0,6%
		Standardized Residual	
Total		Count	325
		Expected Count	325,0
		% within health_conditions=hypertension	100,0%
		% within symptoms_life	100,0%
		% of Total	100,0%

Each subscript letter denotes a subset of symptoms_life categories whose column proportions do not differ significantly from each other at the ,05 level.

Chi-Square Tests

	Value	df	Asymptotic Significance (2-sided)	Exact Sig. (2-sided)	Exact Sig. (1-sided)
Pearson Chi-Square	3,578[a]	1	,059		
Continuity Correction[b]	1,328	1	,249		
Likelihood Ratio	4,109	1	,043		
Fisher's Exact Test				,129	,129
Linear-by-Linear Association	3,567	1	,059		
N of Valid Cases	325				

a. 2 cells (50,0%) have expected count less than 5. The minimum expected count is ,72.
b. Computed only for a 2x2 table

Symmetric Measures

		Value	Approximate Significance
Nominal by Nominal	Phi	,105	,059
	Cramer's V	,105	,059
	Contingency Coefficient	,104	,059
N of Valid Cases		325	

health_conditions=hyperthyrodism * symptoms_life

Crosstab

			symptoms_life no	symptoms_life 1
health_conditions=hyperthyrodism	others	Count	208_a	116_a
		Expected Count	207,4	116,6
		% within health_conditions=hyperthyrodism	64,2%	35,8%
		% within symptoms_life	100,0%	99,1%
		% of Total	64,0%	35,7%
		Standardized Residual	,0	-,1
	hyperthyrodism	Count	0_a	1_a
		Expected Count	,6	,4
		% within health_conditions=hyperthyrodism	0,0%	100,0%
		% within symptoms_life	0,0%	0,9%
		% of Total	0,0%	0,3%
		Standardized Residual	-,8	1,1
Total		Count	208	117
		Expected Count	208,0	117,0
		% within health_conditions=hyperthyrodism	64,0%	36,0%
		% within symptoms_life	100,0%	100,0%
		% of Total	64,0%	36,0%

Crosstab

			Total
health_conditions=hyperthyrodism	others	Count	324
		Expected Count	324,0
		% within health_conditions=hyperthyrodism	100,0%
		% within symptoms_life	99,7%
		% of Total	99,7%
		Standardized Residual	
	hyperthyrodism	Count	1
		Expected Count	1,0

		% within health_conditions=hyperthyrodism	100,0%
		% within symptoms_life	0,3%
		% of Total	0,3%
		Standardized Residual	
Total		Count	325
		Expected Count	325,0
		% within health_conditions=hyperthyrodism	100,0%
		% within symptoms_life	100,0%
		% of Total	100,0%

Each subscript letter denotes a subset of symptoms_life categories whose column proportions do not differ significantly from each other at the ,05 level.

Chi-Square Tests

	Value	df	Asymptotic Significance (2-sided)	Exact Sig. (2-sided)	Exact Sig. (1-sided)
Pearson Chi-Square	1,783[a]	1	,182		
Continuity Correction[b]	,085	1	,770		
Likelihood Ratio	2,049	1	,152		
Fisher's Exact Test				,360	,360
Linear-by-Linear Association	1,778	1	,182		
N of Valid Cases	325				

a. 2 cells (50,0%) have expected count less than 5. The minimum expected count is ,36.
b. Computed only for a 2x2 table

Symmetric Measures

		Value	Approximate Significance
Nominal by Nominal	Phi	,074	,182
	Cramer's V	,074	,182
	Contingency Coefficient	,074	,182
N of Valid Cases		325	

health_conditions=respiratory issues * symptoms_life

Crosstab

			symptoms_life	
			no	1
health_conditions=respiratory issues	others	Count	198_a	102_b
		Expected Count	192,0	108,0
		% within health_conditions=respiratory issues	66,0%	34,0%
		% within symptoms_life	95,2%	87,2%
		% of Total	60,9%	31,4%
		Standardized Residual	,4	-,6
	respiratory issues	Count	10_a	15_b
		Expected Count	16,0	9,0
		% within health_conditions=respiratory issues	40,0%	60,0%
		% within symptoms_life	4,8%	12,8%
		% of Total	3,1%	4,6%
		Standardized Residual	-1,5	2,0
Total		Count	208	117
		Expected Count	208,0	117,0
		% within health_conditions=respiratory issues	64,0%	36,0%
		% within symptoms_life	100,0%	100,0%

		% of Total	64,0%	36,0%

Crosstab

			Total
health_conditions=respiratory issues	others	Count	300
		Expected Count	300,0
		% within health_conditions=respiratory issues	100,0%
		% within symptoms_life	92,3%
		% of Total	92,3%
		Standardized Residual	
	respiratory issues	Count	25
		Expected Count	25,0
		% within health_conditions=respiratory issues	100,0%
		% within symptoms_life	7,7%
		% of Total	7,7%
		Standardized Residual	
Total		Count	325
		Expected Count	325,0
		% within health_conditions=respiratory issues	100,0%
		% within symptoms_life	100,0%
		% of Total	100,0%

Each subscript letter denotes a subset of symptoms_life categories whose column proportions do not differ significantly from each other at the ,05 level.

Chi-Square Tests

	Value	df	Asymptotic Significance (2-sided)	Exact Sig. (2-sided)	Exact Sig. (1-sided)
Pearson Chi-Square	6,771[a]	1	,009		
Continuity Correction[b]	5,689	1	,017		
Likelihood Ratio	6,450	1	,011		
Fisher's Exact Test				,015	,010
Linear-by-Linear Association	6,750	1	,009		
N of Valid Cases	325				

a. 0 cells (0,0%) have expected count less than 5. The minimum expected count is 9,00.
b. Computed only for a 2x2 table

Symmetric Measures

		Value	Approximate Significance
Nominal by Nominal	Phi	,144	,009
	Cramer's V	,144	,009
	Contingency Coefficient	,143	,009
N of Valid Cases		325	

stress_fractures=1 to 3 * symptoms_life

Crosstab

			symptoms_life		
			no	1	Total
stress_fractures=1 to 3	others	Count	172_a	93_a	265
		Expected Count	169,6	95,4	265,0
		% within stress_fractures=1 to 3	64,9%	35,1%	100,0%

		% within symptoms_life	82,7%	79,5%	81,5%
		% of Total	52,9%	28,6%	81,5%
		Standardized Residual	,2	-,2	
	1 to 3	Count	36_{a}	24_{a}	60
		Expected Count	38,4	21,6	60,0
		% within stress_fractures=1 to 3	60,0%	40,0%	100,0%
		% within symptoms_life	17,3%	20,5%	18,5%
		% of Total	11,1%	7,4%	18,5%
		Standardized Residual	-,4	,5	
Total		Count	208	117	325
		Expected Count	208,0	117,0	325,0
		% within stress_fractures=1 to 3	64,0%	36,0%	100,0%
		% within symptoms_life	100,0%	100,0%	100,0%
		% of Total	64,0%	36,0%	100,0%

Each subscript letter denotes a subset of symptoms_life categories whose column proportions do not differ significantly from each other at the ,05 level.

Chi-Square Tests

	Value	df	Asymptotic Significance (2-sided)	Exact Sig. (2-sided)	Exact Sig. (1-sided)
Pearson Chi-Square	,511[a]	1	,475		
Continuity Correction[b]	,320	1	,571		
Likelihood Ratio	,505	1	,477		
Fisher's Exact Test				,552	,284
Linear-by-Linear Association	,509	1	,475		
N of Valid Cases	325				

a. 0 cells (0,0%) have expected count less than 5. The minimum expected count is 21,60.
b. Computed only for a 2x2 table

Symmetric Measures

		Value	Approximate Significance
Nominal by Nominal	Phi	,040	,475
	Cramer's V	,040	,475
	Contingency Coefficient	,040	,475
N of Valid Cases		325	

stress_fractures=more than 3 * symptoms_life

Crosstab

			symptoms_life	
			no	1
stress_fractures=more than 3	others	Count	206_{a}	110_{b}
		Expected Count	202,2	113,8
		% within stress_fractures=more than 3	65,2%	34,8%
		% within symptoms_life	99,0%	94,0%
		% of Total	63,4%	33,8%
		Standardized Residual	,3	-,4
	more than 3	Count	2_{a}	7_{b}
		Expected Count	5,8	3,2
		% within stress_fractures=more than 3	22,2%	77,8%
		% within symptoms_life	1,0%	6,0%
		% of Total	0,6%	2,2%
		Standardized Residual	-1,6	2,1
Total		Count	208	117
		Expected Count	208,0	117,0
		% within stress_fractures=more than 3	64,0%	36,0%

	% within symptoms_life	100,0%	100,0%
	% of Total	64,0%	36,0%

Crosstab

			Total
stress_fractures=more than 3	others	Count	316
		Expected Count	316,0
		% within stress_fractures=more than 3	100,0%
		% within symptoms_life	97,2%
		% of Total	97,2%
		Standardized Residual	
	more than 3	Count	9
		Expected Count	9,0
		% within stress_fractures=more than 3	100,0%
		% within symptoms_life	2,8%
		% of Total	2,8%
		Standardized Residual	
Total		Count	325
		Expected Count	325,0
		% within stress_fractures=more than 3	100,0%
		% within symptoms_life	100,0%
		% of Total	100,0%

Each subscript letter denotes a subset of symptoms_life categories whose column proportions do not differ significantly from each other at the ,05 level.

Chi-Square Tests

	Value	df	Asymptotic Significance (2-sided)	Exact Sig. (2-sided)	Exact Sig. (1-sided)
Pearson Chi-Square	7,012[a]	1	,008		
Continuity Correction[b]	5,271	1	,022		
Likelihood Ratio	6,749	1	,009		
Fisher's Exact Test				,012	,012
Linear-by-Linear Association	6,991	1	,008		
N of Valid Cases	325				

a. 1 cells (25,0%) have expected count less than 5. The minimum expected count is 3,24.
b. Computed only for a 2x2 table

Symmetric Measures

		Value	Approximate Significance
Nominal by Nominal	Phi	,147	,008
	Cramer's V	,147	,008
	Contingency Coefficient	,145	,008
N of Valid Cases		325	

DIFFERENCES BETWEEN SYMPTOMATIC AND ASYMPTOMATIC ATHLETES IN ATHLETICS.

A. Continuous variables

Variables	Level	SYMPTOMATIC (n=111)	ASYMPTOMATIC (n = 214)	*p*-value*
BMI (kg/m^2)	Median [Min – Max]	20.3 [15 – 30.9]	20.9 [16 – 39.2]	.003
	IQR	15.9	23.2	
Training (hours/day)	Median [Min – Max]	2 [1 – 6]	2.50 [1-8]	.108
	IQR	5	7	
Training (sessions/week)	Median [Min – Max]	6 [1 – 12]	6 [1 – 13]	.631
	IQR	11	12	

[*] Mann-Whitney test (non-parametric test) was used.

B. Categorial variables

sex * symptoms_athletics

Crosstab

			symptoms_athletics no	symptoms_athletics yes	Total
sex	male	Count	101_a	32_b	133
		Expected Count	87,6	45,4	133,0
		% within sex	75,9%	24,1%	100,0%
		% within symptoms_athletics	47,2%	28,8%	40,9%
		% of Total	31,1%	9,8%	40,9%
		Standardized Residual	1,4	-2,0	
	female	Count	113_a	79_b	192
		Expected Count	126,4	65,6	192,0
		% within sex	58,9%	41,1%	100,0%
		% within symptoms_athletics	52,8%	71,2%	59,1%
		% of Total	34,8%	24,3%	59,1%
		Standardized Residual	-1,2	1,7	
Total		Count	214	111	325
		Expected Count	214,0	111,0	325,0
		% within sex	65,8%	34,2%	100,0%
		% within symptoms_athletics	100,0%	100,0%	100,0%
		% of Total	65,8%	34,2%	100,0%

Each subscript letter denotes a subset of symptoms_athletics categories whose column proportions do not differ significantly from each other at the ,05 level.

Chi-Square Tests

	Value	df	Asymptotic Significance (2-sided)	Exact Sig. (2-sided)	Exact Sig. (1-sided)
Pearson Chi-Square	10,199[a]	1	,001		
Continuity Correction[b]	9,454	1	,002		
Likelihood Ratio	10,446	1	,001		
Fisher's Exact Test				,002	<,001
Linear-by-Linear Association	10,168	1	,001		
N of Valid Cases	325				

a. 0 cells (0,0%) have expected count less than 5. The minimum expected count is 45,42.
b. Computed only for a 2x2 table

Symmetric Measures

		Value	Approximate Significance
Nominal by Nominal	Phi	,177	,001
	Cramer's V	,177	,001
	Contingency Coefficient	,174	,001
N of Valid Cases		325	

medications * symptoms_athletics

Crosstab

			symptoms_athletics		
			no	yes	Total
medications	no	Count	209_{a}	108_{a}	317
		Expected Count	208,7	108,3	317,0
		% within medications	65,9%	34,1%	100,0%
		% within symptoms_athletics	97,7%	97,3%	97,5%
		% of Total	64,3%	33,2%	97,5%
		Standardized Residual	,0	,0	
	yes	Count	5_{a}	3_{a}	8
		Expected Count	5,3	2,7	8,0
		% within medications	62,5%	37,5%	100,0%
		% within symptoms_athletics	2,3%	2,7%	2,5%
		% of Total	1,5%	0,9%	2,5%
		Standardized Residual	-,1	,2	
Total		Count	214	111	325
		Expected Count	214,0	111,0	325,0
		% within medications	65,8%	34,2%	100,0%
		% within symptoms_athletics	100,0%	100,0%	100,0%
		% of Total	65,8%	34,2%	100,0%

Each subscript letter denotes a subset of symptoms_athletics categories whose column proportions do not differ significantly from each other at the ,05 level.

Chi-Square Tests

	Value	df	Asymptotic Significance (2-sided)	Exact Sig. (2-sided)	Exact Sig. (1-sided)
Pearson Chi-Square	,041[a]	1	,840		
Continuity Correction[b]	,000	1	1,000		
Likelihood Ratio	,040	1	,841		
Fisher's Exact Test				1,000	,553
Linear-by-Linear Association	,041	1	,840		
N of Valid Cases	325				

a. 1 cells (25,0%) have expected count less than 5. The minimum expected count is 2,73.
b. Computed only for a 2x2 table

Symmetric Measures

		Value	Approximate Significance
Nominal by Nominal	Phi	,011	,840
	Cramer's V	,011	,840
	Contingency Coefficient	,011	,840
N of Valid Cases		325	

smoking * symptoms_athletics

Crosstab

			symptoms_athletics		
			no	yes	Total
smoking	no	Count	212_a	106_b	318
		Expected Count	209,4	108,6	318,0
		% within smoking	66,7%	33,3%	100,0%
		% within symptoms_athletics	99,1%	95,5%	97,8%
		% of Total	65,2%	32,6%	97,8%
		Standardized Residual	,2	-,3	
	yes	Count	2_a	5_b	7
		Expected Count	4,6	2,4	7,0
		% within smoking	28,6%	71,4%	100,0%
		% within symptoms_athletics	0,9%	4,5%	2,2%
		% of Total	0,6%	1,5%	2,2%
		Standardized Residual	-1,2	1,7	
Total		Count	214	111	325
		Expected Count	214,0	111,0	325,0
		% within smoking	65,8%	34,2%	100,0%
		% within symptoms_athletics	100,0%	100,0%	100,0%
		% of Total	65,8%	34,2%	100,0%

Each subscript letter denotes a subset of symptoms_athletics categories whose column proportions do not differ significantly from each other at the ,05 level.

Chi-Square Tests

	Value	df	Asymptotic Significance (2-sided)	Exact Sig. (2-sided)	Exact Sig. (1-sided)
Pearson Chi-Square	4,420[a]	1	,036		
Continuity Correction[b]	2,888	1	,089		
Likelihood Ratio	4,134	1	,042		
Fisher's Exact Test				,048	,048
Linear-by-Linear Association	4,406	1	,036		
N of Valid Cases	325				

a. 2 cells (50,0%) have expected count less than 5. The minimum expected count is 2,39.
b. Computed only for a 2x2 table

Symmetric Measures

		Value	Approximate Significance
Nominal by Nominal	Phi	,117	,036
	Cramer's V	,117	,036
	Contingency Coefficient	,116	,036
N of Valid Cases		325	

pelvic_injury * symptoms_athletics

Crosstab

			symptoms_athletics		
			no	yes	Total
pelvic_injury	no	Count	195_a	93_b	288
		Expected Count	189,6	98,4	288,0
		% within pelvic_injury	67,7%	32,3%	100,0%
		% within symptoms_athletics	91,1%	83,8%	88,6%
		% of Total	60,0%	28,6%	88,6%
		Standardized Residual	,4	-,5	
	yes	Count	19_a	18_b	37
		Expected Count	24,4	12,6	37,0
		% within pelvic_injury	51,4%	48,6%	100,0%
		% within symptoms_athletics	8,9%	16,2%	11,4%
		% of Total	5,8%	5,5%	11,4%
		Standardized Residual	-1,1	1,5	
Total		Count	214	111	325
		Expected Count	214,0	111,0	325,0
		% within pelvic_injury	65,8%	34,2%	100,0%
		% within symptoms_athletics	100,0%	100,0%	100,0%
		% of Total	65,8%	34,2%	100,0%

Each subscript letter denotes a subset of symptoms_athletics categories whose column proportions do not differ significantly from each other at the ,05 level.

Chi-Square Tests

	Value	df	Asymptotic Significance (2-sided)	Exact Sig. (2-sided)	Exact Sig. (1-sided)
Pearson Chi-Square	3,901[a]	1	,048		
Continuity Correction[b]	3,207	1	,073		
Likelihood Ratio	3,735	1	,053		
Fisher's Exact Test				,064	,039
Linear-by-Linear Association	3,889	1	,049		
N of Valid Cases	325				

a. 0 cells (0,0%) have expected count less than 5. The minimum expected count is 12,64.
b. Computed only for a 2x2 table

Symmetric Measures

		Value	Approximate Significance
Nominal by Nominal	Phi	,110	,048
	Cramer's V	,110	,048
	Contingency Coefficient	,109	,048
N of Valid Cases		325	

other_sports * symptoms_athletics

Crosstab

			symptoms_athletics		
			no	yes	Total
other_sports	no	Count	166_a	79_a	245
		Expected Count	161,3	83,7	245,0
		% within other_sports	67,8%	32,2%	100,0%
		% within symptoms_athletics	77,6%	71,2%	75,4%
		% of Total	51,1%	24,3%	75,4%
		Standardized Residual	,4	-,5	
	yes	Count	48_a	32_a	80
		Expected Count	52,7	27,3	80,0
		% within other_sports	60,0%	40,0%	100,0%

	% within symptoms_athletics	22,4%	28,8%	24,6%
	% of Total	14,8%	9,8%	24,6%
	Standardized Residual	-,6	,9	
Total	Count	214	111	325
	Expected Count	214,0	111,0	325,0
	% within other_sports	65,8%	34,2%	100,0%
	% within symptoms_athletics	100,0%	100,0%	100,0%
	% of Total	65,8%	34,2%	100,0%

Each subscript letter denotes a subset of symptoms_athletics categories whose column proportions do not differ significantly from each other at the ,05 level.

Chi-Square Tests

	Value	df	Asymptotic Significance (2-sided)	Exact Sig. (2-sided)	Exact Sig. (1-sided)
Pearson Chi-Square	1,613[a]	1	,204		
Continuity Correction[b]	1,286	1	,257		
Likelihood Ratio	1,587	1	,208		
Fisher's Exact Test				,223	,129
Linear-by-Linear Association	1,608	1	,205		
N of Valid Cases	325				

a. 0 cells (0,0%) have expected count less than 5. The minimum expected count is 27,32.
b. Computed only for a 2x2 table

Symmetric Measures

		Value	Approximate Significance
Nominal by Nominal	Phi	,070	,204
	Cramer's V	,070	,204
	Contingency Coefficient	,070	,204
N of Valid Cases		325	

pelvic_awareness * symptoms_athletics

Crosstab

			symptoms_athletics		
			no	yes	Total
pelvic_awareness	no	Count	161_a	69_b	230
		Expected Count	151,4	78,6	230,0
		% within pelvic_awareness	70,0%	30,0%	100,0%
		% within symptoms_athletics	75,2%	62,2%	70,8%
		% of Total	49,5%	21,2%	70,8%
		Standardized Residual	,8	-1,1	
	yes	Count	53_a	42_b	95
		Expected Count	62,6	32,4	95,0
		% within pelvic_awareness	55,8%	44,2%	100,0%
		% within symptoms_athletics	24,8%	37,8%	29,2%
		% of Total	16,3%	12,9%	29,2%
		Standardized Residual	-1,2	1,7	
Total		Count	214	111	325
		Expected Count	214,0	111,0	325,0
		% within pelvic_awareness	65,8%	34,2%	100,0%
		% within symptoms_athletics	100,0%	100,0%	100,0%
		% of Total	65,8%	34,2%	100,0%

Each subscript letter denotes a subset of symptoms_athletics categories whose column proportions do not differ significantly from each other at the ,05 level.

Chi-Square Tests

	Value	df	Asymptotic Significance (2-sided)	Exact Sig. (2-sided)	Exact Sig. (1-sided)
Pearson Chi-Square	6,037[a]	1	,014		
Continuity Correction[b]	5,422	1	,020		
Likelihood Ratio	5,914	1	,015		
Fisher's Exact Test				,020	,010
Linear-by-Linear Association	6,018	1	,014		
N of Valid Cases	325				

a. 0 cells (0,0%) have expected count less than 5. The minimum expected count is 32,45.
b. Computed only for a 2x2 table

Symmetric Measures

		Value	Approximate Significance
Nominal by Nominal	Phi	,136	,014
	Cramer's V	,136	,014
	Contingency Coefficient	,135	,014
N of Valid Cases		325	

pfd_awareness * symptoms_athletics

Crosstab

			symptoms_athletics		Total
			no	yes	
pfd_awareness	no	Count	176$_a$	80$_b$	256
		Expected Count	168,6	87,4	256,0
		% within pfd_awareness	68,8%	31,3%	100,0%
		% within symptoms_athletics	82,2%	72,1%	78,8%
		% of Total	54,2%	24,6%	78,8%
		Standardized Residual	,6	-,8	
	yes	Count	38$_a$	31$_b$	69
		Expected Count	45,4	23,6	69,0
		% within pfd_awareness	55,1%	44,9%	100,0%
		% within symptoms_athletics	17,8%	27,9%	21,2%
		% of Total	11,7%	9,5%	21,2%
		Standardized Residual	-1,1	1,5	
Total		Count	214	111	325
		Expected Count	214,0	111,0	325,0
		% within pfd_awareness	65,8%	34,2%	100,0%
		% within symptoms_athletics	100,0%	100,0%	100,0%
		% of Total	65,8%	34,2%	100,0%

Each subscript letter denotes a subset of symptoms_athletics categories whose column proportions do not differ significantly from each other at the ,05 level.

Chi-Square Tests

	Value	df	Asymptotic Significance (2-sided)	Exact Sig. (2-sided)	Exact Sig. (1-sided)
Pearson Chi-Square	4,521[a]	1	,033		
Continuity Correction[b]	3,933	1	,047		
Likelihood Ratio	4,394	1	,036		
Fisher's Exact Test				,045	,025
Linear-by-Linear Association	4,507	1	,034		
N of Valid Cases	325				

a. 0 cells (0,0%) have expected count less than 5. The minimum expected count is 23,57.
b. Computed only for a 2x2 table

Symmetric Measures

		Value	Approximate Significance
Nominal by Nominal	Phi	,118	,033
	Cramer's V	,118	,033
	Contingency Coefficient	,117	,033
N of Valid Cases		325	

difficult_urination * symptoms_athletics

Crosstab

			symptoms_athletics		
			no	yes	Total
difficult_urination	no	Count	202_a	96_b	298
		Expected Count	196,2	101,8	298,0
		% within difficult_urination	67,8%	32,2%	100,0%
		% within symptoms_athletics	94,4%	86,5%	91,7%
		% of Total	62,2%	29,5%	91,7%
		Standardized Residual	,4	-,6	
	yes	Count	12_a	15_b	27
		Expected Count	17,8	9,2	27,0
		% within difficult_urination	44,4%	55,6%	100,0%
		% within symptoms_athletics	5,6%	13,5%	8,3%
		% of Total	3,7%	4,6%	8,3%
		Standardized Residual	-1,4	1,9	
Total		Count	214	111	325
		Expected Count	214,0	111,0	325,0
		% within difficult_urination	65,8%	34,2%	100,0%
		% within symptoms_athletics	100,0%	100,0%	100,0%
		% of Total	65,8%	34,2%	100,0%

Each subscript letter denotes a subset of symptoms_athletics categories whose column proportions do not differ significantly from each other at the ,05 level.

Chi-Square Tests

	Value	df	Asymptotic Significance (2-sided)	Exact Sig. (2-sided)	Exact Sig. (1-sided)
Pearson Chi-Square	5,997[a]	1	,014		
Continuity Correction[b]	5,004	1	,025		
Likelihood Ratio	5,664	1	,017		
Fisher's Exact Test				,019	,014
Linear-by-Linear Association	5,979	1	,014		
N of Valid Cases	325				

a. 0 cells (0,0%) have expected count less than 5. The minimum expected count is 9,22.
b. Computed only for a 2x2 table

Symmetric Measures

		Value	Approximate Significance
Nominal by Nominal	Phi	,136	,014
	Cramer's V	,136	,014
	Contingency Coefficient	,135	,014
N of Valid Cases		325	

push_bowel * symptoms_athletics

Crosstab

			symptoms_athletics		
			no	yes	Total
push_bowel	no	Count	201a	87b	288
		Expected Count	189,6	98,4	288,0
		% within push_bowel	69,8%	30,2%	100,0%
		% within symptoms_athletics	93,9%	78,4%	88,6%
		% of Total	61,8%	26,8%	88,6%
		Standardized Residual	,8	-1,1	
	yes	Count	13a	24b	37
		Expected Count	24,4	12,6	37,0
		% within push_bowel	35,1%	64,9%	100,0%
		% within symptoms_athletics	6,1%	21,6%	11,4%
		% of Total	4,0%	7,4%	11,4%
		Standardized Residual	-2,3	3,2	
Total		Count	214	111	325
		Expected Count	214,0	111,0	325,0
		% within push_bowel	65,8%	34,2%	100,0%
		% within symptoms_athletics	100,0%	100,0%	100,0%
		% of Total	65,8%	34,2%	100,0%

Each subscript letter denotes a subset of symptoms_athletics categories whose column proportions do not differ significantly from each other at the ,05 level.

Chi-Square Tests

	Value	df	Asymptotic Significance (2-sided)	Exact Sig. (2-sided)	Exact Sig. (1-sided)
Pearson Chi-Square	17,511[a]	1	<,001		
Continuity Correction[b]	16,004	1	<,001		
Likelihood Ratio	16,492	1	<,001		
Fisher's Exact Test				<,001	<,001
Linear-by-Linear Association	17,457	1	<,001		
N of Valid Cases	325				

a. 0 cells (0,0%) have expected count less than 5. The minimum expected count is 12,64.
b. Computed only for a 2x2 table

Symmetric Measures

		Value	Approximate Significance
Nominal by Nominal	Phi	,232	<,001
	Cramer's V	,232	<,001
	Contingency Coefficient	,226	<,001
N of Valid Cases		325	

toilet_before * symptoms_athletics

Crosstab

			symptoms_athletics		
			no	yes	Total
toilet_before	no	Count	64a	25a	89
		Expected Count	58,6	30,4	89,0
		% within toilet_before	71,9%	28,1%	100,0%
		% within symptoms_athletics	29,9%	22,5%	27,4%
		% of Total	19,7%	7,7%	27,4%
		Standardized Residual	,7	-1,0	
	yes	Count	150a	86a	236
		Expected Count	155,4	80,6	236,0
		% within toilet_before	63,6%	36,4%	100,0%

	% within symptoms_athletics	70,1%	77,5%	72,6%
	% of Total	46,2%	26,5%	72,6%
	Standardized Residual	-,4	,6	
Total	Count	214	111	325
	Expected Count	214,0	111,0	325,0
	% within toilet_before	65,8%	34,2%	100,0%
	% within symptoms_athletics	100,0%	100,0%	100,0%
	% of Total	65,8%	34,2%	100,0%

Each subscript letter denotes a subset of symptoms_athletics categories whose column proportions do not differ significantly from each other at the ,05 level.

Chi-Square Tests

	Value	df	Asymptotic Significance (2-sided)	Exact Sig. (2-sided)	Exact Sig. (1-sided)
Pearson Chi-Square	2,004[a]	1	,157		
Continuity Correction[b]	1,650	1	,199		
Likelihood Ratio	2,046	1	,153		
Fisher's Exact Test				,190	,099
Linear-by-Linear Association	1,998	1	,158		
N of Valid Cases	325				

a. 0 cells (0,0%) have expected count less than 5. The minimum expected count is 30,40.
b. Computed only for a 2x2 table

Symmetric Measures

		Value	Approximate Significance
Nominal by Nominal	Phi	,079	,157
	Cramer's V	,079	,157
	Contingency Coefficient	,078	,157
N of Valid Cases		325	

reduce_liquid * symptoms_athletics

Crosstab

			symptoms_athletics		
			no	yes	Total
reduce_liquid	no	Count	161_a	80_a	241
		Expected Count	158,7	82,3	241,0
		% within reduce_liquid	66,8%	33,2%	100,0%
		% within symptoms_athletics	75,2%	72,1%	74,2%
		% of Total	49,5%	24,6%	74,2%
		Standardized Residual	,2	-,3	
	yes	Count	53_a	31_a	84
		Expected Count	55,3	28,7	84,0
		% within reduce_liquid	63,1%	36,9%	100,0%
		% within symptoms_athletics	24,8%	27,9%	25,8%
		% of Total	16,3%	9,5%	25,8%
		Standardized Residual	-,3	,4	
Total		Count	214	111	325
		Expected Count	214,0	111,0	325,0
		% within reduce_liquid	65,8%	34,2%	100,0%
		% within symptoms_athletics	100,0%	100,0%	100,0%
		% of Total	65,8%	34,2%	100,0%

Each subscript letter denotes a subset of symptoms_athletics categories whose column proportions do not differ significantly from each other at the ,05 level.

Chi-Square Tests

	Value	df	Asymptotic Significance (2-sided)	Exact Sig. (2-sided)	Exact Sig. (1-sided)
Pearson Chi-Square	,381[a]	1	,537		
Continuity Correction[b]	,234	1	,629		
Likelihood Ratio	,378	1	,539		
Fisher's Exact Test				,593	,312
Linear-by-Linear Association	,380	1	,538		
N of Valid Cases	325				

a. 0 cells (0,0%) have expected count less than 5. The minimum expected count is 28,69.
b. Computed only for a 2x2 table

Symmetric Measures

		Value	Approximate Significance
Nominal by Nominal	Phi	,034	,537
	Cramer's V	,034	,537
	Contingency Coefficient	,034	,537
N of Valid Cases		325	

toilet_training * symptoms_athletics

Crosstab

			symptoms_athletics		Total
			no	yes	
toilet_training	no	Count	148_a	60_b	208
		Expected Count	137,0	71,0	208,0
		% within toilet_training	71,2%	28,8%	100,0%
		% within symptoms_athletics	69,2%	54,1%	64,0%
		% of Total	45,5%	18,5%	64,0%
		Standardized Residual	,9	-1,3	
	yes	Count	66_a	51_b	117
		Expected Count	77,0	40,0	117,0
		% within toilet_training	56,4%	43,6%	100,0%
		% within symptoms_athletics	30,8%	45,9%	36,0%
		% of Total	20,3%	15,7%	36,0%
		Standardized Residual	-1,3	1,7	
Total		Count	214	111	325
		Expected Count	214,0	111,0	325,0
		% within toilet_training	65,8%	34,2%	100,0%
		% within symptoms_athletics	100,0%	100,0%	100,0%
		% of Total	65,8%	34,2%	100,0%

Each subscript letter denotes a subset of symptoms_athletics categories whose column proportions do not differ significantly from each other at the ,05 level.

Chi-Square Tests

	Value	df	Asymptotic Significance (2-sided)	Exact Sig. (2-sided)	Exact Sig. (1-sided)
Pearson Chi-Square	7,238[a]	1	,007		
Continuity Correction[b]	6,597	1	,010		
Likelihood Ratio	7,145	1	,008		
Fisher's Exact Test				,010	,005
Linear-by-Linear Association	7,215	1	,007		
N of Valid Cases	325				

a. 0 cells (0,0%) have expected count less than 5. The minimum expected count is 39,96.
b. Computed only for a 2x2 table

Symmetric Measures

		Value	Approximate Significance
Nominal by Nominal	Phi	,149	,007
	Cramer's V	,149	,007
	Contingency Coefficient	,148	,007
N of Valid Cases		325	

toilet_competition * symptoms_athletics

Crosstab

			symptoms_athletics		
			no	yes	Total
toilet_competition	no	Count	136_a	57_b	193
		Expected Count	127,1	65,9	193,0
		% within toilet_competition	70,5%	29,5%	100,0%
		% within symptoms_athletics	63,6%	51,4%	59,4%
		% of Total	41,8%	17,5%	59,4%
		Standardized Residual	,8	-1,1	
	yes	Count	78_a	54_b	132
		Expected Count	86,9	45,1	132,0
		% within toilet_competition	59,1%	40,9%	100,0%
		% within symptoms_athletics	36,4%	48,6%	40,6%
		% of Total	24,0%	16,6%	40,6%
		Standardized Residual	-1,0	1,3	
Total		Count	214	111	325
		Expected Count	214,0	111,0	325,0
		% within toilet_competition	65,8%	34,2%	100,0%
		% within symptoms_athletics	100,0%	100,0%	100,0%
		% of Total	65,8%	34,2%	100,0%

Each subscript letter denotes a subset of symptoms_athletics categories whose column proportions do not differ significantly from each other at the ,05 level.

Chi-Square Tests

	Value	df	Asymptotic Significance (2-sided)	Exact Sig. (2-sided)	Exact Sig. (1-sided)
Pearson Chi-Square	4,510[a]	1	,034		
Continuity Correction[b]	4,019	1	,045		
Likelihood Ratio	4,482	1	,034		
Fisher's Exact Test				,043	,023
Linear-by-Linear Association	4,496	1	,034		
N of Valid Cases	325				

a. 0 cells (0,0%) have expected count less than 5. The minimum expected count is 45,08.
b. Computed only for a 2x2 table

Symmetric Measures

		Value	Approximate Significance
Nominal by Nominal	Phi	,118	,034
	Cramer's V	,118	,034
	Contingency Coefficient	,117	,034
N of Valid Cases		325	

caffeine * symptoms_athletics

Crosstab

			symptoms_athletics		Total
			no	yes	
caffeine	no	Count	151_a	66_b	217
		Expected Count	142,9	74,1	217,0
		% within caffeine	69,6%	30,4%	100,0%
		% within symptoms_athletics	70,6%	59,5%	66,8%
		% of Total	46,5%	20,3%	66,8%
		Standardized Residual	,7	-,9	
	yes	Count	63_a	45_b	108
		Expected Count	71,1	36,9	108,0
		% within caffeine	58,3%	41,7%	100,0%
		% within symptoms_athletics	29,4%	40,5%	33,2%
		% of Total	19,4%	13,8%	33,2%
		Standardized Residual	-1,0	1,3	
Total		Count	214	111	325
		Expected Count	214,0	111,0	325,0
		% within caffeine	65,8%	34,2%	100,0%
		% within symptoms_athletics	100,0%	100,0%	100,0%
		% of Total	65,8%	34,2%	100,0%

Each subscript letter denotes a subset of symptoms_athletics categories whose column proportions do not differ significantly from each other at the ,05 level.

Chi-Square Tests

	Value	df	Asymptotic Significance (2-sided)	Exact Sig. (2-sided)	Exact Sig. (1-sided)
Pearson Chi-Square	4,060[a]	1	,044		
Continuity Correction[b]	3,575	1	,059		
Likelihood Ratio	4,005	1	,045		
Fisher's Exact Test				,048	,030
Linear-by-Linear Association	4,047	1	,044		
N of Valid Cases	325				

a. 0 cells (0,0%) have expected count less than 5. The minimum expected count is 36,89.
b. Computed only for a 2x2 table

Symmetric Measures

		Value	Approximate Significance
Nominal by Nominal	Phi	,112	,044
	Cramer's V	,112	,044
	Contingency Coefficient	,111	,044
N of Valid Cases		325	

event=combined events * symptoms_athletics

Crosstab

			symptoms_athletics		Total
			no	yes	
event=combined events	others	Count	209_a	109_a	318
		Expected Count	209,4	108,6	318,0
		% within event=combined events	65,7%	34,3%	100,0%
		% within symptoms_athletics	97,7%	98,2%	97,8%
		% of Total	64,3%	33,5%	97,8%
		Standardized Residual	,0	,0	
	combined	Count	5_a	2_a	7
		Expected Count	4,6	2,4	7,0
		% within event=combined events	71,4%	28,6%	100,0%

		% within symptoms_athletics	2,3%	1,8%	2,2%
		% of Total	1,5%	0,6%	2,2%
		Standardized Residual	,2	-,3	
Total		Count	214	111	325
		Expected Count	214,0	111,0	325,0
		% within event=combined events	65,8%	34,2%	100,0%
		% within symptoms_athletics	100,0%	100,0%	100,0%
		% of Total	65,8%	34,2%	100,0%

Each subscript letter denotes a subset of symptoms_athletics categories whose column proportions do not differ significantly from each other at the ,05 level.

Chi-Square Tests

	Value	df	Asymptotic Significance (2-sided)	Exact Sig. (2-sided)	Exact Sig. (1-sided)
Pearson Chi-Square	,099[a]	1	,753		
Continuity Correction[b]	,000	1	1,000		
Likelihood Ratio	,102	1	,749		
Fisher's Exact Test				1,000	,552
Linear-by-Linear Association	,099	1	,753		
N of Valid Cases	325				

a. 2 cells (50,0%) have expected count less than 5. The minimum expected count is 2,39.
b. Computed only for a 2x2 table

Symmetric Measures

		Value	Approximate Significance
Nominal by Nominal	Phi	-,017	,753
	Cramer's V	,017	,753
	Contingency Coefficient	,017	,753
N of Valid Cases		325	

event=hurdles * symptoms_athletics

Crosstab

			symptoms_athletics		
			no	yes	Total
event=hurdles	others	Count	191$_a$	95$_a$	286
		Expected Count	188,3	97,7	286,0
		% within event=hurdles	66,8%	33,2%	100,0%
		% within symptoms_athletics	89,3%	85,6%	88,0%
		% of Total	58,8%	29,2%	88,0%
		Standardized Residual	,2	-,3	
	hurdles	Count	23$_a$	16$_a$	39
		Expected Count	25,7	13,3	39,0
		% within event=hurdles	59,0%	41,0%	100,0%
		% within symptoms_athletics	10,7%	14,4%	12,0%
		% of Total	7,1%	4,9%	12,0%
		Standardized Residual	-,5	,7	
Total		Count	214	111	325
		Expected Count	214,0	111,0	325,0
		% within event=hurdles	65,8%	34,2%	100,0%
		% within symptoms_athletics	100,0%	100,0%	100,0%
		% of Total	65,8%	34,2%	100,0%

Each subscript letter denotes a subset of symptoms_athletics categories whose column proportions do not differ significantly from each other at the ,05 level.

Chi-Square Tests

	Value	df	Asymptotic Significance (2-sided)	Exact Sig. (2-sided)	Exact Sig. (1-sided)
Pearson Chi-Square	,931[a]	1	,335		
Continuity Correction[b]	,616	1	,433		
Likelihood Ratio	,908	1	,341		
Fisher's Exact Test				,370	,215
Linear-by-Linear Association	,928	1	,335		
N of Valid Cases	325				

a. 0 cells (0,0%) have expected count less than 5. The minimum expected count is 13,32.
b. Computed only for a 2x2 table

Symmetric Measures

		Value	Approximate Significance
Nominal by Nominal	Phi	,054	,335
	Cramer's V	,054	,335
	Contingency Coefficient	,053	,335
N of Valid Cases		325	

event=jumps * symptoms_athletics

Crosstab

			symptoms_athletics		
			no	yes	Total
event=jumps	others	Count	189_a	98_a	287
		Expected Count	189,0	98,0	287,0
		% within event=jumps	65,9%	34,1%	100,0%
		% within symptoms_athletics	88,3%	88,3%	88,3%
		% of Total	58,2%	30,2%	88,3%
		Standardized Residual	,0	,0	
	jumps	Count	25_a	13_a	38
		Expected Count	25,0	13,0	38,0
		% within event=jumps	65,8%	34,2%	100,0%
		% within symptoms_athletics	11,7%	11,7%	11,7%
		% of Total	7,7%	4,0%	11,7%
		Standardized Residual	,0	,0	
Total		Count	214	111	325
		Expected Count	214,0	111,0	325,0
		% within event=jumps	65,8%	34,2%	100,0%
		% within symptoms_athletics	100,0%	100,0%	100,0%
		% of Total	65,8%	34,2%	100,0%

Each subscript letter denotes a subset of symptoms_athletics categories whose column proportions do not differ significantly from each other at the ,05 level.

Chi-Square Tests

	Value	df	Asymptotic Significance (2-sided)	Exact Sig. (2-sided)	Exact Sig. (1-sided)
Pearson Chi-Square	,000[a]	1	,994		
Continuity Correction[b]	,000	1	1,000		
Likelihood Ratio	,000	1	,994		
Fisher's Exact Test				1,000	,563
Linear-by-Linear Association	,000	1	,994		
N of Valid Cases	325				

a. 0 cells (0,0%) have expected count less than 5. The minimum expected count is 12,98.
b. Computed only for a 2x2 table

Symmetric Measures

		Value	Approximate Significance
Nominal by Nominal	Phi	,000	,994
	Cramer's V	,000	,994
	Contingency Coefficient	,000	,994
N of Valid Cases		325	

event=long distance runs * symptoms_athletics

Crosstab

			symptoms_athletics	
			no	yes
event=long distance runs	others	Count	205_a	101_a
		Expected Count	201,5	104,5
		% within event=long distance runs	67,0%	33,0%
		% within symptoms_athletics	95,8%	91,0%
		% of Total	63,1%	31,1%
		Standardized Residual	,2	-,3
	long distance	Count	9_a	10_a
		Expected Count	12,5	6,5
		% within event=long distance runs	47,4%	52,6%
		% within symptoms_athletics	4,2%	9,0%
		% of Total	2,8%	3,1%
		Standardized Residual	-1,0	1,4
Total		Count	214	111
		Expected Count	214,0	111,0
		% within event=long distance runs	65,8%	34,2%
		% within symptoms_athletics	100,0%	100,0%
		% of Total	65,8%	34,2%

Crosstab

			Total
event=long distance runs	others	Count	306
		Expected Count	306,0
		% within event=long distance runs	100,0%
		% within symptoms_athletics	94,2%
		% of Total	94,2%
		Standardized Residual	
	long distance	Count	19
		Expected Count	19,0
		% within event=long distance runs	100,0%
		% within symptoms_athletics	5,8%
		% of Total	5,8%
		Standardized Residual	
Total		Count	325
		Expected Count	325,0
		% within event=long distance runs	100,0%
		% within symptoms_athletics	100,0%
		% of Total	100,0%

Each subscript letter denotes a subset of symptoms_athletics categories whose column proportions do not differ significantly from each other at the ,05 level.

Chi-Square Tests

	Value	df	Asymptotic Significance (2-sided)	Exact Sig. (2-sided)	Exact Sig. (1-sided)
Pearson Chi-Square	3,064[a]	1	,080		
Continuity Correction[b]	2,253	1	,133		
Likelihood Ratio	2,900	1	,089		
Fisher's Exact Test				,087	,069
Linear-by-Linear Association	3,054	1	,081		
N of Valid Cases	325				

a. 0 cells (0,0%) have expected count less than 5. The minimum expected count is 6,49.
b. Computed only for a 2x2 table

Symmetric Measures

		Value	Approximate Significance
Nominal by Nominal	Phi	,097	,080
	Cramer's V	,097	,080
	Contingency Coefficient	,097	,080
N of Valid Cases		325	

event=middle distance runs * symptoms_athletics

Crosstab

			symptoms_athletics no	symptoms_athletics yes
event=middle distance runs	others	Count	192_a	94_a
		Expected Count	188,3	97,7
		% within event=middle distance runs	67,1%	32,9%
		% within symptoms_athletics	89,7%	84,7%
		% of Total	59,1%	28,9%
		Standardized Residual	,3	-,4
	middle distance	Count	22_a	17_a
		Expected Count	25,7	13,3
		% within event=middle distance runs	56,4%	43,6%
		% within symptoms_athletics	10,3%	15,3%
		% of Total	6,8%	5,2%
		Standardized Residual	-,7	1,0
Total		Count	214	111
		Expected Count	214,0	111,0
		% within event=middle distance runs	65,8%	34,2%
		% within symptoms_athletics	100,0%	100,0%
		% of Total	65,8%	34,2%

Crosstab

			Total
event=middle distance runs	others	Count	286
		Expected Count	286,0
		% within event=middle distance runs	100,0%
		% within symptoms_athletics	88,0%
		% of Total	88,0%
		Standardized Residual	
	middle distance	Count	39
		Expected Count	39,0
		% within event=middle distance runs	100,0%
		% within symptoms_athletics	12,0%
		% of Total	12,0%
		Standardized Residual	

Total	Count	325
	Expected Count	325,0
	% within event=middle distance runs	100,0%
	% within symptoms_athletics	100,0%
	% of Total	100,0%

Each subscript letter denotes a subset of symptoms_athletics categories whose column proportions do not differ significantly from each other at the ,05 level.

Chi-Square Tests

	Value	df	Asymptotic Significance (2-sided)	Exact Sig. (2-sided)	Exact Sig. (1-sided)
Pearson Chi-Square	1,755[a]	1	,185		
Continuity Correction[b]	1,310	1	,252		
Likelihood Ratio	1,701	1	,192		
Fisher's Exact Test				,209	,127
Linear-by-Linear Association	1,749	1	,186		
N of Valid Cases	325				

a. 0 cells (0,0%) have expected count less than 5. The minimum expected count is 13,32.
b. Computed only for a 2x2 table

Symmetric Measures

		Value	Approximate Significance
Nominal by Nominal	Phi	,073	,185
	Cramer's V	,073	,185
	Contingency Coefficient	,073	,185
N of Valid Cases		325	

event=race walking * symptoms_athletics

Crosstab

			symptoms_athletics		Total
			no	yes	
event=race walking	others	Count	199_a	100_a	299
		Expected Count	196,9	102,1	299,0
		% within event=race walking	66,6%	33,4%	100,0%
		% within symptoms_athletics	93,0%	90,1%	92,0%
		% of Total	61,2%	30,8%	92,0%
		Standardized Residual	,2	-,2	
	race walking	Count	15_a	11_a	26
		Expected Count	17,1	8,9	26,0
		% within event=race walking	57,7%	42,3%	100,0%
		% within symptoms_athletics	7,0%	9,9%	8,0%
		% of Total	4,6%	3,4%	8,0%
		Standardized Residual	-,5	,7	
Total		Count	214	111	325
		Expected Count	214,0	111,0	325,0
		% within event=race walking	65,8%	34,2%	100,0%
		% within symptoms_athletics	100,0%	100,0%	100,0%
		% of Total	65,8%	34,2%	100,0%

Each subscript letter denotes a subset of symptoms_athletics categories whose column proportions do not differ significantly from each other at the ,05 level.

Chi-Square Tests

	Value	df	Asymptotic Significance (2-sided)	Exact Sig. (2-sided)	Exact Sig. (1-sided)
Pearson Chi-Square	,835[a]	1	,361		
Continuity Correction[b]	,488	1	,485		
Likelihood Ratio	,811	1	,368		

				Exact Sig. (2-sided)	Exact Sig. (1-sided)
Fisher's Exact Test				,392	,240
Linear-by-Linear Association	,833	1	,361		
N of Valid Cases	325				

a. 0 cells (0,0%) have expected count less than 5. The minimum expected count is 8,88.
b. Computed only for a 2x2 table

Symmetric Measures

		Value	Approximate Significance
Nominal by Nominal	Phi	,051	,361
	Cramer's V	,051	,361
	Contingency Coefficient	,051	,361
N of Valid Cases		325	

event=throws * symptoms_athletics

Crosstab

			symptoms_athletics		Total
			no	yes	
event=throws	others	Count	190_a	106_b	296
		Expected Count	194,9	101,1	296,0
		% within event=throws	64,2%	35,8%	100,0%
		% within symptoms_athletics	88,8%	95,5%	91,1%
		% of Total	58,5%	32,6%	91,1%
		Standardized Residual	-,4	,5	
	throws	Count	24_a	5_b	29
		Expected Count	19,1	9,9	29,0
		% within event=throws	82,8%	17,2%	100,0%
		% within symptoms_athletics	11,2%	4,5%	8,9%
		% of Total	7,4%	1,5%	8,9%
		Standardized Residual	1,1	-1,6	
Total		Count	214	111	325
		Expected Count	214,0	111,0	325,0
		% within event=throws	65,8%	34,2%	100,0%
		% within symptoms_athletics	100,0%	100,0%	100,0%
		% of Total	65,8%	34,2%	100,0%

Each subscript letter denotes a subset of symptoms_athletics categories whose column proportions do not differ significantly from each other at the ,05 level.

Chi-Square Tests

	Value	df	Asymptotic Significance (2-sided)	Exact Sig. (2-sided)	Exact Sig. (1-sided)
Pearson Chi-Square	4,050[a]	1	,044		
Continuity Correction[b]	3,266	1	,071		
Likelihood Ratio	4,496	1	,034		
Fisher's Exact Test				,063	,031
Linear-by-Linear Association	4,037	1	,045		
N of Valid Cases	325				

a. 0 cells (0,0%) have expected count less than 5. The minimum expected count is 9,90.
b. Computed only for a 2x2 table

Symmetric Measures

		Value	Approximate Significance
Nominal by Nominal	Phi	-,112	,044
	Cramer's V	,112	,044
	Contingency Coefficient	,111	,044
N of Valid Cases		325	

health_conditions=constipation * symptoms_athletics

Crosstab

			symptoms_athletics	
			no	yes
health_conditions=constipation	others	Count	214_a	105_b
		Expected Count	210,0	109,0
		% within health_conditions=constipation	67,1%	32,9%
		% within symptoms_athletics	100,0%	94,6%
		% of Total	65,8%	32,3%
		Standardized Residual	,3	-,4
	constipations	Count	0_a	6_b
		Expected Count	4,0	2,0
		% within health_conditions=constipation	0,0%	100,0%
		% within symptoms_athletics	0,0%	5,4%
		% of Total	0,0%	1,8%
		Standardized Residual	-2,0	2,8
Total		Count	214	111
		Expected Count	214,0	111,0
		% within health_conditions=constipation	65,8%	34,2%
		% within symptoms_athletics	100,0%	100,0%
		% of Total	65,8%	34,2%

Crosstab

			Total
health_conditions=constipation	others	Count	319
		Expected Count	319,0
		% within health_conditions=constipation	100,0%
		% within symptoms_athletics	98,2%
		% of Total	98,2%
		Standardized Residual	
	constipations	Count	6
		Expected Count	6,0
		% within health_conditions=constipation	100,0%
		% within symptoms_athletics	1,8%
		% of Total	1,8%
		Standardized Residual	
Total		Count	325
		Expected Count	325,0
		% within health_conditions=constipation	100,0%
		% within symptoms_athletics	100,0%
		% of Total	100,0%

Each subscript letter denotes a subset of symptoms_athletics categories whose column proportions do not differ significantly from each other at the ,05 level.

Chi-Square Tests

	Value	df	Asymptotic Significance (2-sided)	Exact Sig. (2-sided)	Exact Sig. (1-sided)
Pearson Chi-Square	11,785[a]	1	<,001		
Continuity Correction[b]	8,991	1	,003		

Likelihood Ratio	13,110	1	<,001		
Fisher's Exact Test				,001	,001
Linear-by-Linear Association	11,749	1	<,001		
N of Valid Cases	325				

a. 2 cells (50,0%) have expected count less than 5. The minimum expected count is 2,05.
b. Computed only for a 2x2 table

Symmetric Measures

		Value	Approximate Significance
Nominal by Nominal	Phi	,190	<,001
	Cramer's V	,190	<,001
	Contingency Coefficient	,187	<,001
N of Valid Cases		325	

health_conditions=diabetes * symptoms_athletics

Crosstab

			symptoms_athletics		Total
			no	yes	
health_conditions=diabetes	others	Count	213_a	111_a	324
		Expected Count	213,3	110,7	324,0
		% within health_conditions=diabetes	65,7%	34,3%	100,0%
		% within symptoms_athletics	99,5%	100,0%	99,7%
		% of Total	65,5%	34,2%	99,7%
		Standardized Residual	,0	,0	
	diabetes	Count	1_a	0_a	1
		Expected Count	,7	,3	1,0
		% within health_conditions=diabetes	100,0%	0,0%	100,0%
		% within symptoms_athletics	0,5%	0,0%	0,3%
		% of Total	0,3%	0,0%	0,3%
		Standardized Residual	,4	-,6	
Total		Count	214	111	325
		Expected Count	214,0	111,0	325,0
		% within health_conditions=diabetes	65,8%	34,2%	100,0%
		% within symptoms_athletics	100,0%	100,0%	100,0%
		% of Total	65,8%	34,2%	100,0%

Each subscript letter denotes a subset of symptoms_athletics categories whose column proportions do not differ significantly from each other at the ,05 level.

Chi-Square Tests

	Value	df	Asymptotic Significance (2-sided)	Exact Sig. (2-sided)	Exact Sig. (1-sided)
Pearson Chi-Square	,520[a]	1	,471		
Continuity Correction[b]	,000	1	1,000		
Likelihood Ratio	,837	1	,360		
Fisher's Exact Test				1,000	,658
Linear-by-Linear Association	,519	1	,471		
N of Valid Cases	325				

a. 2 cells (50,0%) have expected count less than 5. The minimum expected count is ,34.
b. Computed only for a 2x2 table

Symmetric Measures

		Value	Approximate Significance
Nominal by Nominal	Phi	-,040	,471
	Cramer's V	,040	,471

Contingency Coefficient	,040		,471
N of Valid Cases	325		

health_conditions=frequent UI * symptoms_athletics

Crosstab

			symptoms_athletics	
			no	yes
health_conditions=frequent UI	others	Count	209_a	105_a
		Expected Count	206,8	107,2
		% within health_conditions=frequent UI	66,6%	33,4%
		% within symptoms_athletics	97,7%	94,6%
		% of Total	64,3%	32,3%
		Standardized Residual	,2	-,2
	Frequent UI	Count	5_a	6_a
		Expected Count	7,2	3,8
		% within health_conditions=frequent UI	45,5%	54,5%
		% within symptoms_athletics	2,3%	5,4%
		% of Total	1,5%	1,8%
		Standardized Residual	-,8	1,2
Total		Count	214	111
		Expected Count	214,0	111,0
		% within health_conditions=frequent UI	65,8%	34,2%
		% within symptoms_athletics	100,0%	100,0%
		% of Total	65,8%	34,2%

Crosstab

			Total
health_conditions=frequent UI	others	Count	314
		Expected Count	314,0
		% within health_conditions=frequent UI	100,0%
		% within symptoms_athletics	96,6%
		% of Total	96,6%
		Standardized Residual	
	Frequent UI	Count	11
		Expected Count	11,0
		% within health_conditions=frequent UI	100,0%
		% within symptoms_athletics	3,4%
		% of Total	3,4%
		Standardized Residual	
Total		Count	325
		Expected Count	325,0
		% within health_conditions=frequent UI	100,0%
		% within symptoms_athletics	100,0%
		% of Total	100,0%

Each subscript letter denotes a subset of symptoms_athletics categories whose column proportions do not differ significantly from each other at the ,05 level.

Chi-Square Tests

	Value	df	Asymptotic Significance (2-sided)	Exact Sig. (2-sided)	Exact Sig. (1-sided)
Pearson Chi-Square	2,105[a]	1	,147		
Continuity Correction[b]	1,271	1	,260		
Likelihood Ratio	1,983	1	,159		
Fisher's Exact Test				,195	,131
Linear-by-Linear Association	2,099	1	,147		
N of Valid Cases	325				

a. 1 cells (25,0%) have expected count less than 5. The minimum expected count is 3,76.

b. Computed only for a 2x2 table

Symmetric Measures

		Value	Approximate Significance
Nominal by Nominal	Phi	,080	,147
	Cramer's V	,080	,147
	Contingency Coefficient	,080	,147
N of Valid Cases		325	

health_conditions=heart arrhytmia * symptoms_athletics

Crosstab

			symptoms_athletics no	symptoms_athletics yes
health_conditions=heart arrhytmia	others	Count	213_a	111_a
		Expected Count	213,3	110,7
		% within health_conditions=heart arrhytmia	65,7%	34,3%
		% within symptoms_athletics	99,5%	100,0%
		% of Total	65,5%	34,2%
		Standardized Residual	,0	,0
	heart arrhytmia	Count	1_a	0_a
		Expected Count	,7	,3
		% within health_conditions=heart arrhytmia	100,0%	0,0%
		% within symptoms_athletics	0,5%	0,0%
		% of Total	0,3%	0,0%
		Standardized Residual	,4	-,6
Total		Count	214	111
		Expected Count	214,0	111,0
		% within health_conditions=heart arrhytmia	65,8%	34,2%
		% within symptoms_athletics	100,0%	100,0%
		% of Total	65,8%	34,2%

Crosstab

			Total
health_conditions=heart arrhytmia	others	Count	324
		Expected Count	324,0
		% within health_conditions=heart arrhytmia	100,0%
		% within symptoms_athletics	99,7%
		% of Total	99,7%
		Standardized Residual	
	heart arrhytmia	Count	1
		Expected Count	1,0

		% within health_conditions=heart arrhytmia	100,0%
		% within symptoms_athletics	0,3%
		% of Total	0,3%
		Standardized Residual	
Total		Count	325
		Expected Count	325,0
		% within health_conditions=heart arrhytmia	100,0%
		% within symptoms_athletics	100,0%
		% of Total	100,0%

Each subscript letter denotes a subset of symptoms_athletics categories whose column proportions do not differ significantly from each other at the ,05 level.

Chi-Square Tests

	Value	df	Asymptotic Significance (2-sided)	Exact Sig. (2-sided)	Exact Sig. (1-sided)
Pearson Chi-Square	,520[a]	1	,471		
Continuity Correction[b]	,000	1	1,000		
Likelihood Ratio	,837	1	,360		
Fisher's Exact Test				1,000	,658
Linear-by-Linear Association	,519	1	,471		
N of Valid Cases	325				

a. 2 cells (50,0%) have expected count less than 5. The minimum expected count is ,34.
b. Computed only for a 2x2 table

Symmetric Measures

		Value	Approximate Significance
Nominal by Nominal	Phi	-,040	,471
	Cramer's V	,040	,471
	Contingency Coefficient	,040	,471
N of Valid Cases		325	

health_conditions=pelvic surgery * symptoms_athletics

Crosstab

			symptoms_athletics	
			no	yes
health_conditions=pelvic surgery	others	Count	214_a	109_b
		Expected Count	212,7	110,3
		% within health_conditions=pelvic surgery	66,3%	33,7%
		% within symptoms_athletics	100,0%	98,2%
		% of Total	65,8%	33,5%
		Standardized Residual	,1	-,1
	pelvic surgery	Count	0_a	2_b
		Expected Count	1,3	,7
		% within health_conditions=pelvic surgery	0,0%	100,0%
		% within symptoms_athletics	0,0%	1,8%
		% of Total	0,0%	0,6%
		Standardized Residual	-1,1	1,6
Total		Count	214	111
		Expected Count	214,0	111,0
		% within health_conditions=pelvic surgery	65,8%	34,2%
		% within symptoms_athletics	100,0%	100,0%

		% of Total	65,8%	34,2%

Crosstab

			Total
health_conditions=pelvic surgery	others	Count	323
		Expected Count	323,0
		% within health_conditions=pelvic surgery	100,0%
		% within symptoms_athletics	99,4%
		% of Total	99,4%
		Standardized Residual	
	pelvic surgery	Count	2
		Expected Count	2,0
		% within health_conditions=pelvic surgery	100,0%
		% within symptoms_athletics	0,6%
		% of Total	0,6%
		Standardized Residual	
Total		Count	325
		Expected Count	325,0
		% within health_conditions=pelvic surgery	100,0%
		% within symptoms_athletics	100,0%
		% of Total	100,0%

Each subscript letter denotes a subset of symptoms_athletics categories whose column proportions do not differ significantly from each other at the ,05 level.

Chi-Square Tests

	Value	df	Asymptotic Significance (2-sided)	Exact Sig. (2-sided)	Exact Sig. (1-sided)
Pearson Chi-Square	3,880[a]	1	,049		
Continuity Correction[b]	1,493	1	,222		
Likelihood Ratio	4,321	1	,038		
Fisher's Exact Test				,116	,116
Linear-by-Linear Association	3,868	1	,049		
N of Valid Cases	325				

a. 2 cells (50,0%) have expected count less than 5. The minimum expected count is ,68.
b. Computed only for a 2x2 table

Symmetric Measures

		Value	Approximate Significance
Nominal by Nominal	Phi	,109	,049
	Cramer's V	,109	,049
	Contingency Coefficient	,109	,049
N of Valid Cases		325	

health_conditions=hypertension * symptoms_athletics

Crosstab

			symptoms_athletics	
			no	yes
health_conditions=hypertension	others	Count	214_a	109_b
		Expected Count	212,7	110,3

		% within health_conditions=hypertension	66,3%	33,7%
		% within symptoms_athletics	100,0%	98,2%
		% of Total	65,8%	33,5%
		Standardized Residual	,1	-,1
	hypertension	Count	0_a	2_b
		Expected Count	1,3	,7
		% within health_conditions=hypertension	0,0%	100,0%
		% within symptoms_athletics	0,0%	1,8%
		% of Total	0,0%	0,6%
		Standardized Residual	-1,1	1,6
Total		Count	214	111
		Expected Count	214,0	111,0
		% within health_conditions=hypertension	65,8%	34,2%
		% within symptoms_athletics	100,0%	100,0%
		% of Total	65,8%	34,2%

Crosstab

			Total
health_conditions=hypertension	others	Count	323
		Expected Count	323,0
		% within health_conditions=hypertension	100,0%
		% within symptoms_athletics	99,4%
		% of Total	99,4%
		Standardized Residual	
	hypertension	Count	2
		Expected Count	2,0
		% within health_conditions=hypertension	100,0%
		% within symptoms_athletics	0,6%
		% of Total	0,6%
		Standardized Residual	
Total		Count	325
		Expected Count	325,0
		% within health_conditions=hypertension	100,0%
		% within symptoms_athletics	100,0%
		% of Total	100,0%

Each subscript letter denotes a subset of symptoms_athletics categories whose column proportions do not differ significantly from each other at the ,05 level.

Chi-Square Tests

	Value	df	Asymptotic Significance (2-sided)	Exact Sig. (2-sided)	Exact Sig. (1-sided)
Pearson Chi-Square	3,880[a]	1	,049		
Continuity Correction[b]	1,493	1	,222		
Likelihood Ratio	4,321	1	,038		
Fisher's Exact Test				,116	,116
Linear-by-Linear Association	3,868	1	,049		
N of Valid Cases	325				

a. 2 cells (50,0%) have expected count less than 5. The minimum expected count is ,68.
b. Computed only for a 2x2 table

Symmetric Measures

		Value	Approximate Significance
Nominal by Nominal	Phi	,109	,049
	Cramer's V	,109	,049
	Contingency Coefficient	,109	,049
N of Valid Cases		325	

health_conditions=hyperthyrodism * symptoms_athletics

Crosstab

			symptoms_athletics	
			no	yes
health_conditions=hyperthyrodism	others	Count	214_a	110_a
		Expected Count	213,3	110,7
		% within health_conditions=hyperthyrodism	66,0%	34,0%
		% within symptoms_athletics	100,0%	99,1%
		% of Total	65,8%	33,8%
		Standardized Residual	,0	-,1
	hyperthyrodism	Count	0_a	1_a
		Expected Count	,7	,3
		% within health_conditions=hyperthyrodism	0,0%	100,0%
		% within symptoms_athletics	0,0%	0,9%
		% of Total	0,0%	0,3%
		Standardized Residual	-,8	1,1
Total		Count	214	111
		Expected Count	214,0	111,0
		% within health_conditions=hyperthyrodism	65,8%	34,2%
		% within symptoms_athletics	100,0%	100,0%
		% of Total	65,8%	34,2%

Crosstab

			Total
health_conditions=hyperthyrodism	others	Count	324
		Expected Count	324,0
		% within health_conditions=hyperthyrodism	100,0%
		% within symptoms_athletics	99,7%
		% of Total	99,7%
		Standardized Residual	
	hyperthyrodism	Count	1
		Expected Count	1,0
		% within health_conditions=hyperthyrodism	100,0%
		% within symptoms_athletics	0,3%
		% of Total	0,3%
		Standardized Residual	
Total		Count	325
		Expected Count	325,0
		% within health_conditions=hyperthyrodism	100,0%
		% within symptoms_athletics	100,0%
		% of Total	100,0%

Each subscript letter denotes a subset of symptoms_athletics categories whose column proportions do not differ significantly from each other at the ,05 level.

Chi-Square Tests

	Value	df	Asymptotic Significance (2-sided)	Exact Sig. (2-sided)	Exact Sig. (1-sided)
Pearson Chi-Square	1,934[a]	1	,164		
Continuity Correction[b]	,112	1	,738		
Likelihood Ratio	2,155	1	,142		
Fisher's Exact Test				,342	,342
Linear-by-Linear Association	1,928	1	,165		
N of Valid Cases	325				

a. 2 cells (50,0%) have expected count less than 5. The minimum expected count is ,34.
b. Computed only for a 2x2 table

Symmetric Measures

		Value	Approximate Significance
Nominal by Nominal	Phi	,077	,164
	Cramer's V	,077	,164
	Contingency Coefficient	,077	,164
N of Valid Cases		325	

health_conditions=respiratory issues * symptoms_athletics

Crosstab

			symptoms_athletics no	symptoms_athletics yes
health_conditions=respiratory issues	others	Count	200_a	100_a
		Expected Count	197,5	102,5
		% within health_conditions=respiratory issues	66,7%	33,3%
		% within symptoms_athletics	93,5%	90,1%
		% of Total	61,5%	30,8%
		Standardized Residual	,2	-,2
	respiratory issues	Count	14_a	11_a
		Expected Count	16,5	8,5
		% within health_conditions=respiratory issues	56,0%	44,0%
		% within symptoms_athletics	6,5%	9,9%
		% of Total	4,3%	3,4%
		Standardized Residual	-,6	,8
Total		Count	214	111
		Expected Count	214,0	111,0
		% within health_conditions=respiratory issues	65,8%	34,2%
		% within symptoms_athletics	100,0%	100,0%
		% of Total	65,8%	34,2%

Crosstab

			Total
health_conditions=respiratory issues	others	Count	300
		Expected Count	300,0
		% within health_conditions=respiratory issues	100,0%
		% within symptoms_athletics	92,3%
		% of Total	92,3%

		Standardized Residual	
	respiratory issues	Count	25
		Expected Count	25,0
		% within health_conditions=respiratory issues	100,0%
		% within symptoms_athletics	7,7%
		% of Total	7,7%
		Standardized Residual	
Total		Count	325
		Expected Count	325,0
		% within health_conditions=respiratory issues	100,0%
		% within symptoms_athletics	100,0%
		% of Total	100,0%

Each subscript letter denotes a subset of symptoms_athletics categories whose column proportions do not differ significantly from each other at the ,05 level.

Chi-Square Tests

	Value	df	Asymptotic Significance (2-sided)	Exact Sig. (2-sided)	Exact Sig. (1-sided)
Pearson Chi-Square	1,168[a]	1	,280		
Continuity Correction[b]	,741	1	,389		
Likelihood Ratio	1,128	1	,288		
Fisher's Exact Test				,281	,193
Linear-by-Linear Association	1,164	1	,281		
N of Valid Cases	325				

a. 0 cells (0,0%) have expected count less than 5. The minimum expected count is 8,54.
b. Computed only for a 2x2 table

Symmetric Measures

		Value	Approximate Significance
Nominal by Nominal	Phi	,060	,280
	Cramer's V	,060	,280
	Contingency Coefficient	,060	,280
N of Valid Cases		325	

stress_fractures=1 to 3 * symptoms_athletics

Crosstab

			symptoms_athletics		
			no	yes	Total
stress_fractures=1 to 3	others	Count	181_{a}	84_{b}	265
		Expected Count	174,5	90,5	265,0
		% within stress_fractures=1 to 3	68,3%	31,7%	100,0%
		% within symptoms_athletics	84,6%	75,7%	81,5%
		% of Total	55,7%	25,8%	81,5%
		Standardized Residual	,5	-,7	
	1 to 3	Count	33_{a}	27_{b}	60
		Expected Count	39,5	20,5	60,0
		% within stress_fractures=1 to 3	55,0%	45,0%	100,0%
		% within symptoms_athletics	15,4%	24,3%	18,5%
		% of Total	10,2%	8,3%	18,5%
		Standardized Residual	-1,0	1,4	
Total		Count	214	111	325
		Expected Count	214,0	111,0	325,0
		% within stress_fractures=1 to 3	65,8%	34,2%	100,0%
		% within symptoms_athletics	100,0%	100,0%	100,0%

% of Total	65,8%	34,2%	100,0%

Each subscript letter denotes a subset of symptoms_athletics categories whose column proportions do not differ significantly from each other at the ,05 level.

Chi-Square Tests

	Value	df	Asymptotic Significance (2-sided)	Exact Sig. (2-sided)	Exact Sig. (1-sided)
Pearson Chi-Square	3,849[a]	1	,050		
Continuity Correction[b]	3,280	1	,070		
Likelihood Ratio	3,733	1	,053		
Fisher's Exact Test				,070	,036
Linear-by-Linear Association	3,837	1	,050		
N of Valid Cases	325				

a. 0 cells (0,0%) have expected count less than 5. The minimum expected count is 20,49.
b. Computed only for a 2x2 table

Symmetric Measures

		Value	Approximate Significance
Nominal by Nominal	Phi	,109	,050
	Cramer's V	,109	,050
	Contingency Coefficient	,108	,050
N of Valid Cases		325	

stress_fractures=more than 3 * symptoms_athletics

Crosstab

			symptoms_athletics	
			no	yes
stress_fractures=more than 3	others	Count	210_a	106_a
		Expected Count	208,1	107,9
		% within stress_fractures=more than 3	66,5%	33,5%
		% within symptoms_athletics	98,1%	95,5%
		% of Total	64,6%	32,6%
		Standardized Residual	,1	-,2
	more than 3	Count	4_a	5_a
		Expected Count	5,9	3,1
		% within stress_fractures=more than 3	44,4%	55,6%
		% within symptoms_athletics	1,9%	4,5%
		% of Total	1,2%	1,5%
		Standardized Residual	-,8	1,1
Total		Count	214	111
		Expected Count	214,0	111,0
		% within stress_fractures=more than 3	65,8%	34,2%
		% within symptoms_athletics	100,0%	100,0%
		% of Total	65,8%	34,2%

Crosstab

			Total
stress_fractures=more than 3	others	Count	316
		Expected Count	316,0
		% within stress_fractures=more than 3	100,0%
		% within symptoms_athletics	97,2%

		% of Total	97,2%
		Standardized Residual	
	more than 3	Count	9
		Expected Count	9,0
		% within stress_fractures=more than 3	100,0%
		% within symptoms_athletics	2,8%
		% of Total	2,8%
		Standardized Residual	
Total		Count	325
		Expected Count	325,0
		% within stress_fractures=more than 3	100,0%
		% within symptoms_athletics	100,0%
		% of Total	100,0%

Each subscript letter denotes a subset of symptoms_athletics categories whose column proportions do not differ significantly from each other at the ,05 level.

Chi-Square Tests

	Value	df	Asymptotic Significance (2-sided)	Exact Sig. (2-sided)	Exact Sig. (1-sided)
Pearson Chi-Square	1,885[a]	1	,170		
Continuity Correction[b]	1,034	1	,309		
Likelihood Ratio	1,773	1	,183		
Fisher's Exact Test				,283	,155
Linear-by-Linear Association	1,879	1	,170		
N of Valid Cases	325				

a. 1 cells (25,0%) have expected count less than 5. The minimum expected count is 3,07.
b. Computed only for a 2x2 table

Symmetric Measures

		Value	Approximate Significance
Nominal by Nominal	Phi	,076	,170
	Cramer's V	,076	,170
	Contingency Coefficient	,076	,170
N of Valid Cases		325	

DIFFERENCES BETWEEN SYMPTOMATIC (ATHLETICS-RELATED URINARY INCONTINENCE) AND ASYMPTOMATIC ATHLETES.

A. Continuous variables

Variables	Level	SYMPTOMATIC (n=42)	ASYMPTOMATIC (n = 283)	*p*-value*
BMI (kg/m^2)	Median [Min – Max]	19.6 [16.8 – 28.6]	19.6 [16.8 – 28.60]	
	IQR	11.8	11.8	
Training (hours/day)	Median [Min – Max]	2 [1.5 – 4]	2.5 [1 – 8]	.069
	IQR	2.5	7	
Training (sessions/week)	Median [Min – Max]	6 [2 – 9]	6 [1 – 13]	.845
	IQR	7	12	

* Mann-Whitney test (non-parametric test) was used.

B. Categorial variables

sex * ui_athletics

Crosstab

			ui_athletics		
			no	yes	Total
sex	male	Count	126$_a$	7$_b$	133
		Expected Count	115,8	17,2	133,0
		% within sex	94,7%	5,3%	100,0%
		% within ui_athletics	44,5%	16,7%	40,9%
		% of Total	38,8%	2,2%	40,9%
		Standardized Residual	,9	-2,5	
	female	Count	157$_a$	35$_b$	192
		Expected Count	167,2	24,8	192,0
		% within sex	81,8%	18,2%	100,0%
		% within ui_athletics	55,5%	83,3%	59,1%
		% of Total	48,3%	10,8%	59,1%
		Standardized Residual	-,8	2,0	
Total		Count	283	42	325
		Expected Count	283,0	42,0	325,0
		% within sex	87,1%	12,9%	100,0%
		% within ui_athletics	100,0%	100,0%	100,0%
		% of Total	87,1%	12,9%	100,0%

Each subscript letter denotes a subset of ui_athletics categories whose column proportions do not differ significantly from each other at the ,05 level.

Chi-Square Tests

	Value	df	Asymptotic Significance (2-sided)	Exact Sig. (2-sided)	Exact Sig. (1-sided)
Pearson Chi-Square	11,739[a]	1	<,001		
Continuity Correction[b]	10,615	1	,001		
Likelihood Ratio	13,009	1	<,001		

Fisher's Exact Test				<,001	<,001
Linear-by-Linear Association	11,702	1	<,001		
N of Valid Cases	325				

a. 0 cells (0,0%) have expected count less than 5. The minimum expected count is 17,19.
b. Computed only for a 2x2 table

Symmetric Measures

		Value	Approximate Significance
Nominal by Nominal	Phi	,190	<,001
	Cramer's V	,190	<,001
	Contingency Coefficient	,187	<,001
N of Valid Cases		325	

medications * ui_athletics

Crosstab

			ui_athletics		
			no	yes	Total
medications	no	Count	275_a	42_a	317
		Expected Count	276,0	41,0	317,0
		% within medications	86,8%	13,2%	100,0%
		% within ui_athletics	97,2%	100,0%	97,5%
		% of Total	84,6%	12,9%	97,5%
		Standardized Residual	-,1	,2	
	yes	Count	8_a	0_a	8
		Expected Count	7,0	1,0	8,0
		% within medications	100,0%	0,0%	100,0%
		% within ui_athletics	2,8%	0,0%	2,5%
		% of Total	2,5%	0,0%	2,5%
		Standardized Residual	,4	-1,0	
Total		Count	283	42	325
		Expected Count	283,0	42,0	325,0
		% within medications	87,1%	12,9%	100,0%
		% within ui_athletics	100,0%	100,0%	100,0%
		% of Total	87,1%	12,9%	100,0%

Each subscript letter denotes a subset of ui_athletics categories whose column proportions do not differ significantly from each other at the ,05 level.

Chi-Square Tests

	Value	df	Asymptotic Significance (2-sided)	Exact Sig. (2-sided)	Exact Sig. (1-sided)
Pearson Chi-Square	1,217[a]	1	,270		
Continuity Correction[b]	,325	1	,569		
Likelihood Ratio	2,244	1	,134		
Fisher's Exact Test				,603	,326
Linear-by-Linear Association	1,213	1	,271		
N of Valid Cases	325				

a. 1 cells (25,0%) have expected count less than 5. The minimum expected count is 1,03.
b. Computed only for a 2x2 table

Symmetric Measures

		Value	Approximate Significance
Nominal by Nominal	Phi	-,061	,270
	Cramer's V	,061	,270
	Contingency Coefficient	,061	,270
N of Valid Cases		325	

smoking * ui_athletics

Crosstab

			ui_athletics		
			no	yes	Total
smoking	no	Count	276$_a$	42$_a$	318
		Expected Count	276,9	41,1	318,0
		% within smoking	86,8%	13,2%	100,0%
		% within ui_athletics	97,5%	100,0%	97,8%
		% of Total	84,9%	12,9%	97,8%
		Standardized Residual	-,1	,1	
	yes	Count	7$_a$	0$_a$	7
		Expected Count	6,1	,9	7,0
		% within smoking	100,0%	0,0%	100,0%
		% within ui_athletics	2,5%	0,0%	2,2%
		% of Total	2,2%	0,0%	2,2%
		Standardized Residual	,4	-1,0	
Total		Count	283	42	325
		Expected Count	283,0	42,0	325,0
		% within smoking	87,1%	12,9%	100,0%
		% within ui_athletics	100,0%	100,0%	100,0%
		% of Total	87,1%	12,9%	100,0%

Each subscript letter denotes a subset of ui_athletics categories whose column proportions do not differ significantly from each other at the ,05 level.

Chi-Square Tests

	Value	df	Asymptotic Significance (2-sided)	Exact Sig. (2-sided)	Exact Sig. (1-sided)
Pearson Chi-Square	1,062[a]	1	,303		
Continuity Correction[b]	,212	1	,645		
Likelihood Ratio	1,960	1	,162		
Fisher's Exact Test				,601	,376
Linear-by-Linear Association	1,058	1	,304		
N of Valid Cases	325				

a. 1 cells (25,0%) have expected count less than 5. The minimum expected count is ,90.
b. Computed only for a 2x2 table

Symmetric Measures

		Value	Approximate Significance
Nominal by Nominal	Phi	-,057	,303
	Cramer's V	,057	,303
	Contingency Coefficient	,057	,303
N of Valid Cases		325	

pelvic_injury * ui_athletics

Crosstab

			ui_athletics		
			no	yes	Total
pelvic_injury	no	Count	252$_a$	36$_a$	288
		Expected Count	250,8	37,2	288,0
		% within pelvic_injury	87,5%	12,5%	100,0%
		% within ui_athletics	89,0%	85,7%	88,6%
		% of Total	77,5%	11,1%	88,6%
		Standardized Residual	,1	-,2	
	yes	Count	31$_a$	6$_a$	37
		Expected Count	32,2	4,8	37,0
		% within pelvic_injury	83,8%	16,2%	100,0%
		% within ui_athletics	11,0%	14,3%	11,4%
		% of Total	9,5%	1,8%	11,4%

	Standardized Residual	-,2	,6	
Total	Count	283	42	325
	Expected Count	283,0	42,0	325,0
	% within pelvic_injury	87,1%	12,9%	100,0%
	% within ui_athletics	100,0%	100,0%	100,0%
	% of Total	87,1%	12,9%	100,0%

Each subscript letter denotes a subset of ui_athletics categories whose column proportions do not differ significantly from each other at the ,05 level.

Chi-Square Tests

	Value	df	Asymptotic Significance (2-sided)	Exact Sig. (2-sided)	Exact Sig. (1-sided)
Pearson Chi-Square	,402[a]	1	,526		
Continuity Correction[b]	,140	1	,708		
Likelihood Ratio	,380	1	,538		
Fisher's Exact Test				,601	,339
Linear-by-Linear Association	,401	1	,526		
N of Valid Cases	325				

a. 1 cells (25,0%) have expected count less than 5. The minimum expected count is 4,78.
b. Computed only for a 2x2 table

Symmetric Measures

		Value	Approximate Significance
Nominal by Nominal	Phi	,035	,526
	Cramer's V	,035	,526
	Contingency Coefficient	,035	,526
N of Valid Cases		325	

other_sports * ui_athletics

Crosstab

			ui_athletics		
			no	yes	Total
other_sports	no	Count	214_a	31_a	245
		Expected Count	213,3	31,7	245,0
		% within other_sports	87,3%	12,7%	100,0%
		% within ui_athletics	75,6%	73,8%	75,4%
		% of Total	65,8%	9,5%	75,4%
		Standardized Residual	,0	-,1	
	yes	Count	69_a	11_a	80
		Expected Count	69,7	10,3	80,0
		% within other_sports	86,3%	13,8%	100,0%
		% within ui_athletics	24,4%	26,2%	24,6%
		% of Total	21,2%	3,4%	24,6%
		Standardized Residual	-,1	,2	
Total		Count	283	42	325
		Expected Count	283,0	42,0	325,0
		% within other_sports	87,1%	12,9%	100,0%
		% within ui_athletics	100,0%	100,0%	100,0%
		% of Total	87,1%	12,9%	100,0%

Each subscript letter denotes a subset of ui_athletics categories whose column proportions do not differ significantly from each other at the ,05 level.

Chi-Square Tests

	Value	df	Asymptotic Significance (2-sided)	Exact Sig. (2-sided)	Exact Sig. (1-sided)
Pearson Chi-Square	,064[a]	1	,800		
Continuity Correction[b]	,004	1	,951		
Likelihood Ratio	,064	1	,801		
Fisher's Exact Test				,848	,466

Linear-by-Linear Association	,064	1	,800		
N of Valid Cases	325				

a. 0 cells (0,0%) have expected count less than 5. The minimum expected count is 10,34.
b. Computed only for a 2x2 table

Symmetric Measures

		Value	Approximate Significance
Nominal by Nominal	Phi	,014	,800
	Cramer's V	,014	,800
	Contingency Coefficient	,014	,800
N of Valid Cases		325	

pelvic_awareness * ui_athletics

Crosstab

			ui_athletics		Total
			no	yes	
pelvic_awareness	no	Count	208_a	22_b	230
		Expected Count	200,3	29,7	230,0
		% within pelvic_awareness	90,4%	9,6%	100,0%
		% within ui_athletics	73,5%	52,4%	70,8%
		% of Total	64,0%	6,8%	70,8%
		Standardized Residual	,5	-1,4	
	yes	Count	75_a	20_b	95
		Expected Count	82,7	12,3	95,0
		% within pelvic_awareness	78,9%	21,1%	100,0%
		% within ui_athletics	26,5%	47,6%	29,2%
		% of Total	23,1%	6,2%	29,2%
		Standardized Residual	-,8	2,2	
Total		Count	283	42	325
		Expected Count	283,0	42,0	325,0
		% within pelvic_awareness	87,1%	12,9%	100,0%
		% within ui_athletics	100,0%	100,0%	100,0%
		% of Total	87,1%	12,9%	100,0%

Each subscript letter denotes a subset of ui_athletics categories whose column proportions do not differ significantly from each other at the ,05 level.

Chi-Square Tests

	Value	df	Asymptotic Significance (2-sided)	Exact Sig. (2-sided)	Exact Sig. (1-sided)
Pearson Chi-Square	7,884[a]	1	,005		
Continuity Correction[b]	6,896	1	,009		
Likelihood Ratio	7,320	1	,007		
Fisher's Exact Test				,010	,005
Linear-by-Linear Association	7,860	1	,005		
N of Valid Cases	325				

a. 0 cells (0,0%) have expected count less than 5. The minimum expected count is 12,28.
b. Computed only for a 2x2 table

Symmetric Measures

		Value	Approximate Significance
Nominal by Nominal	Phi	,156	,005
	Cramer's V	,156	,005
	Contingency Coefficient	,154	,005
N of Valid Cases		325	

pfd_awareness * ui_athletics

Crosstab

			ui_athletics		
			no	yes	Total
pfd_awareness	no	Count	228$_a$	28$_b$	256
		Expected Count	222,9	33,1	256,0
		% within pfd_awareness	89,1%	10,9%	100,0%
		% within ui_athletics	80,6%	66,7%	78,8%
		% of Total	70,2%	8,6%	78,8%
		Standardized Residual	,3	-,9	
	yes	Count	55$_a$	14$_b$	69
		Expected Count	60,1	8,9	69,0
		% within pfd_awareness	79,7%	20,3%	100,0%
		% within ui_athletics	19,4%	33,3%	21,2%
		% of Total	16,9%	4,3%	21,2%
		Standardized Residual	-,7	1,7	
Total		Count	283	42	325
		Expected Count	283,0	42,0	325,0
		% within pfd_awareness	87,1%	12,9%	100,0%
		% within ui_athletics	100,0%	100,0%	100,0%
		% of Total	87,1%	12,9%	100,0%

Each subscript letter denotes a subset of ui_athletics categories whose column proportions do not differ significantly from each other at the ,05 level.

Chi-Square Tests

	Value	df	Asymptotic Significance (2-sided)	Exact Sig. (2-sided)	Exact Sig. (1-sided)
Pearson Chi-Square	4,225[a]	1	,040		
Continuity Correction[b]	3,434	1	,064		
Likelihood Ratio	3,847	1	,050		
Fisher's Exact Test				,045	,036
Linear-by-Linear Association	4,212	1	,040		
N of Valid Cases	325				

a. 0 cells (0,0%) have expected count less than 5. The minimum expected count is 8,92.
b. Computed only for a 2x2 table

Symmetric Measures

		Value	Approximate Significance
Nominal by Nominal	Phi	,114	,040
	Cramer's V	,114	,040
	Contingency Coefficient	,113	,040
N of Valid Cases		325	

difficult_urination * ui_athletics

Crosstab

			ui_athletics		
			no	yes	Total
difficult_urination	no	Count	264$_a$	34$_b$	298
		Expected Count	259,5	38,5	298,0
		% within difficult_urination	88,6%	11,4%	100,0%
		% within ui_athletics	93,3%	81,0%	91,7%
		% of Total	81,2%	10,5%	91,7%
		Standardized Residual	,3	-,7	
	yes	Count	19$_a$	8$_b$	27
		Expected Count	23,5	3,5	27,0
		% within difficult_urination	70,4%	29,6%	100,0%
		% within ui_athletics	6,7%	19,0%	8,3%
		% of Total	5,8%	2,5%	8,3%
		Standardized Residual	-,9	2,4	
Total		Count	283	42	325

	Expected Count	283,0	42,0	325,0
	% within difficult_urination	87,1%	12,9%	100,0%
	% within ui_athletics	100,0%	100,0%	100,0%
	% of Total	87,1%	12,9%	100,0%

Each subscript letter denotes a subset of ui_athletics categories whose column proportions do not differ significantly from each other at the ,05 level.

Chi-Square Tests

	Value	df	Asymptotic Significance (2-sided)	Exact Sig. (2-sided)	Exact Sig. (1-sided)
Pearson Chi-Square	7,304[a]	1	,007		
Continuity Correction[b]	5,774	1	,016		
Likelihood Ratio	5,810	1	,016		
Fisher's Exact Test				,013	,013
Linear-by-Linear Association	7,281	1	,007		
N of Valid Cases	325				

a. 1 cells (25,0%) have expected count less than 5. The minimum expected count is 3,49.
b. Computed only for a 2x2 table

Symmetric Measures

		Value	Approximate Significance
Nominal by Nominal	Phi	,150	,007
	Cramer's V	,150	,007
	Contingency Coefficient	,148	,007
N of Valid Cases		325	

push_bowel * ui_athletics

Crosstab

			ui_athletics		
			no	yes	Total
push_bowel	no	Count	254_a	34_a	288
		Expected Count	250,8	37,2	288,0
		% within push_bowel	88,2%	11,8%	100,0%
		% within ui_athletics	89,8%	81,0%	88,6%
		% of Total	78,2%	10,5%	88,6%
		Standardized Residual	,2	-,5	
	yes	Count	29_a	8_a	37
		Expected Count	32,2	4,8	37,0
		% within push_bowel	78,4%	21,6%	100,0%
		% within ui_athletics	10,2%	19,0%	11,4%
		% of Total	8,9%	2,5%	11,4%
		Standardized Residual	-,6	1,5	
Total		Count	283	42	325
		Expected Count	283,0	42,0	325,0
		% within push_bowel	87,1%	12,9%	100,0%
		% within ui_athletics	100,0%	100,0%	100,0%
		% of Total	87,1%	12,9%	100,0%

Each subscript letter denotes a subset of ui_athletics categories whose column proportions do not differ significantly from each other at the ,05 level.

Chi-Square Tests

	Value	df	Asymptotic Significance (2-sided)	Exact Sig. (2-sided)	Exact Sig. (1-sided)
Pearson Chi-Square	2,807[a]	1	,094		
Continuity Correction[b]	2,003	1	,157		
Likelihood Ratio	2,459	1	,117		
Fisher's Exact Test				,115	,084
Linear-by-Linear Association	2,799	1	,094		
N of Valid Cases	325				

a. 1 cells (25,0%) have expected count less than 5. The minimum expected count is 4,78.
b. Computed only for a 2x2 table

Symmetric Measures

		Value	Approximate Significance
Nominal by Nominal	Phi	,093	,094
	Cramer's V	,093	,094
	Contingency Coefficient	,093	,094
N of Valid Cases		325	

toilet_before * ui_athletics

Crosstab

			ui_athletics		
			no	yes	Total
toilet_before	no	Count	81$_a$	8$_a$	89
		Expected Count	77,5	11,5	89,0
		% within toilet_before	91,0%	9,0%	100,0%
		% within ui_athletics	28,6%	19,0%	27,4%
		% of Total	24,9%	2,5%	27,4%
		Standardized Residual	,4	-1,0	
	yes	Count	202$_a$	34$_a$	236
		Expected Count	205,5	30,5	236,0
		% within toilet_before	85,6%	14,4%	100,0%
		% within ui_athletics	71,4%	81,0%	72,6%
		% of Total	62,2%	10,5%	72,6%
		Standardized Residual	-,2	,6	
Total		Count	283	42	325
		Expected Count	283,0	42,0	325,0
		% within toilet_before	87,1%	12,9%	100,0%
		% within ui_athletics	100,0%	100,0%	100,0%
		% of Total	87,1%	12,9%	100,0%

Each subscript letter denotes a subset of ui_athletics categories whose column proportions do not differ significantly from each other at the ,05 level.

Chi-Square Tests

	Value	df	Asymptotic Significance (2-sided)	Exact Sig. (2-sided)	Exact Sig. (1-sided)
Pearson Chi-Square	1,686[a]	1	,194		
Continuity Correction[b]	1,239	1	,266		
Likelihood Ratio	1,798	1	,180		
Fisher's Exact Test				,265	,132
Linear-by-Linear Association	1,681	1	,195		
N of Valid Cases	325				

a. 0 cells (0,0%) have expected count less than 5. The minimum expected count is 11,50.
b. Computed only for a 2x2 table

Symmetric Measures

		Value	Approximate Significance
Nominal by Nominal	Phi	,072	,194
	Cramer's V	,072	,194
	Contingency Coefficient	,072	,194
N of Valid Cases		325	

reduce_liquid * ui_athletics

Crosstab

			ui_athletics		
			no	yes	Total
reduce_liquid	no	Count	218$_a$	23$_b$	241
		Expected Count	209,9	31,1	241,0
		% within reduce_liquid	90,5%	9,5%	100,0%

		% within ui_athletics	77,0%	54,8%	74,2%
		% of Total	67,1%	7,1%	74,2%
		Standardized Residual	,6	-1,5	
	yes	Count	65_a	19_b	84
		Expected Count	73,1	10,9	84,0
		% within reduce_liquid	77,4%	22,6%	100,0%
		% within ui_athletics	23,0%	45,2%	25,8%
		% of Total	20,0%	5,8%	25,8%
		Standardized Residual	-1,0	2,5	
Total		Count	283	42	325
		Expected Count	283,0	42,0	325,0
		% within reduce_liquid	87,1%	12,9%	100,0%
		% within ui_athletics	100,0%	100,0%	100,0%
		% of Total	87,1%	12,9%	100,0%

Each subscript letter denotes a subset of ui_athletics categories whose column proportions do not differ significantly from each other at the ,05 level.

Chi-Square Tests

	Value	df	Asymptotic Significance (2-sided)	Exact Sig. (2-sided)	Exact Sig. (1-sided)
Pearson Chi-Square	9,464[a]	1	,002		
Continuity Correction[b]	8,337	1	,004		
Likelihood Ratio	8,581	1	,003		
Fisher's Exact Test				,004	,003
Linear-by-Linear Association	9,435	1	,002		
N of Valid Cases	325				

a. 0 cells (0,0%) have expected count less than 5. The minimum expected count is 10,86.
b. Computed only for a 2x2 table

Symmetric Measures

		Value	Approximate Significance
Nominal by Nominal	Phi	,171	,002
	Cramer's V	,171	,002
	Contingency Coefficient	,168	,002
N of Valid Cases		325	

toilet_training * ui_athletics

Crosstab

			ui_athletics		
			no	yes	Total
toilet_training	no	Count	188_a	20_b	208
		Expected Count	181,1	26,9	208,0
		% within toilet_training	90,4%	9,6%	100,0%
		% within ui_athletics	66,4%	47,6%	64,0%
		% of Total	57,8%	6,2%	64,0%
		Standardized Residual	,5	-1,3	
	yes	Count	95_a	22_b	117
		Expected Count	101,9	15,1	117,0
		% within toilet_training	81,2%	18,8%	100,0%
		% within ui_athletics	33,6%	52,4%	36,0%
		% of Total	29,2%	6,8%	36,0%
		Standardized Residual	-,7	1,8	
Total		Count	283	42	325
		Expected Count	283,0	42,0	325,0
		% within toilet_training	87,1%	12,9%	100,0%
		% within ui_athletics	100,0%	100,0%	100,0%
		% of Total	87,1%	12,9%	100,0%

Each subscript letter denotes a subset of ui_athletics categories whose column proportions do not differ significantly from each other at the ,05 level.

Chi-Square Tests

	Value	df	Asymptotic Significance (2-sided)	Exact Sig. (2-sided)	Exact Sig. (1-sided)
Pearson Chi-Square	5,617[a]	1	,018		
Continuity Correction[b]	4,831	1	,028		
Likelihood Ratio	5,409	1	,020		
Fisher's Exact Test				,024	,015
Linear-by-Linear Association	5,600	1	,018		
N of Valid Cases	325				

a. 0 cells (0,0%) have expected count less than 5. The minimum expected count is 15,12.
b. Computed only for a 2x2 table

Symmetric Measures

		Value	Approximate Significance
Nominal by Nominal	Phi	,131	,018
	Cramer's V	,131	,018
	Contingency Coefficient	,130	,018
N of Valid Cases		325	

toilet_competition * ui_athletics

Crosstab

			ui_athletics		
			no	yes	Total
toilet_competition	no	Count	170$_a$	23$_a$	193
		Expected Count	168,1	24,9	193,0
		% within toilet_competition	88,1%	11,9%	100,0%
		% within ui_athletics	60,1%	54,8%	59,4%
		% of Total	52,3%	7,1%	59,4%
		Standardized Residual	,1	-,4	
	yes	Count	113$_a$	19$_a$	132
		Expected Count	114,9	17,1	132,0
		% within toilet_competition	85,6%	14,4%	100,0%
		% within ui_athletics	39,9%	45,2%	40,6%
		% of Total	34,8%	5,8%	40,6%
		Standardized Residual	-,2	,5	
Total		Count	283	42	325
		Expected Count	283,0	42,0	325,0
		% within toilet_competition	87,1%	12,9%	100,0%
		% within ui_athletics	100,0%	100,0%	100,0%
		% of Total	87,1%	12,9%	100,0%

Each subscript letter denotes a subset of ui_athletics categories whose column proportions do not differ significantly from each other at the ,05 level.

Chi-Square Tests

	Value	df	Asymptotic Significance (2-sided)	Exact Sig. (2-sided)	Exact Sig. (1-sided)
Pearson Chi-Square	,427[a]	1	,513		
Continuity Correction[b]	,236	1	,627		
Likelihood Ratio	,424	1	,515		
Fisher's Exact Test				,507	,312
Linear-by-Linear Association	,426	1	,514		
N of Valid Cases	325				

a. 0 cells (0,0%) have expected count less than 5. The minimum expected count is 17,06.
b. Computed only for a 2x2 table

Symmetric Measures

		Value	Approximate Significance
Nominal by Nominal	Phi	,036	,513
	Cramer's V	,036	,513
	Contingency Coefficient	,036	,513

N of Valid Cases	325	

caffeine * ui_athletics

Crosstab

			ui_athletics		Total
			no	yes	
caffeine	no	Count	193a	24a	217
		Expected Count	189,0	28,0	217,0
		% within caffeine	88,9%	11,1%	100,0%
		% within ui_athletics	68,2%	57,1%	66,8%
		% of Total	59,4%	7,4%	66,8%
		Standardized Residual	,3	-,8	
	yes	Count	90a	18a	108
		Expected Count	94,0	14,0	108,0
		% within caffeine	83,3%	16,7%	100,0%
		% within ui_athletics	31,8%	42,9%	33,2%
		% of Total	27,7%	5,5%	33,2%
		Standardized Residual	-,4	1,1	
Total		Count	283	42	325
		Expected Count	283,0	42,0	325,0
		% within caffeine	87,1%	12,9%	100,0%
		% within ui_athletics	100,0%	100,0%	100,0%
		% of Total	87,1%	12,9%	100,0%

Each subscript letter denotes a subset of ui_athletics categories whose column proportions do not differ significantly from each other at the ,05 level.

Chi-Square Tests

	Value	df	Asymptotic Significance (2-sided)	Exact Sig. (2-sided)	Exact Sig. (1-sided)
Pearson Chi-Square	2,014[a]	1	,156		
Continuity Correction[b]	1,547	1	,214		
Likelihood Ratio	1,948	1	,163		
Fisher's Exact Test				,164	,108
Linear-by-Linear Association	2,008	1	,156		
N of Valid Cases	325				

a. 0 cells (0,0%) have expected count less than 5. The minimum expected count is 13,96.
b. Computed only for a 2x2 table

Symmetric Measures

		Value	Approximate Significance
Nominal by Nominal	Phi	,079	,156
	Cramer's V	,079	,156
	Contingency Coefficient	,078	,156
N of Valid Cases		325	

event=combined events * ui_athletics

Crosstab

			ui_athletics		Total
			no	yes	
event=combined events	others	Count	277a	41a	318
		Expected Count	276,9	41,1	318,0
		% within event=combined events	87,1%	12,9%	100,0%
		% within ui_athletics	97,9%	97,6%	97,8%
		% of Total	85,2%	12,6%	97,8%
		Standardized Residual	,0	,0	
	combined	Count	6a	1a	7
		Expected Count	6,1	,9	7,0
		% within event=combined events	85,7%	14,3%	100,0%
		% within ui_athletics	2,1%	2,4%	2,2%

		% of Total	1,8%	0,3%	2,2%
		Standardized Residual	,0	,1	
Total		Count	283	42	325
		Expected Count	283,0	42,0	325,0
		% within event=combined events	87,1%	12,9%	100,0%
		% within ui_athletics	100,0%	100,0%	100,0%
		% of Total	87,1%	12,9%	100,0%

Each subscript letter denotes a subset of ui_athletics categories whose column proportions do not differ significantly from each other at the ,05 level.

Chi-Square Tests

	Value	df	Asymptotic Significance (2-sided)	Exact Sig. (2-sided)	Exact Sig. (1-sided)
Pearson Chi-Square	,012[a]	1	,913		
Continuity Correction[b]	,000	1	1,000		
Likelihood Ratio	,011	1	,915		
Fisher's Exact Test				1,000	,624
Linear-by-Linear Association	,012	1	,914		
N of Valid Cases	325				

a. 1 cells (25,0%) have expected count less than 5. The minimum expected count is ,90.
b. Computed only for a 2x2 table

Symmetric Measures

		Value	Approximate Significance
Nominal by Nominal	Phi	,006	,913
	Cramer's V	,006	,913
	Contingency Coefficient	,006	,913
N of Valid Cases		325	

event=hurdles * ui_athletics

Crosstab

			ui_athletics		
			no	yes	Total
event=hurdles	others	Count	250_a	36_a	286
		Expected Count	249,0	37,0	286,0
		% within event=hurdles	87,4%	12,6%	100,0%
		% within ui_athletics	88,3%	85,7%	88,0%
		% of Total	76,9%	11,1%	88,0%
		Standardized Residual	,1	-,2	
	hurdles	Count	33_a	6_a	39
		Expected Count	34,0	5,0	39,0
		% within event=hurdles	84,6%	15,4%	100,0%
		% within ui_athletics	11,7%	14,3%	12,0%
		% of Total	10,2%	1,8%	12,0%
		Standardized Residual	-,2	,4	
Total		Count	283	42	325
		Expected Count	283,0	42,0	325,0
		% within event=hurdles	87,1%	12,9%	100,0%
		% within ui_athletics	100,0%	100,0%	100,0%
		% of Total	87,1%	12,9%	100,0%

Each subscript letter denotes a subset of ui_athletics categories whose column proportions do not differ significantly from each other at the ,05 level.

Chi-Square Tests

	Value	df	Asymptotic Significance (2-sided)	Exact Sig. (2-sided)	Exact Sig. (1-sided)
Pearson Chi-Square	,239[a]	1	,625		
Continuity Correction[b]	,055	1	,815		
Likelihood Ratio	,228	1	,633		
Fisher's Exact Test				,613	,390
Linear-by-Linear Association	,238	1	,626		

N of Valid Cases	325				

a. 0 cells (0,0%) have expected count less than 5. The minimum expected count is 5,04.
b. Computed only for a 2x2 table

Symmetric Measures

		Value	Approximate Significance
Nominal by Nominal	Phi	,027	,625
	Cramer's V	,027	,625
	Contingency Coefficient	,027	,625
N of Valid Cases		325	

event=jumps * ui_athletics

Crosstab

			ui_athletics		
			no	yes	Total
event=jumps	others	Count	253_a	34_a	287
		Expected Count	249,9	37,1	287,0
		% within event=jumps	88,2%	11,8%	100,0%
		% within ui_athletics	89,4%	81,0%	88,3%
		% of Total	77,8%	10,5%	88,3%
		Standardized Residual	,2	-,5	
	jumps	Count	30_a	8_a	38
		Expected Count	33,1	4,9	38,0
		% within event=jumps	78,9%	21,1%	100,0%
		% within ui_athletics	10,6%	19,0%	11,7%
		% of Total	9,2%	2,5%	11,7%
		Standardized Residual	-,5	1,4	
Total		Count	283	42	325
		Expected Count	283,0	42,0	325,0
		% within event=jumps	87,1%	12,9%	100,0%
		% within ui_athletics	100,0%	100,0%	100,0%
		% of Total	87,1%	12,9%	100,0%

Each subscript letter denotes a subset of ui_athletics categories whose column proportions do not differ significantly from each other at the ,05 level.

Chi-Square Tests

	Value	df	Asymptotic Significance (2-sided)	Exact Sig. (2-sided)	Exact Sig. (1-sided)
Pearson Chi-Square	2,527[a]	1	,112		
Continuity Correction[b]	1,775	1	,183		
Likelihood Ratio	2,230	1	,135		
Fisher's Exact Test				,123	,096
Linear-by-Linear Association	2,519	1	,112		
N of Valid Cases	325				

a. 1 cells (25,0%) have expected count less than 5. The minimum expected count is 4,91.
b. Computed only for a 2x2 table

Symmetric Measures

		Value	Approximate Significance
Nominal by Nominal	Phi	,088	,112
	Cramer's V	,088	,112
	Contingency Coefficient	,088	,112
N of Valid Cases		325	

event=long distance runs * ui_athletics

Crosstab

			ui_athletics		
			no	yes	Total
event=long distance runs	others	Count	267a	39a	306
		Expected Count	266,5	39,5	306,0
		% within event=long distance runs	87,3%	12,7%	100,0%
		% within ui_athletics	94,3%	92,9%	94,2%
		% of Total	82,2%	12,0%	94,2%
		Standardized Residual	,0	-,1	
	long distance	Count	16a	3a	19
		Expected Count	16,5	2,5	19,0
		% within event=long distance runs	84,2%	15,8%	100,0%
		% within ui_athletics	5,7%	7,1%	5,8%
		% of Total	4,9%	0,9%	5,8%
		Standardized Residual	-,1	,3	
Total		Count	283	42	325
		Expected Count	283,0	42,0	325,0
		% within event=long distance runs	87,1%	12,9%	100,0%
		% within ui_athletics	100,0%	100,0%	100,0%
		% of Total	87,1%	12,9%	100,0%

Each subscript letter denotes a subset of ui_athletics categories whose column proportions do not differ significantly from each other at the ,05 level.

Chi-Square Tests

	Value	df	Asymptotic Significance (2-sided)	Exact Sig. (2-sided)	Exact Sig. (1-sided)
Pearson Chi-Square	,147[a]	1	,701		
Continuity Correction[b]	,001	1	,975		
Likelihood Ratio	,140	1	,709		
Fisher's Exact Test				,722	,455
Linear-by-Linear Association	,147	1	,702		
N of Valid Cases	325				

a. 1 cells (25,0%) have expected count less than 5. The minimum expected count is 2,46.
b. Computed only for a 2x2 table

Symmetric Measures

		Value	Approximate Significance
Nominal by Nominal	Phi	,021	,701
	Cramer's V	,021	,701
	Contingency Coefficient	,021	,701
N of Valid Cases		325	

event=middle distance runs * ui_athletics

Crosstab

			ui_athletics	
			no	yes
event=middle distance runs	others	Count	254_a	32_b
		Expected Count	249,0	37,0
		% within event=middle distance runs	88,8%	11,2%
		% within ui_athletics	89,8%	76,2%
		% of Total	78,2%	9,8%
		Standardized Residual	,3	-,8
	middle distance	Count	29_a	10_b
		Expected Count	34,0	5,0
		% within event=middle distance runs	74,4%	25,6%
		% within ui_athletics	10,2%	23,8%
		% of Total	8,9%	3,1%
		Standardized Residual	-,9	2,2
Total		Count	283	42
		Expected Count	283,0	42,0
		% within event=middle distance runs	87,1%	12,9%
		% within ui_athletics	100,0%	100,0%
		% of Total	87,1%	12,9%

Crosstab

			Total
event=middle distance runs	others	Count	286
		Expected Count	286,0
		% within event=middle distance runs	100,0%
		% within ui_athletics	88,0%
		% of Total	88,0%
		Standardized Residual	
	middle distance	Count	39
		Expected Count	39,0
		% within event=middle distance runs	100,0%
		% within ui_athletics	12,0%
		% of Total	12,0%
		Standardized Residual	
Total		Count	325
		Expected Count	325,0
		% within event=middle distance runs	100,0%
		% within ui_athletics	100,0%
		% of Total	100,0%

Each subscript letter denotes a subset of ui_athletics categories whose column proportions do not differ significantly from each other at the ,05 level.

Chi-Square Tests

	Value	df	Asymptotic Significance (2-sided)	Exact Sig. (2-sided)	Exact Sig. (1-sided)
Pearson Chi-Square	6,370[a]	1	,012		
Continuity Correction[b]	5,151	1	,023		
Likelihood Ratio	5,342	1	,021		
Fisher's Exact Test				,020	,017

Linear-by-Linear Association	6,351	1	,012		
N of Valid Cases	325				

a. 0 cells (0,0%) have expected count less than 5. The minimum expected count is 5,04.
b. Computed only for a 2x2 table

Symmetric Measures

		Value	Approximate Significance
Nominal by Nominal	Phi	,140	,012
	Cramer's V	,140	,012
	Contingency Coefficient	,139	,012
N of Valid Cases		325	

event=race walking * ui_athletics

Crosstab

			ui_athletics		Total
			no	yes	
event=race walking	others	Count	261_a	38_a	299
		Expected Count	260,4	38,6	299,0
		% within event=race walking	87,3%	12,7%	100,0%
		% within ui_athletics	92,2%	90,5%	92,0%
		% of Total	80,3%	11,7%	92,0%
		Standardized Residual	,0	-,1	
	race walking	Count	22_a	4_a	26
		Expected Count	22,6	3,4	26,0
		% within event=race walking	84,6%	15,4%	100,0%
		% within ui_athletics	7,8%	9,5%	8,0%
		% of Total	6,8%	1,2%	8,0%
		Standardized Residual	-,1	,3	
Total		Count	283	42	325
		Expected Count	283,0	42,0	325,0
		% within event=race walking	87,1%	12,9%	100,0%
		% within ui_athletics	100,0%	100,0%	100,0%
		% of Total	87,1%	12,9%	100,0%

Each subscript letter denotes a subset of ui_athletics categories whose column proportions do not differ significantly from each other at the ,05 level.

Chi-Square Tests

	Value	df	Asymptotic Significance (2-sided)	Exact Sig. (2-sided)	Exact Sig. (1-sided)
Pearson Chi-Square	,152[a]	1	,696		
Continuity Correction[b]	,007	1	,932		
Likelihood Ratio	,145	1	,703		
Fisher's Exact Test				,759	,440
Linear-by-Linear Association	,152	1	,697		
N of Valid Cases	325				

a. 1 cells (25,0%) have expected count less than 5. The minimum expected count is 3,36.
b. Computed only for a 2x2 table

Symmetric Measures

		Value	Approximate Significance
Nominal by Nominal	Phi	,022	,696
	Cramer's V	,022	,696
	Contingency Coefficient	,022	,696
N of Valid Cases		325	

event=throws * ui_athletics

Crosstab

ui_athletics	Total

			no	yes	
event=throws	others	Count	255a	41a	296
		Expected Count	257,7	38,3	296,0
		% within event=throws	86,1%	13,9%	100,0%
		% within ui_athletics	90,1%	97,6%	91,1%
		% of Total	78,5%	12,6%	91,1%
		Standardized Residual	-,2	,4	
	throws	Count	28a	1a	29
		Expected Count	25,3	3,7	29,0
		% within event=throws	96,6%	3,4%	100,0%
		% within ui_athletics	9,9%	2,4%	8,9%
		% of Total	8,6%	0,3%	8,9%
		Standardized Residual	,5	-1,4	
Total		Count	283	42	325
		Expected Count	283,0	42,0	325,0
		% within event=throws	87,1%	12,9%	100,0%
		% within ui_athletics	100,0%	100,0%	100,0%
		% of Total	87,1%	12,9%	100,0%

Each subscript letter denotes a subset of ui_athletics categories whose column proportions do not differ significantly from each other at the ,05 level.

Chi-Square Tests

	Value	df	Asymptotic Significance (2-sided)	Exact Sig. (2-sided)	Exact Sig. (1-sided)
Pearson Chi-Square	2,540[a]	1	,111		
Continuity Correction[b]	1,700	1	,192		
Likelihood Ratio	3,364	1	,067		
Fisher's Exact Test				,148	,086
Linear-by-Linear Association	2,532	1	,112		
N of Valid Cases	325				

a. 1 cells (25,0%) have expected count less than 5. The minimum expected count is 3,75.
b. Computed only for a 2x2 table

Symmetric Measures

		Value	Approximate Significance
Nominal by Nominal	Phi	-,088	,111
	Cramer's V	,088	,111
	Contingency Coefficient	,088	,111
N of Valid Cases		325	

health_conditions=constipation * ui_athletics

Crosstab

			ui_athletics	
			no	yes
health_conditions=constipation	others	Count	279a	40a
		Expected Count	277,8	41,2
		% within health_conditions=constipation	87,5%	12,5%
		% within ui_athletics	98,6%	95,2%
		% of Total	85,8%	12,3%
		Standardized Residual	,1	-,2
	constipations	Count	4a	2a
		Expected Count	5,2	,8
		% within health_conditions=constipation	66,7%	33,3%
		% within ui_athletics	1,4%	4,8%
		% of Total	1,2%	0,6%
		Standardized Residual	-,5	1,4
Total		Count	283	42
		Expected Count	283,0	42,0
		% within health_conditions=constipation	87,1%	12,9%

		% within ui_athletics	100,0%	100,0%
		% of Total	87,1%	12,9%

Crosstab

			Total
health_conditions=constipation	others	Count	319
		Expected Count	319,0
		% within health_conditions=constipation	100,0%
		% within ui_athletics	98,2%
		% of Total	98,2%
		Standardized Residual	
	constipations	Count	6
		Expected Count	6,0
		% within health_conditions=constipation	100,0%
		% within ui_athletics	1,8%
		% of Total	1,8%
		Standardized Residual	
Total		Count	325
		Expected Count	325,0
		% within health_conditions=constipation	100,0%
		% within ui_athletics	100,0%
		% of Total	100,0%

Each subscript letter denotes a subset of ui_athletics categories whose column proportions do not differ significantly from each other at the ,05 level.

Chi-Square Tests

	Value	df	Asymptotic Significance (2-sided)	Exact Sig. (2-sided)	Exact Sig. (1-sided)
Pearson Chi-Square	2,263[a]	1	,133		
Continuity Correction[b]	,792	1	,373		
Likelihood Ratio	1,696	1	,193		
Fisher's Exact Test				,175	,175
Linear-by-Linear Association	2,256	1	,133		
N of Valid Cases	325				

a. 1 cells (25,0%) have expected count less than 5. The minimum expected count is ,78.
b. Computed only for a 2x2 table

Symmetric Measures

		Value	Approximate Significance
Nominal by Nominal	Phi	,083	,133
	Cramer's V	,083	,133
	Contingency Coefficient	,083	,133
N of Valid Cases		325	

health_conditions=diabetes * ui_athletics

Crosstab

			ui_athletics		
			no	yes	Total
health_conditions=diabetes	others	Count	282_a	42_a	324
		Expected Count	282,1	41,9	324,0
		% within health_conditions=diabetes	87,0%	13,0%	100,0%
		% within ui_athletics	99,6%	100,0%	99,7%
		% of Total	86,8%	12,9%	99,7%

		Standardized Residual	,0	,0	
	diabetes	Count	1_a	0_a	1
		Expected Count	,9	,1	1,0
		% within health_conditions=diabetes	100,0%	0,0%	100,0%
		% within ui_athletics	0,4%	0,0%	0,3%
		% of Total	0,3%	0,0%	0,3%
		Standardized Residual	,1	-,4	
Total		Count	283	42	325
		Expected Count	283,0	42,0	325,0
		% within health_conditions=diabetes	87,1%	12,9%	100,0%
		% within ui_athletics	100,0%	100,0%	100,0%
		% of Total	87,1%	12,9%	100,0%

Each subscript letter denotes a subset of ui_athletics categories whose column proportions do not differ significantly from each other at the ,05 level.

Chi-Square Tests

	Value	df	Asymptotic Significance (2-sided)	Exact Sig. (2-sided)	Exact Sig. (1-sided)
Pearson Chi-Square	,149[a]	1	,700		
Continuity Correction[b]	,000	1	1,000		
Likelihood Ratio	,277	1	,599		
Fisher's Exact Test				1,000	,871
Linear-by-Linear Association	,148	1	,700		
N of Valid Cases	325				

a. 2 cells (50,0%) have expected count less than 5. The minimum expected count is ,13.
b. Computed only for a 2x2 table

Symmetric Measures

		Value	Approximate Significance
Nominal by Nominal	Phi	-,021	,700
	Cramer's V	,021	,700
	Contingency Coefficient	,021	,700
N of Valid Cases		325	

health_conditions=frequent UI * ui_athletics

Crosstab

			ui_athletics	
			no	yes
health_conditions=frequent UI	others	Count	276_a	38_b
		Expected Count	273,4	40,6
		% within health_conditions=frequent UI	87,9%	12,1%
		% within ui_athletics	97,5%	90,5%
		% of Total	84,9%	11,7%
		Standardized Residual	,2	-,4
	Frequent UI	Count	7_a	4_b
		Expected Count	9,6	1,4
		% within health_conditions=frequent UI	63,6%	36,4%
		% within ui_athletics	2,5%	9,5%
		% of Total	2,2%	1,2%
		Standardized Residual	-,8	2,2
Total		Count	283	42
		Expected Count	283,0	42,0
		% within health_conditions=frequent UI	87,1%	12,9%
		% within ui_athletics	100,0%	100,0%
		% of Total	87,1%	12,9%

Crosstab

			Total
health_conditions=frequent UI	others	Count	314
		Expected Count	314,0
		% within health_conditions=frequent UI	100,0%
		% within ui_athletics	96,6%
		% of Total	96,6%
		Standardized Residual	
	Frequent UI	Count	11
		Expected Count	11,0
		% within health_conditions=frequent UI	100,0%
		% within ui_athletics	3,4%
		% of Total	3,4%
		Standardized Residual	
Total		Count	325
		Expected Count	325,0
		% within health_conditions=frequent UI	100,0%
		% within ui_athletics	100,0%
		% of Total	100,0%

Each subscript letter denotes a subset of ui_athletics categories whose column proportions do not differ significantly from each other at the ,05 level.

Chi-Square Tests

	Value	df	Asymptotic Significance (2-sided)	Exact Sig. (2-sided)	Exact Sig. (1-sided)
Pearson Chi-Square	5,559[a]	1	,018		
Continuity Correction[b]	3,612	1	,057		
Likelihood Ratio	4,078	1	,043		
Fisher's Exact Test				,041	,041
Linear-by-Linear Association	5,542	1	,019		
N of Valid Cases	325				

a. 1 cells (25,0%) have expected count less than 5. The minimum expected count is 1,42.
b. Computed only for a 2x2 table

Symmetric Measures

		Value	Approximate Significance
Nominal by Nominal	Phi	,131	,018
	Cramer's V	,131	,018
	Contingency Coefficient	,130	,018
N of Valid Cases		325	

health_conditions=heart arrhytmia * ui_athletics

Crosstab

			ui_athletics no	ui_athletics yes
health_conditions=heart arrhytmia	others	Count	282_a	42_a
		Expected Count	282,1	41,9
		% within health_conditions=heart arrhytmia	87,0%	13,0%
		% within ui_athletics	99,6%	100,0%
		% of Total	86,8%	12,9%
		Standardized Residual	,0	,0
	heart arrhytmia	Count	1_a	0_a
		Expected Count	,9	,1
		% within health_conditions=heart arrhytmia	100,0%	0,0%
		% within ui_athletics	0,4%	0,0%

		% of Total	0,3%	0,0%
		Standardized Residual	,1	-,4
Total		Count	283	42
		Expected Count	283,0	42,0
		% within health_conditions=heart arrhytmia	87,1%	12,9%
		% within ui_athletics	100,0%	100,0%
		% of Total	87,1%	12,9%

Crosstab

			Total
health_conditions=heart arrhytmia	others	Count	324
		Expected Count	324,0
		% within health_conditions=heart arrhytmia	100,0%
		% within ui_athletics	99,7%
		% of Total	99,7%
		Standardized Residual	
	heart arrhytmia	Count	1
		Expected Count	1,0
		% within health_conditions=heart arrhytmia	100,0%
		% within ui_athletics	0,3%
		% of Total	0,3%
		Standardized Residual	
Total		Count	325
		Expected Count	325,0
		% within health_conditions=heart arrhytmia	100,0%
		% within ui_athletics	100,0%
		% of Total	100,0%

Each subscript letter denotes a subset of ui_athletics categories whose column proportions do not differ significantly from each other at the ,05 level.

Chi-Square Tests

	Value	df	Asymptotic Significance (2-sided)	Exact Sig. (2-sided)	Exact Sig. (1-sided)
Pearson Chi-Square	,149[a]	1	,700		
Continuity Correction[b]	,000	1	1,000		
Likelihood Ratio	,277	1	,599		
Fisher's Exact Test				1,000	,871
Linear-by-Linear Association	,148	1	,700		
N of Valid Cases	325				

a. 2 cells (50,0%) have expected count less than 5. The minimum expected count is ,13.
b. Computed only for a 2x2 table

Symmetric Measures

		Value	Approximate Significance
Nominal by Nominal	Phi	-,021	,700
	Cramer's V	,021	,700
	Contingency Coefficient	,021	,700
N of Valid Cases		325	

health_conditions=pelvic surgery * ui_athletics

Crosstab

			ui_athletics	
			no	yes
health_conditions=pelvic surgery	others	Count	282a	41a
		Expected Count	281,3	41,7
		% within health_conditions=pelvic surgery	87,3%	12,7%
		% within ui_athletics	99,6%	97,6%
		% of Total	86,8%	12,6%
		Standardized Residual	,0	-,1
	pelvic surgery	Count	1a	1a
		Expected Count	1,7	,3
		% within health_conditions=pelvic surgery	50,0%	50,0%
		% within ui_athletics	0,4%	2,4%
		% of Total	0,3%	0,3%
		Standardized Residual	-,6	1,5
Total		Count	283	42
		Expected Count	283,0	42,0
		% within health_conditions=pelvic surgery	87,1%	12,9%
		% within ui_athletics	100,0%	100,0%
		% of Total	87,1%	12,9%

Crosstab

			Total
health_conditions=pelvic surgery	others	Count	323
		Expected Count	323,0
		% within health_conditions=pelvic surgery	100,0%
		% within ui_athletics	99,4%
		% of Total	99,4%
		Standardized Residual	
	pelvic surgery	Count	2
		Expected Count	2,0
		% within health_conditions=pelvic surgery	100,0%
		% within ui_athletics	0,6%
		% of Total	0,6%
		Standardized Residual	
Total		Count	325
		Expected Count	325,0
		% within health_conditions=pelvic surgery	100,0%
		% within ui_athletics	100,0%
		% of Total	100,0%

Each subscript letter denotes a subset of ui_athletics categories whose column proportions do not differ significantly from each other at the ,05 level.

Chi-Square Tests

	Value	df	Asymptotic Significance (2-sided)	Exact Sig. (2-sided)	Exact Sig. (1-sided)
Pearson Chi-Square	2,458[a]	1	,117		
Continuity Correction[b]	,261	1	,610		
Likelihood Ratio	1,612	1	,204		
Fisher's Exact Test				,242	,242
Linear-by-Linear Association	2,451	1	,117		

N of Valid Cases	325				

a. 2 cells (50,0%) have expected count less than 5. The minimum expected count is ,26.
b. Computed only for a 2x2 table

Symmetric Measures

		Value	Approximate Significance
Nominal by Nominal	Phi	,087	,117
	Cramer's V	,087	,117
	Contingency Coefficient	,087	,117
N of Valid Cases		325	

health_conditions=hypertension * ui_athletics

Crosstab

			ui_athletics	
			no	yes
health_conditions=hypertension	others	Count	282_a	41_a
		Expected Count	281,3	41,7
		% within health_conditions=hypertension	87,3%	12,7%
		% within ui_athletics	99,6%	97,6%
		% of Total	86,8%	12,6%
		Standardized Residual	,0	-,1
	hypertension	Count	1_a	1_a
		Expected Count	1,7	,3
		% within health_conditions=hypertension	50,0%	50,0%
		% within ui_athletics	0,4%	2,4%
		% of Total	0,3%	0,3%
		Standardized Residual	-,6	1,5
Total		Count	283	42
		Expected Count	283,0	42,0
		% within health_conditions=hypertension	87,1%	12,9%
		% within ui_athletics	100,0%	100,0%
		% of Total	87,1%	12,9%

Crosstab

			Total
health_conditions=hypertension	others	Count	323
		Expected Count	323,0
		% within health_conditions=hypertension	100,0%
		% within ui_athletics	99,4%
		% of Total	99,4%
		Standardized Residual	
	hypertension	Count	2
		Expected Count	2,0
		% within health_conditions=hypertension	100,0%
		% within ui_athletics	0,6%
		% of Total	0,6%
		Standardized Residual	
Total		Count	325
		Expected Count	325,0
		% within health_conditions=hypertension	100,0%
		% within ui_athletics	100,0%
		% of Total	100,0%

Each subscript letter denotes a subset of ui_athletics categories whose column proportions do not differ significantly from each other at the ,05 level.

Chi-Square Tests

	Value	df	Asymptotic Significance (2-sided)	Exact Sig. (2-sided)	Exact Sig. (1-sided)
Pearson Chi-Square	2,458[a]	1	,117		
Continuity Correction[b]	,261	1	,610		
Likelihood Ratio	1,612	1	,204		
Fisher's Exact Test				,242	,242
Linear-by-Linear Association	2,451	1	,117		
N of Valid Cases	325				

a. 2 cells (50,0%) have expected count less than 5. The minimum expected count is ,26.
b. Computed only for a 2x2 table

Symmetric Measures

		Value	Approximate Significance
Nominal by Nominal	Phi	,087	,117
	Cramer's V	,087	,117
	Contingency Coefficient	,087	,117
N of Valid Cases		325	

health_conditions=hyperthyrodism * ui_athletics

Crosstab

			ui_athletics	
			no	yes
health_conditions=hyperthyrodism	others	Count	282_a	42_a
		Expected Count	282,1	41,9
		% within health_conditions=hyperthyrodism	87,0%	13,0%
		% within ui_athletics	99,6%	100,0%
		% of Total	86,8%	12,9%
		Standardized Residual	,0	,0
	hyperthyrodism	Count	1_a	0_a
		Expected Count	,9	,1
		% within health_conditions=hyperthyrodism	100,0%	0,0%
		% within ui_athletics	0,4%	0,0%
		% of Total	0,3%	0,0%
		Standardized Residual	,1	-,4
Total		Count	283	42
		Expected Count	283,0	42,0
		% within health_conditions=hyperthyrodism	87,1%	12,9%
		% within ui_athletics	100,0%	100,0%
		% of Total	87,1%	12,9%

Crosstab

			Total
health_conditions=hyperthyrodism	others	Count	324
		Expected Count	324,0
		% within health_conditions=hyperthyrodism	100,0%
		% within ui_athletics	99,7%
		% of Total	99,7%
		Standardized Residual	
	hyperthyrodism	Count	1
		Expected Count	1,0

		% within health_conditions=hyperthyrodism	100,0%
		% within ui_athletics	0,3%
		% of Total	0,3%
		Standardized Residual	
Total		Count	325
		Expected Count	325,0
		% within health_conditions=hyperthyrodism	100,0%
		% within ui_athletics	100,0%
		% of Total	100,0%

Each subscript letter denotes a subset of ui_athletics categories whose column proportions do not differ significantly from each other at the ,05 level.

Chi-Square Tests

	Value	df	Asymptotic Significance (2-sided)	Exact Sig. (2-sided)	Exact Sig. (1-sided)
Pearson Chi-Square	,149[a]	1	,700		
Continuity Correction[b]	,000	1	1,000		
Likelihood Ratio	,277	1	,599		
Fisher's Exact Test				1,000	,871
Linear-by-Linear Association	,148	1	,700		
N of Valid Cases	325				

a. 2 cells (50,0%) have expected count less than 5. The minimum expected count is ,13.
b. Computed only for a 2x2 table

Symmetric Measures

		Value	Approximate Significance
Nominal by Nominal	Phi	-,021	,700
	Cramer's V	,021	,700
	Contingency Coefficient	,021	,700
N of Valid Cases		325	

health_conditions=respiratory issues * ui_athletics

Crosstab

			ui_athletics	
			no	yes
health_conditions=respiratory issues	others	Count	261_a	39_a
		Expected Count	261,2	38,8
		% within health_conditions=respiratory issues	87,0%	13,0%
		% within ui_athletics	92,2%	92,9%
		% of Total	80,3%	12,0%
		Standardized Residual	,0	,0
	respiratory issues	Count	22_a	3_a
		Expected Count	21,8	3,2
		% within health_conditions=respiratory issues	88,0%	12,0%
		% within ui_athletics	7,8%	7,1%
		% of Total	6,8%	0,9%
		Standardized Residual	,0	-,1
Total		Count	283	42
		Expected Count	283,0	42,0
		% within health_conditions=respiratory issues	87,1%	12,9%
		% within ui_athletics	100,0%	100,0%
		% of Total	87,1%	12,9%

Crosstab

			Total
health_conditions=respiratory issues	others	Count	300
		Expected Count	300,0
		% within health_conditions=respiratory issues	100,0%
		% within ui_athletics	92,3%
		% of Total	92,3%
		Standardized Residual	
	respiratory issues	Count	25
		Expected Count	25,0
		% within health_conditions=respiratory issues	100,0%
		% within ui_athletics	7,7%
		% of Total	7,7%
		Standardized Residual	
Total		Count	325
		Expected Count	325,0
		% within health_conditions=respiratory issues	100,0%
		% within ui_athletics	100,0%
		% of Total	100,0%

Each subscript letter denotes a subset of ui_athletics categories whose column proportions do not differ significantly from each other at the ,05 level.

Chi-Square Tests

	Value	df	Asymptotic Significance (2-sided)	Exact Sig. (2-sided)	Exact Sig. (1-sided)
Pearson Chi-Square	,021[a]	1	,886		
Continuity Correction[b]	,000	1	1,000		
Likelihood Ratio	,021	1	,885		
Fisher's Exact Test				1,000	,591
Linear-by-Linear Association	,020	1	,886		
N of Valid Cases	325				

a. 1 cells (25,0%) have expected count less than 5. The minimum expected count is 3,23.
b. Computed only for a 2x2 table

Symmetric Measures

		Value	Approximate Significance
Nominal by Nominal	Phi	-,008	,886
	Cramer's V	,008	,886
	Contingency Coefficient	,008	,886
N of Valid Cases		325	

stress_fractures=1 to 3 * ui_athletics

Crosstab

			ui_athletics		
			no	yes	Total
stress_fractures=1 to 3	others	Count	233_a	32_a	265
		Expected Count	230,8	34,2	265,0
		% within stress_fractures=1 to 3	87,9%	12,1%	100,0%
		% within ui_athletics	82,3%	76,2%	81,5%
		% of Total	71,7%	9,8%	81,5%
		Standardized Residual	,1	-,4	

	1 to 3	Count	50_a	10_a	60
		Expected Count	52,2	7,8	60,0
		% within stress_fractures=1 to 3	83,3%	16,7%	100,0%
		% within ui_athletics	17,7%	23,8%	18,5%
		% of Total	15,4%	3,1%	18,5%
		Standardized Residual	-,3	,8	
Total		Count	283	42	325
		Expected Count	283,0	42,0	325,0
		% within stress_fractures=1 to 3	87,1%	12,9%	100,0%
		% within ui_athletics	100,0%	100,0%	100,0%
		% of Total	87,1%	12,9%	100,0%

Each subscript letter denotes a subset of ui_athletics categories whose column proportions do not differ significantly from each other at the ,05 level.

Chi-Square Tests

	Value	df	Asymptotic Significance (2-sided)	Exact Sig. (2-sided)	Exact Sig. (1-sided)
Pearson Chi-Square	,916[a]	1	,338		
Continuity Correction[b]	,554	1	,457		
Likelihood Ratio	,866	1	,352		
Fisher's Exact Test				,393	,224
Linear-by-Linear Association	,914	1	,339		
N of Valid Cases	325				

a. 0 cells (0,0%) have expected count less than 5. The minimum expected count is 7,75.
b. Computed only for a 2x2 table

Symmetric Measures

		Value	Approximate Significance
Nominal by Nominal	Phi	,053	,338
	Cramer's V	,053	,338
	Contingency Coefficient	,053	,338
N of Valid Cases		325	

stress_fractures=more than 3 * ui_athletics

Crosstab

			ui_athletics	
			no	yes
stress_fractures=more than 3	others	Count	276_a	40_a
		Expected Count	275,2	40,8
		% within stress_fractures=more than 3	87,3%	12,7%
		% within ui_athletics	97,5%	95,2%
		% of Total	84,9%	12,3%
		Standardized Residual	,1	-,1
	more than 3	Count	7_a	2_a
		Expected Count	7,8	1,2
		% within stress_fractures=more than 3	77,8%	22,2%
		% within ui_athletics	2,5%	4,8%
		% of Total	2,2%	0,6%
		Standardized Residual	-,3	,8
Total		Count	283	42
		Expected Count	283,0	42,0
		% within stress_fractures=more than 3	87,1%	12,9%
		% within ui_athletics	100,0%	100,0%
		% of Total	87,1%	12,9%

Crosstab

			Total

stress_fractures=more than 3	others	Count	316
		Expected Count	316,0
		% within stress_fractures=more than 3	100,0%
		% within ui_athletics	97,2%
		% of Total	97,2%
		Standardized Residual	
	more than 3	Count	9
		Expected Count	9,0
		% within stress_fractures=more than 3	100,0%
		% within ui_athletics	2,8%
		% of Total	2,8%
		Standardized Residual	
Total		Count	325
		Expected Count	325,0
		% within stress_fractures=more than 3	100,0%
		% within ui_athletics	100,0%
		% of Total	100,0%

Each subscript letter denotes a subset of ui_athletics categories whose column proportions do not differ significantly from each other at the ,05 level.

Chi-Square Tests

	Value	df	Asymptotic Significance (2-sided)	Exact Sig. (2-sided)	Exact Sig. (1-sided)
Pearson Chi-Square	,711[a]	1	,399		
Continuity Correction[b]	,115	1	,734		
Likelihood Ratio	,607	1	,436		
Fisher's Exact Test				,328	,328
Linear-by-Linear Association	,709	1	,400		
N of Valid Cases	325				

a. 1 cells (25,0%) have expected count less than 5. The minimum expected count is 1,16.
b. Computed only for a 2x2 table

Symmetric Measures

		Value	Approximate Significance
Nominal by Nominal	Phi	,047	,399
	Cramer's V	,047	,399
	Contingency Coefficient	,047	,399
N of Valid Cases		325	
